# Comparing multi-criteria decision analysis and integrated assessment to support long-term water supply planning

**DOI:** 10.1371/journal.pone.0176663

**Published:** 2017-05-08

**Authors:** Lisa Scholten, Max Maurer, Judit Lienert

**Affiliations:** 1 Faculty of Civil Engineering and Geosciences, Delft University of Technology, Delft, The Netherlands; 2 Eawag: Swiss Federal Institute of Aquatic Science and Technology, Dübendorf, Switzerland; 3 ETH Zurich, Institute of Environmental Engineering, Zurich, Switzerland; US Army Engineer Research and Development Center, UNITED STATES

## Abstract

We compare the use of multi-criteria decision analysis (MCDA)–or more precisely, models used in multi-attribute value theory (MAVT)–to integrated assessment (IA) models for supporting long-term water supply planning in a small town case study in Switzerland. They are used to evaluate thirteen system scale water supply alternatives in four future scenarios regarding forty-four objectives, covering technical, social, environmental, and economic aspects. The alternatives encompass both conventional and unconventional solutions and differ regarding technical, spatial and organizational characteristics. This paper focuses on the impact assessment and final evaluation step of the structured MCDA decision support process. We analyze the performance of the alternatives for ten stakeholders. We demonstrate the implications of model assumptions by comparing two IA and three MAVT evaluation model layouts of different complexity. For this comparison, we focus on the validity (ranking stability), desirability (value), and distinguishability (value range) of the alternatives given the five model layouts. These layouts exclude or include stakeholder preferences and uncertainties. Even though all five led us to identify the same best alternatives, they did not produce identical rankings. We found that the MAVT-type models provide higher distinguishability and a more robust basis for discussion than the IA-type models. The needed complexity of the model, however, should be determined based on the intended use of the model within the decision support process. The best-performing alternatives had consistently strong performance for all stakeholders and future scenarios, whereas the current water supply system was outperformed in all evaluation layouts. The best-performing alternatives comprise proactive pipe rehabilitation, adapted firefighting provisions, and decentralized water storage and/or treatment. We present recommendations for possible ways of improving water supply planning in the case study and beyond.

## 1 Introduction

### 1.1 Water supply planning and uncertainty

*Strategic planning* refers to the decision-making process that conceptualizes and organizes activities to attain desired long-term objectives. Strategic decision-making is complex for many reasons: the presence of several decision makers and stakeholder groups, conflicting objectives and tough value tradeoffs, the challenge of identifying good alternatives and large uncertainties in estimating their performance, among others [1, 2]. Often, decision makers are required to justify their decisions to regulatory authorities, the public, or shareholders. This increases the need for a more holistic assessment, where expertise from different disciplines is integrated (e.g. [3, 4]).

These complexities also apply to water supply planning. In the scholarly discourse, the prevalent centralized urban water infrastructures are criticized for being unsustainable and for their limited ability to cope with dynamic socio-economic, demographic, and environmental change [5–9]. Concurrently, the long-lived existing system increasingly requires rehabilitation, involving high costs (e.g. [10, 11–13]). This rehabilitation need poses a financial and practical challenge, but could also be an opportunity for change, as assets do not necessarily need to be replaced like-for-like [14]. As a result, sustainability has become a leading concept in water supply planning (for a more detailed account of sustainability in the context of urban water see e.g. [5, 9, 15–17]). Many predominantly technical alternatives have been advocated, such as decentralized water treatment and recycling, rainwater harvesting, reduction of firefighting flows etc. [8, 18–20]. Organizational alternatives such as alternative forms of utility organization, including regionalization and privatization are sometimes suggested [21, 22].

Despite these propositions, alternatives to the current centralized system are not generally considered in practice, leading to an “innovation deficit” of urban water systems [23]. The failure to actively search for (and to include) innovative alternatives into the set of options has been recognized as one of the main hindrances to successful decision-making in a range of fields [24]. This and other hindrances have been attributed to socio-economic and institutional barriers, but also to planning itself, because it often neglects future uncertainties and excludes broader goals, important stakeholders, and alternative paths of action (e.g. in Australia, Switzerland, UK, and the USA, see [5, 21, 24, 25, 26, 27]). To overcome this deficit, more strategic, discursive, and collaborative infrastructure planning processes are demanded [21, 23, 28].

Multi-criteria decision analysis (MCDA) can deal with such complexities [2, 29, 30]. MCDA structures and decomposes complex decision problems into manageable tasks and supports the decision process with analytic models. As in other models, quantifiable uncertainties can be addressed by applying probability theory and uncertainty analysis to the modeling process [30–32]. Handling dynamic uncertain framework conditions is more difficult, as their impact on performance is often hardly quantifiable without making assumptions about possible future states (e.g. the effect of climate change on rainfall dynamics, which determine sewer design [33]). Scenario techniques have been developed to analyze the consequences of such uncertain future dynamics [34–37]. Scenarios are widely applied in strategic planning [34, 38], including water management [39] or in combination with MCDA [40–42].

### 1.2 Integrated assessment and multi-criteria decision analysis

Integrated assessment (IA) is commonly used to support organizational decision- and policy-making in the form of well-known benchmarks such as the Human Development Index [43] or more generic sustainability assessments [44, 45]. IA is mentioned with different labels such as integrated or sustainability assessment, indicator-based benchmarking, composite indicators, multi-criteria analysis etc. All these approaches aim to evaluate possible courses of action (alternatives, options) by condensing multiple dimensions into one or several indicators, thereby reducing complexity to a manageable amount of information [45]. Integrated assessment of water systems is also common [46–51]. Technical and environmental criteria are predominantly used, usually amounting to less than 10.

Like IA, MCDA aims to support the evaluation and selection of alternatives, but MCDA additionally integrates the values and preferences of the decision makers (or stakeholders) into the indicator assessment. There are many MCDA methods that build on different assumptions about the decision maker’s preferences, consequentially embracing different evaluation models to reflect these (see overviews in [52, 53]). MCDA uses a suite of evaluation models, but also seeks to offer a transparent process that leads to rational and justifiable decisions in which the model serves as a focus for discussion [52]. It has been applied to complex environmental planning problems in general [1, 54–56] and to water management in particular (e.g. [57, 58–62]).

Selecting an MCDA method is a challenge, given the lack of consensus about which methods are more suitable in a particular situation. A few authors have compared selected MCDA models in the same case study to investigate whether the resulting rankings of alternatives—and the derived recommendations—differ depending on the method. A comprehensive review is provided in Myšiak [63]. The results remain inconclusive. In some cases, different models led to the same and in some cases to different best alternatives. Since then, a few studies have been added [64–68], producing similar results. Several authors recommend to apply different models in parallel to increase the robustness of the results [63, 64], if not to use hybrid methods [67], yet this is only rarely done.

In public policy and decision-making, value-focused MCDA methods such as multi-attribute value theory (MAVT [69]) may be preferred due to several reasons, explained in e.g. Reichert, et al. [30] and Schuwirth, et al.[70]: 1) foundation on axioms of rational choice, 2) explicit handling of prediction uncertainty, 3) ability to process many alternatives without increased elicitation effort, and 4) possibility to include new alternatives at any stage of the decision process). Many benefits are ascribed to using a value-focused decision analytic process. These include—among others—a higher transparency of the intervention, models and assumptions made [52, 71, 72], making the impacts of alternatives tangible, reducing the likelihood of making poor decisions [73], and allowing to assess the value of gathering more information in case of uncertainty [2]. Significant cost savings [74] in the corporate sector have been reported. Frequently mentioned disadvantages are high time demands and the possibility that interventions may lose momentum (e.g. [1, 71, 75]). MAVT has been applied to a wide range of fields, including water management. While most of these applications concern water resources decisions (reviewed in e.g. [58]), a number of applications to urban water management have also been reported (e.g. [75, 76, 77–83]).

As compared to IA, these MAVT applications to urban water management typically cover a larger number and more diverse criteria, including technical, environmental, social, organizational, and financial aspects.

Additive linear multi-criteria models are the most common in both IA and MAVT applications [45, 54, 58, 84–86]. Such aggregation models require so-called *weights*. These scaling factors represent the importance of each criterion relative to the other criteria. In the absence of clear priorities, IA weights are often assumed equal, if not determined by experts or statistical decomposition [45, 85]. In MAVT, the weights are usually based on the decision-makers’ preferences, given the ranges of the alternatives under consideration (i.e. the best and worst possible outcome, see [2, 52, 55]). Additionally to weights, for each criterion a normalization function is required. These translate measurement units into commensurate, neutral units. Linear transformation is often assumed. The implications of these model assumptions are usually not carefully scrutinized in application studies, thus ignoring a large body of literature that evidences that these simplifying assumptions are often inappropriate [87–93]. This is despite the fact that manifold ways for informed, more appropriate choices are available [69, 85, 94]. If uncertainty is considered, mostly only uncertainties of performance assessment are covered. The uncertainty of a decision maker concerning her (or his) preferences is commonly overlooked [55, 95, 96].

### 1.3 Aims and structure of this paper

We follow two aims in this paper. Firstly, we aim to analyze whether and how the evaluation model assumptions and the degree to which stakeholder preferences that are represented by these models affect the outcomes. We use the example of water supply planning in a real Swiss case study. Secondly, we want to explore the performance of alternatives that are not usually considered in current water supply planning. By this, we contribute to the literature that demonstrates the conceptual and practical usefulness of using an MCDA approach—and specifically multi-attribute value theory (MAVT)–in supporting long-term water supply planning. For the case study, we followed the four steps of a value-focused MCDA procedure–employing a MAVT model–as summarized in section 2. We present and discuss the impact of models on the results by using two simpler IA and three MAVT evaluation model layouts of increasing complexity (section 3). Finally, we give recommendations for improving the current water supply infrastructure in the case study and for choosing an evaluation model in section 4. In section 5, we draw conclusions, which encompass the main insights regarding methodology as well as the performance of conventional and unconventional water supply alternatives in the specific case and beyond.

## 2 Materials and methods

We followed a structured MAVT process that is commonly organized into 4–6 interdependent steps, presented below [1, 2, 69]. This research project did not support an ongoing decision process, but was of informative interest to the stakeholders in the case study. Thus, the final discussion with stakeholders and the creation of new, better performing alternatives (step 5) as well as implementation, monitoring, and review (step 6) are not addressed. Instead, our emphasis is on the attribute outcome assessment (impact assessment) (step 2) and evaluation of alternatives (step 4). We discuss the design and facilitation of the entire decision process in separate publications (for a full publications list of the SWIP project see [97]). These include the analysis and selection of stakeholders [75], the modeling approach to predict water pipe failure and outcome of pipe rehabilitation strategies in Scholten, et al. [98], and preference elicitation is described in detail in Scholten, et al. [96]. We provide more details concerning the raw and the model input data in the supporting information (S1 File) to this article.

### 2.1 Case study

Five water utilities of four small municipalities in the upper catchment area of the Mönchaltorfer Aa river near Zurich, Switzerland participated in the case study. The area covers 24’200 inhabitants (2010) and the water supply network lengths range from 7 to 80km each. The water sources in the region are ground- and spring water (about 55% of total water demand) in addition to treated lake water, imported from a regional water supply cooperative. Water abstraction, treatment and supply infrastructures are managed by the utilities, which are either part of the municipality or local cooperatives. Planning accords to cantonal authority provisions, including a ‘General Water Supply Project/Plan (GWP)’ (updated every 10–15 years). Planning is usually supported by two engineering consultancies with long-standing operation in the region. Rehabilitation management differs by utility, ranging from purely reactive repair and replacement to proactive replacement of ca. 1% of the pipe network per year. Currently, water supply in the region is not critical. However, achieving more resilience to adverse events (e.g. droughts), addressing rehabilitation, and maintenance needs are issues of increasing concern in the region. This situation is typical for Switzerland [99], where approximately 3000 water utilities are in charge of the water supply for roughly 2600 municipalities [100]. More than half the population relies on water utilities with less than 10000 connected inhabitants [99]. Understaffing and avocational involvement of workforce are common outside the larger cities [100]. Consequentially, management and rehabilitation predominantly follow reactive strategies. High levels of fragmentation are a common issue in many OECD countries [101, 102].

### 2.2 Structure the decision problem (step 1)

In **step 1**, the decision problem is structured, including framing, setting system boundaries, stakeholder selection, defining the objectives hierarchy, and alternatives [1, 29, 69]. A thorough stakeholder and social network analysis was performed to identify those individuals or organizations that are most important for water infrastructure planning in the region, see details in [103]. We adhere to the definition of Freeman [104, p.46], according to which a stakeholder is *‘any group or individual who can affect or is affected by the achievement of the organization’s objectives’*. A set of 10 out of 29 identified stakeholders was selected for participation in this study, based on their influence and affectedness, as well as the formal and information resources they were able provide to infrastructure planning [96]. The objectives hierarchy and alternatives were defined together with the stakeholders, presented in Lienert, et al. [75]. Overall, 44 fundamental objectives relevant to achieving a ‘good water supply infrastructure’ were defined and hierarchically structured (see objectives hierarchy in supporting information Fig B.1 in S1 File). Five top-level objectives were determined: ‘high intergenerational equity’, ‘high resources and groundwater protection’, ‘good water supply’, ‘high social acceptance’, and ‘low costs’. These are concretized by lower-level objectives, e.g. for ‘high resources and groundwater protection’, the lower-level objectives are ‘high groundwater protection’ and ‘low energy consumption’. The sub-objectives for ‘good water supply’ were differentiated into quality, quantity and reliability aspects of drinking water (dw) supply, household water (hw) supply, and firefighting water (ffw) supply. Water quality was judged irrelevant for ffw supply. Altogether 30 quantifiable attributes (elsewhere also called performance indicators, criteria, or metrics) were identified to measure attainment of the objectives. The objectives hierarchy and attributes were developed and agreed with the stakeholders (for details see [75, 96]). We used the eleven water supply alternatives (also known as options or measures) as outlined in Lienert, et al. [75] (summarized in Table 1, see also Table B.3 in S1 File for details) and added two alternatives: A0 (*Current system*) representing the current technological, organizational, and managerial situation in the case study and A6*, a variant of A6 (*Maximal collaboration*, *centralized*), which allows importing more than 10% of the water from the regional water cooperative.

**Table 1:** Decision alternatives. Details in Table B.3 in S1 File. O&M: operation and maintenance.

Alternative	Description
A0 *–Current system*.	Five individual water utilities, mostly reactive O&M. Water from springs, groundwater, and a lake (purified) is centrally supplied for all water uses.
A1a / A1b *–Centralized*, *privatization*, *high environmental protection*	A1a: As A0, but managed by one regional, multi-sector private contractor. Extensive, proactive O&M. More advanced treatment of lake and groundwater. A1b: As A1a with IKA (intercommunal agency) as provider.
A2 *–Centralized IKA*, *rain stored*	As A1a, yet managed by IKA, moderate O&M efforts. Decentralized rainwater collection in tanks for firefighting.
A3 *–Fully decentralized*	High fragmentation where households contract services to third parties. Reactive, low to moderate O&M. Bottled potable water, household water from rainwater or delivered by lorry (treated in-house), decentralized rain-fed firefighting tanks.
A4 *–Decaying centralized infrastructure*, *decentralized outskirts*	Responsibilities shared between cooperatives, municipalities, and households. Minimal O&M. Centralized system within 2010 boundaries (potable quality not ensured), all else decentralized (supplied by lorries). Treatment in households.
A5 *–Decaying infrastructure everywhere*	Households contract services to third parties. Minimal O&M. Private delivery service recharges decentralized tanks with hygienically safe water (tanks are chlorinated). Firefighting-water is separate. Only spring- and groundwater.
A6 / A6*–Centralized, *maximal collaboration*	A6: Maximal collaboration across municipalities and sectors with full service provision by one cooperative. Proactive, moderate O&M. Reduced pipe diameter, rainwater for toilet flushing, decentralized firefighting tanks. Max. 10% of water imported from lake water supplier. A6*: as A6, without import restrictions.
A7 *–Mixed responsibility*, *fully decentralized with on-site treatment*	One cooperative for water services across municipalities. Proactive, moderate O&M, only decentralized assets are replaced. Water delivered by lorry with point-of-entry treatment in households and combined rainwater use.
A8a / A8b *–Status quo with storm water retention*	A8a: One integrated water and wastewater service, run jointly by the municipalities. Proactive, moderate O&M. Centralized water treatment and supply for all uses. A8b: same as A8b, but new areas dimensioned on reduced water flows.
A9 *–Centralized*, *privatization*, *minimal maintenance*	Full contracting of water infrastructures. Consumers choose their contract provider. Reactive, minimal O&M. Centralized treatment and supply, new areas dimensioned on maximum household demand, decentralized tanks for firefighting.

### 2.3 Assess performance of alternatives (step 2)

In **step 2**, the performance regarding the attributes is determined. This is done for all 13 alternatives and 4 future scenarios over a time span of 40 years. Consideration of this time span was necessary to model infrastructure development, yet only the performance at the endpoint of the time window (2050) was considered for evaluation. This applies to all attributes except *costchange*, where the mean annual cost increase over the whole time span was used.

#### 2.3.1 Predicted performance of alternatives

For each alternative and future scenario, the project team set up a cross-impact matrix that specifies which attributes are presumably impacted by a particular characteristic of the alternative and scenario (Table B.2 in S1 File). Predictions were made for each alternative and each attribute, in four future scenarios over 40 years (2010–2050). Predictions are based on the following (see also [96]):

1. **Alternative definition** (e.g. number of infrastructure sectors that collaborate in planning and construction, *collab*) or infrastructure dimensioning (e.g. areal demand for installations in households, *area*). The prediction resulted directly from dimensioning and we assumed no additional uncertainty. Tanks for example were dimensioned on the maximum capacity needed; consequently, the required space on the private property was determined by the standard tank sizes.
2. **Expert estimation,** where the interval estimates about e.g. the aesthetic and microbial drinking water quality (*aes_dw*, *faecal_dw*) or technical flexibility (*adapt*) were interpreted as 90% confidence intervals of a normal distribution. The upper value of the range was taken as 95% and the lower value as 5% quantile. The mean and standard deviation were derived from these (Table B.2 in S1 File). The experts were independent persons and not otherwise involved in this case study with the exception of one person, the representative of the Cantonal water quality laboratory (SH8). This exception was necessary because not enough qualified experts could be found for estimating water quality implications for the different alternatives in the target system. Therefore, two independent experts and SH 8 provided water quality estimates as experts. Additionally, SH 8 also participated as stakeholder in preference elicitation.
3. **Detailed models** or combinations for assessing e.g. the rehabilitation demand (*rehab)* and reliability of water supply (*reliab_dw*) [98]. Probability distributions were formulated as follows: If a specific distribution could be assumed, this distribution was used. Otherwise, an output sample was created and several possible distributions were fitted (namely: normal, lognormal, beta, gamma, logistic, and truncated normal distribution). Quantile-quantile and histogram plots were used to identify the best-fitting distribution.

Table B.1.1 in S1 File (supporting information) summarizes the 30 attributes, corresponding objectives, min-max ranges, and how the estimates were obtained.

#### 2.3.2 Future scenarios and uncertainty

Four future scenarios were used to assess the robustness of the alternatives to changing framework conditions over the considered time horizon (2010–2050). We followed the ‘Intuitive Logistics’ school [34]. Accordingly, no probabilities were assigned to scenarios, because all scenarios are understood as equally possible visions of the future. The changes encompass urbanization (rate of population increase/ decrease), the economic situation (real income development), people’s attitudes and regulations about the environment, and water demand. Three scenarios (*Boom*, *Doom* and *Quality of life*) were developed in a stakeholder workshop [75]. The fourth, *Status quo*, propagates the current situation (A.1 and A.2 in S1 File). The uncertainties of attribute predictions and preferences were encompassed and propagated by probability distributions. All attribute predictions were assumed independent such that the attribute outcomes could be independently sampled for each alternative and scenario. An attribute sample size of n = 10’000 per alternative and scenario was used.

### 2.4 Determine the decision makers’ preferences (step 3)

**Step 3** aims at specifying the preferences of decision makers or stakeholders, i.e. how attribute outcomes regarding the objectives are weighted, valued, and aggregated. Using the classical MAVT approach, these preference components are elicited separately and later aggregated to form the overall value function for each stakeholder [29, 52, 69]. Thus, for each of our ten stakeholders, 44 imprecise SWING weight intervals for aggregation at each node of the objectives hierarchy were elicited [details in 96]. We did not elicit all 30 marginal value functions over the attributes from each stakeholder, but only detailed information for those 1–4 attributes that they had stated as most important in a prior online survey. Additional rough information about curvatures was elicited for the others as described in Scholten, et al. [96], which was used to restrict the sampling range for the value function’s curvature parameter. If no value information at all could be elicited, any form is theoretically possible, and we sampled the curvature parameter over a wide range so as to encompass strongly concave, convex, and linear shapes (for modelling assumptions see 2.5.1 and part D in S1 File). Some stakeholders stated acceptance thresholds (veto levels) for particular attributes. Acceptance thresholds cause individual objectives, branches of the objectives hierarchy, or the overall value to be set to zero for an alternative, if its attribute outcomes do not satisfy minimum performance criteria.

Preferences were elicited from ten stakeholders from the local to national level. They were selected based on their degree of influence and affectedness as well as centrality in the stakeholder network [103]: Municipal (underground) engineer (SH1), operating staff (SH2), local water supply cooperative (SH3), municipal administration and finance (SH4), engineering consultant (SH5), regional water supply cooperative (SH6), cantonal environmental protection agency (SH7), cantonal water quality laboratory (SH8); Swiss gas and water industry association (SH9), and national environmental protection agency (SH10). Their preferences are summarized in the supporting information (part C, S1 File).

### 2.5 Evaluate and compare alternatives (step 4)

In **step 4**, the attribute outcomes (e.g. expected water quality, costs) and stakeholder preferences are combined into an aggregate score. This *overall value* represents the desirability of the alternative. It is used to compare and rank alternatives by means of a multi-attribute value model. We implemented and evaluated this in *R* [105], also using the contributed libraries *data*.*table* [106], *ggplot2* [107], *msm* [108], *reshape2* [109], and *utility* [110].

#### 2.5.1 Evaluation model design and main assumptions

We analyze the ranking of alternatives (on expected overall values) using five evaluation model layouts (Table 2).

**Table 2:** Five experimental evaluation model layouts L1–5.

NR	Name	Weights (*w*_*i*_), see E.1, S1 File	Attribute-to-value transformation (shape of value function)	Aggregation model	Uncertainty considered
**L1**	Integrated assessment (IA)–bottom up	All *w*_*i*_ = 1/30	Linear	Additive on lowest level, ignoring hierarchical structure of objectives	Attribute uncertainty and scenarios
**L2**	Integrated assessment (IA)–hierarchical	Equal weights on each hierarchical level, multiplied down the hierarchy	Linear	Additive on all levels	Attribute uncertainty and scenarios
**L3**	Additive-linear-MAVT, no thresholds	Mid-point of weight intervals elicited from stakeholders.	Linear value functions unless elicited in detail.	Additive on all levels	Attribute uncertainty and scenarios
**L4**	Additive-linear-MAVT with **a)** stated acceptance thresholds (AT)	Mid-point of weight intervals elicited from stakeholders.	Linear value functions unless elicited in detail.	Additive on all levels, a) stated acceptance thresholds	Attribute uncertainty and scenarios
	**b)** adjusted ATs			b) adjusted AT’s	Attribute uncertainty and scenarios
**L5**	Mixed-nonlinear-MAVT with adjusted ATs	Elicited weights including their uncertainty; 1’000 samples drawn from distribution.	Exponential value function parameter fitted to preferences from detailed elicitation, otherwise sampled from roughly assessed form. Further assumptions see S-D.1.	Mixture aggregation; mixture parameter α_k_ sampled on hierarchical levels k; adjusted ATs.	Attribute and preference uncertainty and scenarios.

MAVT = Multi-Attribute Value Theory.

Layout 1 and 2 (L1, L2) are IA models, i.e. do not consider any stakeholder weights, value functions, or aggregation preferences. They assume equal weights for all objectives and linear transformation of marginal value functions to a dimensionless value x. The difference between L1 and L2 is aggregation. In L1 we aggregate on the lowest level (weighted arithmetic mean), whereas in L2 aggregation is according to the objectives hierarchy in Fig B.1 in S1 File (hierarchically weighted arithmetic mean). An overview of the weights used in L1–L5 is given in E.1. Implications of hierarchical weighting are discussed in section 4.2. Attribute prediction uncertainty (B.4 in S1 File) is propagated in L1–L2. The aggregation model to obtain the overall value V(A) ϵ [0,1] (as a measure of desirability) of each alternative A is additive (e.g. [29]):

$$\begin{array}{cc} V_{add}\left(A\right)=\sum_{i=1}^{n}w_{i}\cdot v_{i}(x_{i}\left(A\right)); & \sum_{i=1}^{n}w_{i}=1 \end{array}$$ (1)

The weights *w*_*i*_ measure the importance of objective *i* relative to the other objectives given the range of possible attribute levels *x*_*i*_. Equal weights imply that all criteria (objectives) are equally important. Marginal value functions *v*_*i*_ describe how well objective *i* is fulfilled by achieving attribute levels *x*_*i*_, i.e. they convert attribute outcome levels to dimensionless values between 0 (worst) and 1 (best). The attribute levels are usually linearly transformed to the neutral 0–1 scale, although exponential and power functions, s-shape curves, step-functions, and others have been suggested (e.g. [29, 45, 111]). Linear transformation means that equally distanced improvements on the attribute scale lead to equally distanced improvements on the value scale.

Layouts L3–L5 (Table 2) include stakeholder preferences of increasing complexity. In L3, the parameters of the aggregation model in Eq 1 were defined as typical for simplified MAVT, using means of the elicited weight intervals and the best fitting exponential value function parameters. If no value functions were elicited in detail, linear transformation was used.

In L4a, individual stakeholder acceptance thresholds (ATs) for selected objectives were additionally included. Some ATs contradict the current situation in the sense that the current performance—generally perceived as satisfactory by the interviewed stakeholders—may be worse than the ATs defined by the same stakeholder (see Discussion). We therefore adjusted these in L4b so that alternatives performing as well as the current (A0) were not eliminated.

In L5, full preferences including attribute and preference uncertainty, a non-additive aggregation model (Eq 2), and adjusted acceptance thresholds were used (Table 2). We deem a non-additive model necessary because in water supply planning, good performance on one objective (e.g. low costs) cannot fully compensate poor performance on another (e.g. potable water quality), i.e. there is no full compensation between objectives. Therefore we used a mixed model of an additive [69] and a Cobb-Douglas model [112], essentially a mix of the weighted arithmetic and geometric mean [111]. This model disadvantages alternatives with extreme outcomes over alternatives with more equilibrated outcomes on individual objectives:

$$V\left(A\right)=\alpha \cdot V_{add}\left(A\right)+\left(1-\alpha \right)\cdot V_{CD}\left(A\right)=\alpha \sum_{i=1}^{n}w_{i}\cdot v_{i}\left(x_{i}\left(A\right)\right)+\left(1-\alpha \right)\prod_{i=1}^{n}v_{i}{\left(x_{i}\left(A\right)\right)}^{w_{i}}$$ (2)

α ϵ [0,1] determines how strongly additive or non-additive the model is. With decreasing α, the compensation between extreme criteria decreases.

For each stakeholder, 1’000 combinations of weight, value function, and aggregation parameters α were sampled and aggregated according to this model. As α was not elicited, we sampled over the full range of possible values (0 to 1), see detailed preference modeling assumptions in D.1 in S1 File. To compare outcomes of the five evaluation layouts, we also measured the distinguishability (value range) between the best and worst alternative, the improvement potential over the current system (A0) if the best alternative is selected, and the Kendall rank correlation coefficient τ [113] of the alternative ranking compared to the ranking in L1 as a measure of rank stability. Since in L3–L5 outcomes are stated for individual stakeholders, the summarized results are averages of the individual stakeholder results.

#### 2.5.2 Dominated alternatives

To avoid unnecessary analysis, alternatives can be excluded if they are dominated. An alternative A dominates another alternative B, if A outperforms B on at least one attribute and performs equally on all others (e.g. [29]). Dominance can also be determined on the overall value instead of attribute level. Dominated alternatives are removed because any approximately rational decision maker would prefer the dominating alternative to the dominated one.

In case of uncertain attribute (value) outcomes with overlapping probability distributions, stochastic dominance concepts are used [114–116]. First order stochastic dominance (FSD) allows determining whether A dominates B. This is the case if the risk profiles 1—P(x) of the attributes’ cumulative probability functions P(x) of A are always above those of B. The risk profiles represent the cumulative probability of an alternative to achieve or exceed a certain outcome. With 13 alternatives and 30 attributes, the effort to do so on attribute level would probably exceed that of calculating the overall values of the alternatives. We thus refrained from performing pairwise comparisons on the attribute level (but still detected two obvious dominance relationships) and instead calculated the risk profiles on the overall value of alternatives for individual stakeholders where otherwise no conclusions would have been possible.

### 2.6 Ethics statement

The research presented builds on expertise from 18 subject matter experts. Eleven of these are scientists who actively publish in the respective fields. The other seven are practitioners whose expertise stems from everyday practice concerning the topics inquired. All experts were informed beforehand by phone or in written form about the aims of the study and the expertise requested, before they would agree to participate. Unless shared per e-mail, their expertise was consulted in semi-structured interviews during which it was agreed how the experts may be cited. For long interviews, a summary of the interview was provided to the experts for review. For short interviews, the main points were summarized at the end of the interview so the expert could make changes if desired. All experts were fully aware of, and agreed with, the fact that the information provided will be used for evaluating different water supply alternatives and that we aimed for scientific publication. As this type of expert interviews is not subjected to ethical restrictions, we did not ask for explicit written consent in addition to the oral consent expressed during the interviews.

In addition, the preferences of ten individual stakeholders were used for MCDA modeling as reported in Scholten, et al. [96]. All stakeholders were informed beforehand about the purpose of the study, the decision problem, objectives, and attributes considered, as well as the contents of the online survey and interview. In the interview, individual agreements were made on how we may cite their inputs in publications resulting from this study. After the interview, they received a written summary of the interview for review, including a statement that we assume their consent with the content and citation unless they inform us otherwise within four weeks. The summaries were corrected according to the received written feedback. All stakeholders agreed to be cited by their function and organization. We do not reveal their names to protect their anonymity. Furthermore, we held information meetings and obtained written consent to perform research within their jurisdiction by the elected political representatives of the case study municipalities. We did not seek prior approval or review by the ethics committee of our research organization, as this type of interviews is not subjected to ethical restrictions.

## 3 Results

### 3.1 Ranking of alternatives based on different evaluation model layouts

#### 3.1.1 Integrated assessment (L1–2)

The first evaluation model layouts are based on classical integrated assessment (IA), which does not take any preferences from stakeholders into account. In the evaluation model layout L1 (bottom-up aggregation; each attribute achieves the same weight *w*_*i*_ = 1/30; Table 2), alternative A1b would be the best one in the Status quo scenario (Table 3), achieving the first rank and an expected value of EV(A1b) = 0.788. In the Boom scenario (columns without shading), alternative A6 is ranked first (0.776). In the evaluation model layout L2 (hierarchical aggregation; Table 3), however, A6 and A6* are best in the Status quo (0.802) and Boom (0.752) scenarios. The current alternative A0 is always outperformed by other alternatives, ranking 6^th^ in L1 (Status quo: 0.732, Boom: 0.729), 6^th^ in L2-Status quo (0.717), and 7^th^ in L2-Boom (0.704). Furthermore, A1b, A2, A6, and A6* constantly rank higher than A0. A8b also ranks higher than A0 except in L2-Status quo. In contrast, alternative A5 is always worst (11^th^ rank, expected value 0.616–0.658). Note that selecting one of the best alternatives instead of A0 would lead to only small improvements in value ranging from 0.047 (L1, Boom scenario, Table 3) to 0.085 (L2, Status quo). Assuming that decision-makers aim to maximize the value across scenarios and that both scenarios are equally important, the alternative with the highest average value in L1 is A1b (average value = 0.775); in L2 it is A6* (0.774; Table 3).

**Table 3:** Overall value and ranking of alternatives using two integrated assessment models (L1 and L2) without stakeholder preferences.

	L1 –Bottom up aggregation (equal weights)							L2 –Hierarchical aggregation						
	Boom			Status quo				Boom			Status quo			
	R	μ	σ	R	μ	σ	Average(μ)	R	μ	σ	R	μ	σ	Average(μ)
**A1b**	2	0.763	0.025	**1**	**0.788**	**0.025**	0.775	2	0.742	0.018	3	0.796	0.020	0.769
**A2**	2	0.763	0.025	2	0.775	0.024	0.769	5	0.726	0.017	4	0.770	0.017	0.748
**A3**	9	0.683	0.025	10	0.672	0.025	0.677	8	0.690	0.014	7	0.709	0.010	0.700
**A4**	10	0.671	0.025	9	0.695	0.025	0.683	10	0.683	0.011	9	0.697	0.011	0.690
**A5**	*11*	*0*.*658*	*0*.*027*	*11*	*0*.*647*	*0*.*026*	0.653	*11*	*0*.*616*	*0*.*018*	*11*	*0*.*650*	*0*.*012*	0.633
**A6**	**1**	**0.776**	**0.025**	3	0.774	0.025	**0.775**	4	0.730	0.018	**1**	**0.802**	**0.015**	0.766
A6*	4	0.756	0.025	4	0.769	0.025	0.762	**1**	**0.752**	**0.015**	2	0.797	0.015	**0.774**
**A7**	8	0.721	0.026	7	0.719	0.026	0.720	3	0.737	0.012	5	0.738	0.012	0.737
**A8b**	5	0.740	0.025	5	0.737	0.024	0.739	6	0.709	0.014	8	0.704	0.013	0.707
**A9**	7	0.722	0.025	8	0.715	0.025	0.718	9	0.684	0.015	10	0.678	0.015	0.681
**A0**	6	0.729	0.025	6	0.732	0.025	0.731	7	0.704	0.014	6	0.717	0.013	0.711

A1b–A0 are 11 water supply alternatives (Table B.3 in S1 File), and their expected values (mean μ), standard deviations (σ) and ranks (R) for two scenarios. For assumptions underlying L1 and L2 see section 2.5.1. Bold = alternative achieved the highest value and best rank, italic = alternative achieved the lowest value and worst rank.

Furthermore, the difference between the average value of the highest and lowest-ranking alternative in L1 is only 0.14 (Status quo)–0.118 (Boom) and in L2 0.152–0.136 respectively (Table 4). Thus, the high uncertainty of individual attribute outcomes (wide attribute probability distributions) is not reflected in a large uncertainty of the alternatives’ overall mean value.

**Table 4:** Distinguishability, improvement potential, and rank stability of the alternatives in five evaluation model layouts (L1–5).

	Boom					Status quo				
	L1	L2	L3	L4b	L5	L1	L2	L3	L4b	L5
**Distinguishability (value range, V**_**max**_**-V**_**min**_**)**	0.118	0.136	0.242	0.352	0.415	0.140	0.152	0.184	0.267	0.363
**Value improvement potential** **(V**_**max**_**-V**_**A0**_**)**	0.047	0.047	0.046	0.053	0.072	0.055	0.085	0.080	0.085	0.094
**Rank stability (Kendall-τ)**	1.000	0.600	0.564	0.564	0.600	1.000	0.600	0.709	0.636	0.600

Assumptions see section 2.5.1. The value range and improvement potential of the current system (A0) compared to the best alternative are calculated on the expected values of individual alternatives. In L3–L5, outcomes for individual stakeholders are averaged over all stakeholders to an average value for each alternative, which is used for ranking. Kendall-τ is the rank correlation coefficient of the rankings of alternatives in L2–L5 compared to L1. In L3–5, the expected values are averaged across stakeholders before ranking.

#### 3.1.2 Results of MCDA evaluation layouts (L3–5)

**Fig 1:**
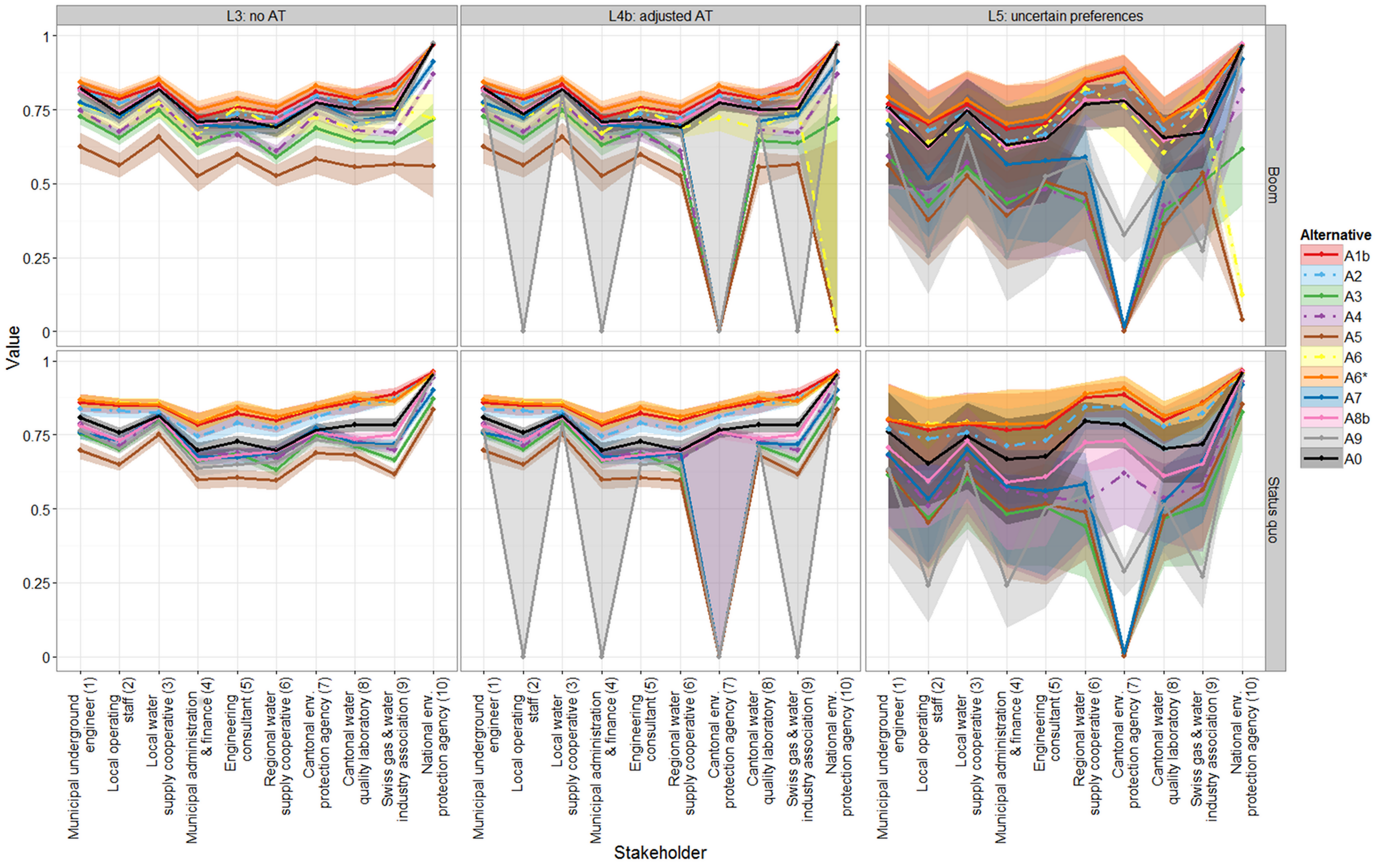
Overall value of alternatives under different preference assumptions for ten stakeholders and two future scenarios using evaluation model layouts L3, L4b, and L5 (cf. Table 2). Lines represent the median (50% quantile), uncertainty bands in corresponding colors the 5–95% quantiles. Alternative A0 (black solid line): current water supply system. The uncertainty in L3 and L4 is entirely due to the uncertainty of the attribute predictions; in L5 additionally also due to uncertain preferences of stakeholders. AT: Acceptance Thresholds.

The overall values for the evaluation layouts L3 to L5, which include preference information from individual stakeholders, are displayed in Fig 1 and in Table E.2 in S1 File. The aggregated mean value, standard deviation, and value rank of the alternatives across stakeholders are also shown in Table E.2 in S1 File. Layout L3 uses several simplifying assumptions (no acceptance thresholds, additive aggregation, linear value functions unless stated otherwise, sure preferences; Table 2), with the result that the value profiles and uncertainty ranges of the alternatives (colored lines, shading in respective color) rarely cross. In other words, there is little divergence in the relative performance of alternatives across stakeholders despite the fact that the different stakeholders assigned considerably different weights to the objectives (Table C.1 in S1 File). Across both scenarios and stakeholders, the alternatives A1b (red line in Fig 1), A2 (light blue), and A6* (orange) receive the highest mean values. Aggregating the expected values across stakeholders, the average values for the best-ranked alternatives A1b, A2, and A6* amount to 0.851, 0.830, 0.856, respectively, in the Status quo scenario, and 0.806, 0.792, 0.818 in the Boom scenario (see “aggregated SH1-10”, last column in Table E.2 in S1 File). These alternatives A1b, A2, and A6* clearly outperform the current system A0 (black line in Fig 1 average expected value across stakeholders in Status quo: 0.779, Boom: 0.767; Table E.2 in S1 File). The performance of alternative A6 differs by scenario. It is among the best in the Status quo (averaged expected value: 0.855, ranks 1–3; Table E.2 in S1 File), but worse than A0 for most stakeholders in the Boom scenario (0.726, ranks 3–9). The remaining alternatives either overlap with A0 or achieve lower values. The worst performing alternative in both scenarios is A5 for all stakeholders (Fig 1, brown line) and both scenarios (average value across stakeholders in Status quo: 0.672, Boom: 0.575; Table E.2 in S1 File), followed by A3 (Status quo: 0.722, Boom: 0.672; green). Looking at individual stakeholders, A6* would be best for half of the stakeholders (SH1, SH5–8) in the Status quo scenario, but for SH2–4 alternative A6 would have the highest value, and A1b is best for SH9–10 (Table E.2 in S1 File). In the Boom scenario, A6* is best for SH1–8, but A1b for SH9 and A2 for SH10. As in L1–2, the improvement in value achieved by selecting the best alternative instead of A0 ranges from 0.08 (Status quo) to 0.046 (Boom) on average. The difference in value between the best and worst alternative is 0.184–0.242 value units (Table 4).

Some stakeholders stated acceptance thresholds (AT) for specific attributes. An AT renders an alternative unacceptable (i.e. it receives an aggregated value of 0) if the respective attribute does not meet the stated AT. The originally stated AT were implemented in evaluation model layout L4a. Hereby, all alternatives except A6*–including the current system A0 –would be discarded due to the possibility of not complying with the thresholds of some stakeholders (see Fig E.2 in S1 File). If instead thresholds are adjusted so that the performance of the current system is considered acceptable (evaluation model layout L4b, middle panel in Fig 1), only alternatives A3, A5, A7, and A9 are discarded for one or more stakeholders, and A4 and A6 in the Boom scenario. The impact of the acceptance thresholds on these alternatives is reflected in lower values compared to L3. The value profiles and rankings of the remaining alternatives are identical to L3. Alternatives A1b, A2, and A6* still achieve the highest overall value across stakeholders. The difference in value between the highest and lowest ranked alternative in L4b is larger than in L1–3 with 0.267 (Status quo)–0.352 (Boom) value units (Table 4).

**Fig 2:**
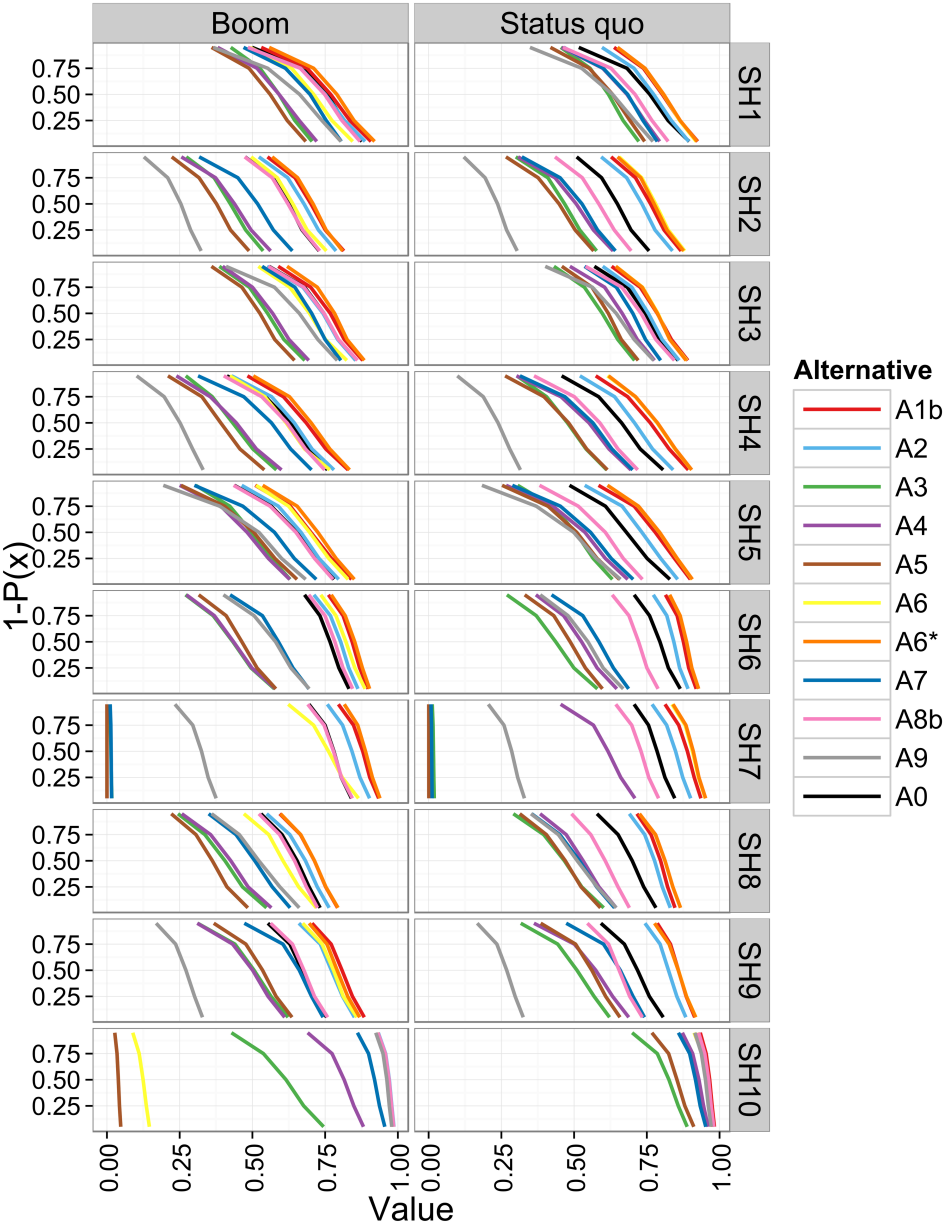
Risk profiles of the expected value of the alternatives for evaluation layout L5 by individual stakeholders (SH1–10) under two scenarios. P(x): cumulative probability. First order stochastic dominance holds if the curve of one alternative is to the right of another.

In evaluation model layout L5, the uncertainty bands are considerably wider and overlap more than in L3 and L4b (Fig 1, right panel). The uncertainty encompasses not only attribute uncertainties, but also the uncertainty of the preferences of stakeholders (weights assigned to objectives, shape of marginal value functions, and aggregation function). The alternatives’ average values across stakeholders span up to 0.415 value units in the Boom scenario, i.e. even more than in layout L4b (Table 4). This makes visual discrimination of the alternatives difficult (Fig 1). Therefore, we plotted the alternatives’ risk profiles by stakeholder to assess dominance regarding the overall value (Fig 2). Alternative A6* (orange lines, far right in Fig 2) is still the best alternative for SH1–8 and dominates all others in the Boom scenario (left column), with alternatives A1b (red lines), A2 (light blue), or A6 (yellow) following closely. For SH9, A1b performs best, followed by A6*, A6, and A2. For SH10, the best alternatives are A2, A6*, and A8b and nearly indistinguishable as their risk profiles overlap. In the Status quo scenario, A6* dominates all other alternatives for seven stakeholders: SH1, 2, 4–8. For the remaining SH3, SH9, and SH10, alternatives A6* and A1b are best, but their risk profiles cross, so that none dominates. As in the other evaluation layouts, the current water supply system A0 (black line) is dominated by several alternatives for all stakeholders and scenarios (e.g. A1b, A2, A6*). Averaging across stakeholders, alternative A6* has the highest cross-stakeholder mean value (Boom: 0.784, Status quo: 0.831, Table E.2 in S1 File). The average improvement of the expected value of selecting the best alternative over A0 lies between 0.094 (Status quo) and 0.072 (Boom) value units (Table 4).

### 3.2 Performance of alternatives

**Fig 3:**
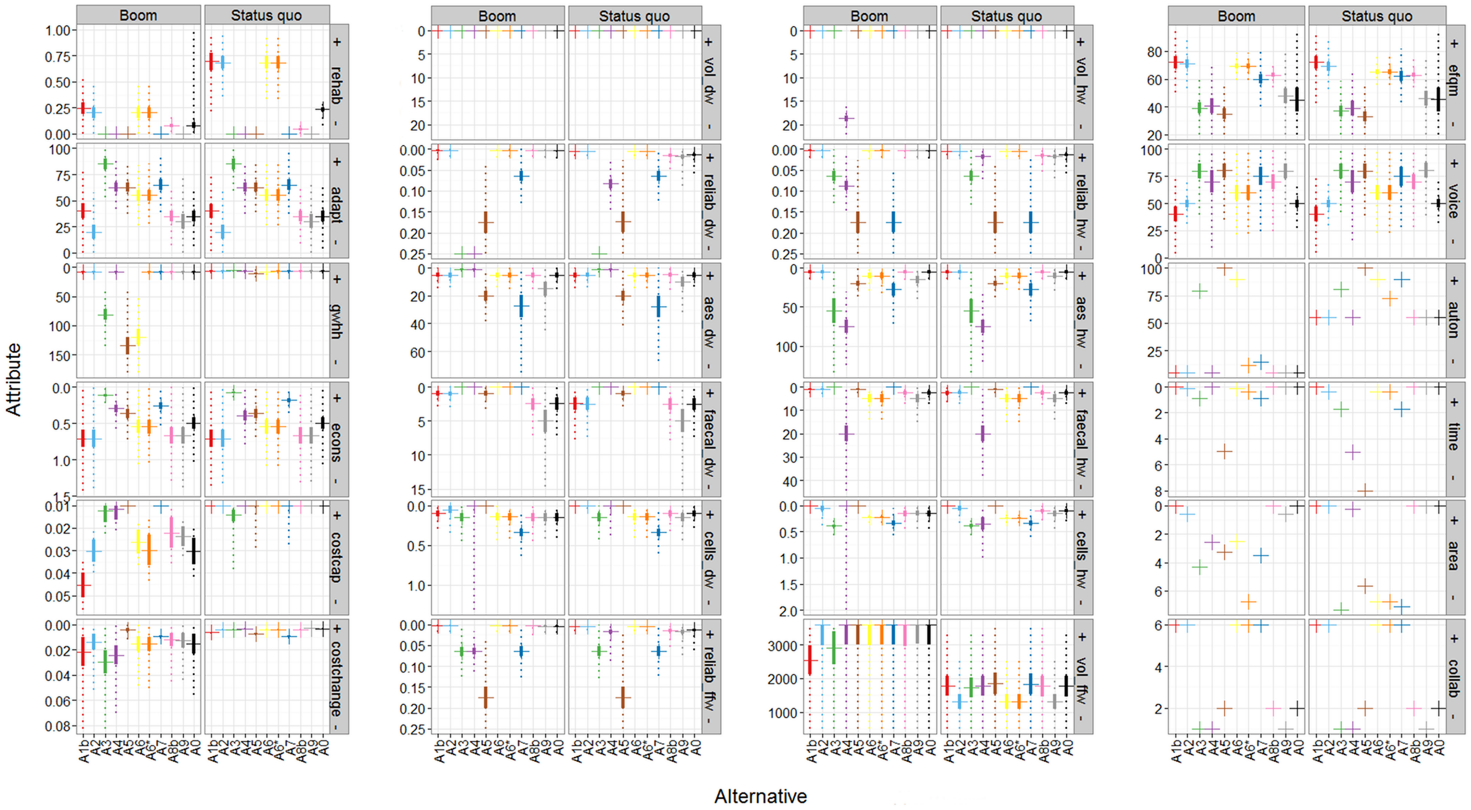
Outcomes for 24 attributes (in boxes) for the 11 non-dominated alternatives (A1b–A0) in the Boom and Status quo scenarios. Horizontal lines (crosses) mark the median (50% quantile), solid, vertical lines the interquartile ranges (25–75% quantiles) and dotted, vertical lines the 5–95% quantiles. The direction of improvement is indicated by + and—signs on the grey labels carrying the abbreviated attributes (explained in Table B.1 in S1 File). Dominated alternatives (A1a, A8a) and attributes without detailed predictions (no3_dw, no3_hw, pest_dw, pest_hw, bta_dw, bta_hw) are not shown. Distributional assumptions are given in Table B.4 in S1 File.

Fig 3 shows the predicted attribute outcomes (also termed “impact” in IA) for all non-dominated alternatives in the Boom (BO) and Status quo (SQ) scenarios. Results for the Doom and Quality of life scenarios are not discussed, because the performance in the Doom scenario is very similar to the Status quo scenario. This similarity was not intended, but resulted from the way we generated the scenarios (discussed in section 4.3). Results for the Quality of life scenario lie between the SQ and BO scenario (Fig E.1 in S1 File). Alternatives A1a and A8a were removed as they are dominated on the attribute level: A1a is dominated by A1b because it performs poorer than A1b on the flexibility to technological expansion and deconstruction (*adapt*) and degree of codetermination *(voice)*. All other attributes are the same (cf. Table B.4 in S1 File). Moreover, A8a is identical to A8b on all attributes, but has a lower median annual cost increase (*costchange*) and annual costs (*costcap*) with smaller uncertainty bands. First order stochastic dominance is fulfilled as P[A8b ≤ x] ≥ P[A8a ≤ x].

The attribute outcomes displayed in Fig 3 differ either 1) across alternatives and future scenarios, or 2) across alternatives, but not (significantly) across scenarios, or 3) not at all (identical for all alternatives/ scenarios).

1. Altogether 14 of the 30 attributes are of the first type, and all five highest level objectives are affected by having at least one attribute with different outcome levels for different alternatives as well as future scenarios: **1) high intergenerational equity:** (realization of the rehabilitation demand, *rehab*), **2) high resources and groundwater protection** (utilization of groundwater recharge, *gwhh)*, **3) good water supply** (reliability of drinking, household, and firefighting water supply, *reliab_dw*, *reliab_hw*, *reliab_ffw*; changes in total cell counts of drinking and household water, *cells_dw*, *cells_hw*; household water and firefighting water quantity, *vol_hw*, *vol_ffw*), **4) high social acceptance** (resources autonomy, *auton*; necessary end-user time investment, *time*; additional area demand on private property, *area*), **5) Low costs** (annual costs per inhabitant, *costcap*; mean annual cost increase, *costchange*).
2. Nine attributes differ in their outcome levels for individual alternatives, but have (nearly) identical outcome levels across scenarios. All highest-level objectives except costs are affected: **1) high intergenerational equity** (flexibility of technical extension or deconstruction, *adapt*), **2) high resources and groundwater protection** (energy consumption for treatment and transport, *econs*), **3) good water supply** (esthetic drinking and household water quality, *aes_dw*, *aes_hw*; hygienic drinking and household water concerns, *faecal_dw*, *faecal_hw*), **4) high social acceptance** (quality of operations and management, *efqm*; codetermination, *voice;*number of collaborating infrastructure sectors, *collab*).
3. Finally, seven attributes achieve identical attribute outcomes for all alternatives and scenarios. These belong only to the objective **good water supply**: drinking water quantity (*vol_dw;* Fig 3) and all physico-chemical quality parameters for drinking and household water supply (*no3_dw/hw*, *pest_dw/hw*, *bta_dw/hw*, not shown, see Table B.4 in S1 File).

The reasons for the differences in the predicted attribute outcomes between alternatives can be explained by the influence of particular alternative characteristics and underlying assumptions (Table B.2 in S1 File). The rehabilitation strategy affects 13 attributes, including intergenerational equity (*rehab*, *adapt*), reliability and quality of supply (*reliab_dw/_hw/_ffw*, *aes_dw/aes_hw*, *faecal_dw/_hw*, *cells_dw/_hw*), and costs (*costcap*, *costchange*). For all attributes except costs, more rehabilitation leads to an improvement on the attribute outcome. The choice of the technology for water treatment, storage, and distribution affect even more attributes than the rehabilitation strategy (altogether 20: *adapt*, *gwhh*, *econs*, *auton*, *time*, *area*, *costcap*, *costchange*, *vol_dw/_hw/_ffw*, *reliab_dw/_hw/_ffw*, *aes_dw/hw*, *faecal_dw/_hw*, *cells_dw/_hw*). In this case, the choice of a particular technology can affect some of these attributes positively and others negatively, depending on the particular alternative characteristics. Whether a specific characteristic presumably affects one or several of the attributes is specified in Table B.2 in S1 File. Characteristics regarding the organizational form and responsibilities, the spatial and sectoral coordination, and the funding strategy only affect four attributes (*adapt*, *collab*, *efqm*, *voice*). This may seem insignificant compared to rehabilitation and technology choices. Nonetheless, *collab*, *efqm*, and *voice* are not affected by any other assumptions and thus may become decisive. Finally, scenario assumptions reinforce the impact of the alternatives. For instance, strong population growth as assumed in the Boom scenario affects six attributes directly (*gwhh*, *auton*, *time*, *area*, *costcap*, *costchange*). This is caused by the increased water demand (due to strong population growth) in combination with the water source and supply technology, as specified in the individual alternatives. Twelve attributes are indirectly affected by the Boom scenario. Hereby, network expansion and densification are a consequence of population growth. These network characteristics can change the age and material distribution of the expanding pipe network and speed-up the transition from the current centralized system to a mixed centralized-decentralized technology if specified by the alternative (*rehab*, *reliab_dw/_hw/_ffw*, *cells_dw/_hw*, *faecal_dw/_hw*, *vol_hw/_ffw*, *econs*).

## 4 Discussion

### 4.1 Impact of evaluation model layouts L1–L5

Despite the differences of the five evaluation model layouts, the same group of alternatives ranked consistently best or worst across layouts, stakeholders, and scenarios, albeit in slightly different rank order (Table E.2 in S1 File, Fig 1; **best**: A1b, A2, A6*; **worst**: A5, A3, A9). Also, rankings of the middle alternatives, e.g. A7 and A8b, vary across models. The relatively small difference in ranking of the top alternatives can be explained by a combination of attribute predictions and preference model assumptions: In layout L1, the 30 attributes are equally weighted and additively aggregated at the same level. Layout L1 is commonly used in integrated assessment [45]. Good outcomes on some attributes can fully compensate poor outcomes on others [111]. Moreover, the more attributes there are, the less their individual influence (1/30th each in case of 30 attributes) This is reflected in the small overall distinguishability (value range) of the alternatives and little improvement if selecting the best alternative instead of alternative A0 (*Current system*; see Table 4). Hence, the ability of L1 to discriminate between alternatives is limited. This discriminative ability is slightly higher in L2 and increases with evaluation layout, indicated by an increasing spread of the overall value across alternatives (Table 4). In hierarchical weighting, the impact of individual attributes depends on their position within the objectives hierarchy and on the weighting on higher levels, as weights are split going downwards in the hierarchy. With equal weights at each level, the weight at the top-level is distributed over the lower-lying levels. Here, three of five top-level objectives have only two underlying attributes each (Fig B.1 in S1 File). Consequentially, 60% of the weight at the top-level (= 1/5*3) are equally distributed across six attributes (*rehab*, *adapt*, *gwhh*, *econs*, *costcap*, *costchange*. Each holds 10% of the overall weight. Another extreme is the weight assigned to the attribute measuring benzotriazole concentration in potable water *bta_dw*, an indicator for micropollutants (Table B.1 in S1 File). With hierarchical weighting, its weight is 0.0025 or 0.25%, i.e. almost irrelevant. [Calculation: The top-level objective *good water supply* contains 18 attributes; the sub-objective *good drinking water* receives 1/5 and then 1/3 (1/5*1/3 = 0.067), which is again split into three subobjectives at three lower lying levels (each 1/3). The weight of *bta_dw* is: (1/5*(1/3)^4^ = 0.0025.] Thus, alternatives with good performance on these attributes receive higher overall values. A6* outperforms most other alternatives on intergenerational equity (*rehab*, *adapt*) and resources and groundwater protection (*gwhh*, *econs*) (Figs 1 and 3). Although the cost attributes c*ostcap and costchange* each hold 10% of the overall weight, the differences in the attribute outcomes regarding costs are small compared to the other attributes (Fig 3). Hence, they are less influential, which explains the higher rank of A6* in layout L2 than in L1 (Fig 1).

In evaluation model layouts L3–L5, the preferences of individual stakeholders are increasingly included (Table 2). Despite different preferences, there is only a small difference in the ranking of alternatives for individual stakeholders (Fig 1, Table E.2 in S1 File). This is because a few alternatives perform well on attributes that matter to all stakeholders and that are positively affected by proactive rehabilitation management (namely: *good water supply*, *high intergenerational equity*, *high resources and groundwater protection*, cf. Table C.1 in S1 File for weights). Combined with a relatively low weight attributed to costs by most stakeholders, it is no surprise that the best alternatives A1b, A2, A6, and A6* foresee at least average rehabilitation efforts. The current system (A0) and remaining alternatives have minimal to no proactive rehabilitation. Although they sometimes outperform the best alternatives on other objectives, these are not important enough to make up for the difference (Figs E.3-12 in S1 File). As neither the mean weighting of objectives, nor the attribute predictions change in L3–L5, differences in alternative ranking are due to the complexity of the model in terms of acceptance thresholds, preference uncertainty, and aggregation form. This increasing complexity is reflected in the increasing range in overall value from the best to the worst performing alternatives, also in comparison to L1–L2 (Table 4). In other words, the difference in total value of the worst-performing alternative compared to the best-performing alternative increases with model complexity from L1–L5. Moreover, the differences between the expected values of the four best alternatives might seem insignificant (0.02 and 0.022–0.032 in L1 and L2, respectively). Yet, this is not the case, because the overall value range is also (0.118–0.14 in L1 and 0.136–0.152 in L2; Table 4), covering roughly 14% to 21% of the range.

The acceptance thresholds (AT) would lead to the removal of most alternatives in layout L4a (Fig E.2 in S1 File), including the current system (alternative A0). This is because it is may fail to achieve the requested levels of drinking water hygiene, even though none of the stakeholders mentioned the current situation as unsatisfactory. A possible explanation is over-stringent or misinterpretation of regulatory requirements. Swiss legislation requires the absence of fecal indicator bacteria in water samples taken at different times and frequencies, depending on the size of the water utility (typically 1–4 times per year in our case study) and does not allow any uncertainty. In practice, fecal indicators may still occur in spring water on days when no sampling takes place. The water quality expert assessed that currently up to five days a year with hygienic impairment are possible, e.g. after strong rain events causing surface runoff from agricultural areas which can temporarily contaminate springs. Because we assessed attribute outcomes after preference elicitation, we could not address during the interviews that the some of the stated AT are stricter than the current state. To avoid potential misunderstandings in future studies, attribute outcomes need to be assessed first so that such discrepancies can be discussed with stakeholders. In our case, obviously the acceptance thresholds did not express what is actually practiced by the stakeholders.

### 4.2 Choosing an evaluation model

Besides the lower ability of L1 and L2 to discriminate between alternatives (section 4.2), there are also conceptual reasons for preferring MCDA to IA models. As discussed in Singh, et al. [45], indicator-based assessment is *“subject to subjectivity despite [a] lot of objectivity used in assessing”*, particularly regarding weighting, correlation, and compensability between indicators. Ultimately, the decision-makers bear responsibility for their decisions and complex decisions involve values [2]. Hence, evaluation must integrate local performance and local values, i.e. represent the decision maker’s preferences about indicators relevant to them. Also, performance assessment of any attribute outcome involves judgement [4]. The challenge lies in selecting a suitable deliberative process in which contingent issues are identified and discussed, combining both evidence-based assessments and stakeholder values as integral parts of the evaluation of alternatives. The combination of structured decision-making, supported by MCDA-type models together with other problem structuring and analytical tools such as scenario planning and uncertainty/ sensitivity analysis has proven to be a very powerful, conceptually satisfying framework to facilitate better decision-making under complexity [1, 2, 30, 31]. Regarding the selection of an appropriate evaluation model, an important consideration is the intended use of the model output. If an authoritative ranking shall be produced that *suggests* which option to choose, then more complex models are needed to correctly represent the decision-maker’s preferences. If, however, the aim is to provide a model for analysis and discussion of key trade-offs within a broader, structured decision-making effort that *supports* the decision [118, 119] (which still needs to be taken by the decision makers), then simpler models may suffice.

In our case, of the five evaluation models considered, layout L5 allows most flexibility to accommodate decision-maker preferences, including uncertainty about these preferences. The main shortcomings of layout L5 are that it is rather complex and requires large efforts for simulation and interpretation of the results. Also, individual risk attitudes are not represented in L5 as opposed to, e.g. a multi-attribute utility model [69, 120], or other models suggested to address preferences about risk [29, 121, 122]. Given that the evaluation model layouts L3–L5 led to the same alternatives being identified as either best or worst—albeit with different ranking depending on the model—one might infer that the simplest model layout L3 is sufficient. L3 assumes additive aggregation, linear marginal value functions for most attributes, and ignores acceptance thresholds. It includes stakeholder preference information concerning the weights and the uncertainty of attribute predictions. Nevertheless, the increasing differences in the rankings of alternatives with increasing complexity and uncertainty of the preference model (indicated by the decreasing Kendall-τ correlation coefficient, Table 4) caution us to generalize this conclusion. In our case, the changes in ranking are not very significant because only the middle-ranking alternatives are affected. This may be different in other cases. Many studies have found that common simplifying assumptions concerning the evaluation model do not adequately reflect the decision maker’s preferences (e.g. [87, 88–92]). The most appropriate model in a certain situation will depend not only the quality of the model itself but its suitability with the situation and skills of the decision makers and analyst [63, 65]. Therefore, the sensitivity of the results to the model structure and possible simplifications needs to be tested in each application where the result is used to produce an authoritative ranking of alternatives. When the aim is to base discussion on an analytic model, then a simpler model such as L3 may suffice.

### 4.3 Predicting the performance of alternatives

We found that attributes whose performances are driven by both the alternatives and scenarios are most useful to discriminate between alternatives, but also to assess robustness under different future scenarios. Attributes that do not differ across alternatives or scenarios do not contribute to the discrimination between alternatives and could be removed. Here, these seven attributes all belong to the objective “good water supply” (e.g. *vol_dw* in Fig 3; see Section 3.1). We kept them as the uncertainty of their performance still contributes to the uncertainty of the overall value of the alternatives. This results in a more uncertain overall value of the alternatives (Fig 1).

The future scenarios were very useful to assess robustness to unpredictable changes in future framework conditions. Particularly the strong population growth in the Boom scenario affects the performance of alternatives (Figs 1 and 3). Other important factors affecting performance were higher water use, average age of the system, and a faster technology transition for alternatives that foresee decentralization. Population increase combined with water import restrictions causes overexploitation of groundwater resources (*gwhh*). This explains the poor performance of alternative A6 in the Boom scenario compared to high performance in the Status quo (Fig 1). Alternative A6* retains its very high performance also in the Boom scenario due to lifting water import restrictions at the expense of slightly higher costs and lower resource autonomy. Since both costs and resource autonomy have only very low weights compared to groundwater protection (valued highly by all stakeholders; see Table C.1 in S1 File), this only slightly reduces the overall value of A6* in the Boom scenario compared to the Status quo. Scenario effects are also visible in alternatives A1b, A2, A6 and A6* as these perform better on the objectives *intergenerational equity* and s*ocial acceptance* in the Status quo scenario compared to Boom. This is explained by the impact of the scenario on the realization of the rehabilitation demand (*rehab*) and resource autonomy (*auton*).

As apparent from the above, we mostly learnt from comparing the two most diverging scenarios Boom and Doom. The stakeholder-driven scenario development (see [75]) created only very small differences between Doom and Status quo, and an intermediate Quality of life scenario between Status quo and Boom. This was a consequence of how the scenarios were developed with the stakeholders; i.e. that four groups elaborated one future scenario each according to their liking, without considering the work of the other groups. In hindsight, this was unfortunate, because the attribute outcomes in the Doom and Quality of life scenarios were very similar to, or in between, the other scenarios. They demanded significant efforts for evaluation without providing significant additional insights. In future studies, we recommend that more attention be placed on creating sufficiently different scenarios. This difference should encompass (i) the magnitude of impact that individual scenario drivers have on the performance of alternatives regarding the attributes, (ii) the direction of influence (positive or negative) of these drivers, and (iii) the joint impact of these drivers on performance when individual attributes are affected in opposing direction. The aim should be to identify those combinations that maximize discrimination between the alternatives’ performance. This will help to formulate scenarios with more opposing characteristics as recommended in the scenario literature [36, 123], allowing even stronger robustness assessment of alternatives.

We spent considerable efforts on defining the scenarios and alternatives, often together with experts. This included making assumptions about the influence of scenarios on the attribute levels so that for each alternative dependable performance predictions could be obtained. Arguably, the uncertainties attached to the predictions were sometimes large and could have been reduced by more detailed assessments. In addition to reducing attribute uncertainty, it would also be worthwhile to study and explicitly model the dependencies between attribute outcomes. Therefore, it would have been necessary not only to elicit the implicit dependencies that experts considered when assessing attribute levels, but also to make substantial assumptions about the dependencies to the other attributes, which we would not have been able to validate. As this was not viable, we resorted to using an independent sample from the marginal distributions instead. The uncertainty analysis shows that such substantial additional efforts would not have yielded a different result. For example, the attribute predictions of innovative technologies are significantly more uncertain than current technologies, because of limited institutional and operational experience with these [17, 23]. This particularly affects those alternatives that comprise decentralized technologies (e.g. A3–A5, A7 for *reliab_hw*, *reliab_ffw*, *aes_hw*, and *aes_dw*; Fig 1). Propagation of these prediction uncertainties did not lead to difficulties in interpreting the ranking of the alternatives in the evaluation model layouts L1–L4. In contrast, it became nearly impossible to visually distinguish the performance of alternatives in layout L5, because including the uncertainties linked to individual stakeholder preferences caused large uncertainty bands and strong overlaps of the overall value distributions (Fig 1). We resorted to plotting the risk profiles to determine whether alternatives were stochastically dominated (Fig 2, section 2.5.2). This allowed us to distinguish between alternatives despite large uncertainty. This is in contrast to a parallel study on wastewater infrastructure planning in the same case study, where the ranking was found to be most sensitive to the uncertainty of the attribute outcome predictions [93].

### 4.4 Improving water supply systems

In a real decision setting, the next steps would be to discuss these insights, to create improved alternatives, predict their performance, and compare them with the already analyzed alternatives. This is ultimately followed by negotiation and decision-making [1, 2, 69]. Since we did not support a full group decision and negotiation process in the case study, we refer the interested reader to the literature on group decision making and negotiation, e.g. the reviews of Kilgour and Eden [124], Kerr and Tindale [125], and Kugler, et al. [126]. Depending on the aim of the decision support intervention, additional stakeholders may need to be included. The advantage of eliciting individual rather than group preferences (regarding weights, values etc.) is that the preferences of additional stakeholders can still be included at any stage of the process. In the remainder, we discuss our main insights from the evaluation of alternatives in the Mönchaltorfer Aa case and how these may be relevant in other cases.

No single alternative is consistently best for all stakeholders in our case study. We assumed that those alternatives perform best which achieve the highest overall value across stakeholders, as there is little conflict potential in their ranking. A6* (*‘Maximal collaboration*, *centralized’*) posits itself as potential compromise. It receives the highest overall value across evaluation layouts, future scenarios, and stakeholders. It is never worse than rank six (of 13) for any individual stakeholder. Its difference in value compared to A1b and A2 is small. A1b and A2 could be good alternatives because transitioning from the current system to one of these more centralized systems might be easier to realize in practice than to a central-decentralized mixed system such as A6*, where decentralized rainwater and firefighting tanks would need to be installed. On the other hand, the long-term costs of both A1b and A2 may become considerably larger than those of A6* or the current system A0, if strong population growth occurs (see *costcap* and *costchange* in Fig 3 for Boom). Alternatives A5, A3, or A9 are the worst alternatives and should thus be discouraged. Regardless of the evaluation model and stakeholder, the current system A0 is clearly outperformed (Figs 1 and 2). Although A0 is not the highest-ranking alternative, the value difference between A0 and better alternatives is small and at a high value level (Table 4). This means that the current system is desirable according to the stakeholder preferences, but also that improvement in the interest of all ten stakeholders is possible.

In our study, alternatives with fully-decentralized technology performed lower than those with centralized technology and the best alternatives combine elements of both. The results show that this is not only a consequence of the technology, but also of their management, organization, and collaboration. Given that many water supply systems share similar technical, organizational, and managerial characteristics with our case study, the following opportunities for improvement may be worth exploring beyond this specific case.

Rehabilitation management may be even more important than the choice of the actual technology. For example, both alternatives A2 and A9 foresee centralized water supply and dimensioning of new water pipes on household peak demand (instead of higher volumes required by fire-fighting regulations). This allows smaller pipe diameters, thereby reducing costs and avoiding water quality issues occurring in current over dimensioned systems due to stagnation [20, 117]. Fire-fighting requirements are met with additional underground firefighting tanks. The main difference between the two alternatives is their maintenance and rehabilitation, which is minimal in A9 and moderate in A2. Due to higher maintenance, alternative A2 performs substantially better than A9 (Fig 1). This is particularly noticeable in layout L3, where no individual acceptance thresholds apply, but also holds in L4b and L5. Conversely, alternatives A5 and A7 are technically comparable, decentralized alternatives. Both foresee in-house water treatment and water delivery by lorries (Table B.3 in S1 File). Yet A7 has moderate rehabilitation efforts as opposed to minimal efforts in A5. As a result, A7 clearly achieves higher values (Fig 1). Planners and utility managers can improve the performance of the current system A0 by embracing pro-active and more extensive rehabilitation management as this most strongly positively affected the ranking of alternatives. Interestingly, rehabilitation management also clearly impacted the ranking of alternatives in the parallel study on wastewater infrastructure planning, despite different attributes and assumptions [93]. This is in line with another study focusing on the comparison of long-term rehabilitation strategies, where we found that alternatives with little or no rehabilitation efforts were outperformed except if almost the entire weight was attributed to costs [98]. The elicited preferences in both this and parallel studies for the wastewater system [93, 127] revealed that stakeholders and the general public assigned little weight to the cost objectives if taking a long-term view on water supply and wastewater system performance. In light of widespread underinvestment in water infrastructure rehabilitation in many countries [10, 12], efforts to improve rehabilitation management towards evidence-based, proactive strategies is highly relevant also beyond our case study.

Furthermore, the results indicate that stronger collaboration across utilities as well as adaptations to the technical dimensioning requirements of the pipe-system benefit performance. The impact of organization and management might seem small given the little weight of the attributes it directly affects: *efqm*, *voice*, *collab* (Table B.2 in S1 File). Comparing alternatives A7 to A3, however, technology and rehabilitation efforts are similar (Table B.3 in S1 File). But in A3, the end users are responsible for maintenance, whereas in A7 the responsibility is shared between utilities and end users. This causes A7 to perform much better on social acceptance (due to *efqm*, *voice*, *collab*; Fig 3) which could explain why A7 outperforms A3. It is striking that A7 has the least fragmented (most centralized) organization and management of all alternatives with decentralized technology, but still has far more fragmented organization than the alternatives with centralized technology (Table B.3 in S1 File). This implies that none of the stakeholder groups considered combining decentralized technologies with centralized organization and management when developing the alternatives [75]. Inversely, no centralized technology foresees a stronger sharing of responsibility with private households. As with the formulation of scenarios, we would recommend to either more strongly guide the participants when developing alternatives or to include these ourselves into the analysis, such that not only innovative technology, but also innovative combinations of management, organization, and technology are encompassed.

The fire-fighting requirements should be revisited, because in small municipalities or peripheral residential areas they often result in bigger pipe diameters than needed to satisfy residential peak demands. Given the advancements in firefighting equipment and practice, but also building materials, lower water flows may be possible. In the Netherlands, firefighting design flows were reduced from 60–90 m^3^/h in the 1990ies to 30 m^3^/h today [20]. This reduces costs and avoids negative impacts on drinking water quality due to stagnation. In the Canton of Zurich, 90 m^3^/h are currently required [128]. This number is established based on conservative estimates by fire brigades’ about expected flows needed to control and extinguish certain types of fires, but not on factual water use [129–131]. Thus, dependable data should be obtained to reassess firefighting needs. The Dutch example shows that this is possible without disturbing ongoing firefighting activities.

Note that the reliability was estimated to be lower in decentralized than in centralized systems (see B.4). It is conceivable that the reliability of innovative technologies will improve in the future, when more practical experience concerning their operation and maintenance becomes available. Further research and practical testing is needed to determine whether comparable reliability can be achieved with the decentralized technologies in e.g. A3–A5, and A7, if monitored and maintained as extensively and professionally as current centralized assets. Hence, one could evaluate whether the decentralized technologies would rank higher under more optimistic reliability assumptions (as in [93], where an alternative with decentralized wastewater technology surfaced as potential best alternative). As with reliability, it remains to be investigated whether alternatives A3–A5, and A7 could perform as well as the centralized technologies, if also assigned with more beneficial organization and management characteristics. We agree with Marlow, et al. [17] that the current evidence base about the performance of decentralized systems in different environments is insufficient to make conclusive judgements. Our and the related wastewater study [93, 127] indicate that decentralized systems for water supply and wastewater treatment may find high acceptance if they can achieve similar performance on these dimensions.

## 5 Conclusions

### 5.1 Evaluation model comparison and selection

We assessed the performance of 13 urban water supply alternatives and demonstrated the implications of five potential evaluation models on rank stability and the ability to discern alternatives. The three MAVT model layouts (L3 –L5) are more recommendable than the two IA models (L1, L2), for both conceptual and technical reasons. In IA as opposed to MAVT, the evaluation model typically encompasses the implicit values and judgement of the analyst and not the preferences of those who are made liable for the decisions. The common assumption of equal weights in IA may also have undesirable consequences, such as a decreasing relevance of individual attributes with increasing number of objectives (L1, non-hierarchical, linear aggregation) or exaggerating the importance of individual attributes, if they belong to a branch of the objectives hierarchy with few sub-objectives (L2, hierarchical, linear aggregation). If acceptance thresholds are incorporated in the model (L4, L5), these should be defined with caution to avoid premature exclusion of good alternatives. In particular, the attribute outcomes should be known before threshold elicitation so that misperceptions about performance of the current situation can be corrected and realistic thresholds defined. Finally, the models’ ability to discern alternatives improves from evaluation model layout L1 to L5 with increasing incorporation of preference components and uncertainties, i.e. model complexity.

Even if the changes in ranking between the five evaluation layouts are not significant in our case, the occurrence of rank reversals on the less salient middle-ranking alternatives cautions us to recommend the use of simpler models or MAVT over other MCDA approaches in general. Rather, if the aim of the analysis is to produce an authoritative ranking, then more complex models that represent stakeholder preferences and alternative performance as closely as possible and include uncertainties are preferable. For being able to derive robust conclusions, the sensitivity of the results to modeling choices needs to be studied in each application. If the intended use of the model is, however, to provide a model for analysis and discussion of key trade-offs within a broader, structured decision-making process (and not a precise ranking of alternatives) then simpler models such as L3 may suffice. It is the responsibility of the analyst to capture, describe, and evaluate the implications of the selected model(s), as well as to suggest appropriate measures to account for uncertainties that matter.

### 5.2 Long-term planning and improvement of water supply systems

Combining an MCDA process with scenario analysis allows to consider dynamic future changes and uncertainties even when their exact magnitude is unknown. This is essential when planning water systems that must function for long time periods under potentially large future uncertainty. It allows not only identification, but also targeted development of robust alternatives, such as A6* in this study. To ensure that all scenarios are useful for exploring the possible outcome space and robustness of alternatives, those combinations of scenario drivers and attribute characteristics need to be identified during scenario development that maximize discrimination between alternatives. This requires to pay special attention to anticipate (i) the magnitude of impact of individual scenario drivers on the performance of alternatives regarding the attributes, (ii) the direction of influence (positive or negative) of these drivers, and (iii) the joint impact of these drivers on performance when individual attributes are affected in opposing direction.

The debate on innovation in the water sector and how to increase the sustainability of existing water supply systems should take a broader look at alternatives, including unconventional ones, and ways to improve these. Our results show that proactive rehabilitation management may well have a stronger impact on the ranking of alternatives than the specific technical system characteristics.

While the current centralized water supply system (A0) performs reasonably well in this case, it is outperformed by three alternatives (A1b, A2, A6*) that are consistently among the best for all stakeholders, future scenarios, and evaluation models. These are centralized or mixed centralized-decentralized systems that foresee at least moderate, proactive maintenance and rehabilitation, strong sectoral and spatial integration, as well as system-wide adaptations to distribution and/or treatment technology. Whether decentralized water supply systems would perform as good as these, if matched with similarly beneficial organization and management and comparable professionality of operation and maintenance, requires further study. In the meantime, reassessing current rehabilitation management, firefighting guidelines, and potentially institutional collaboration are robust recommendations that are worth exploring also in other cases.

## Supporting information

S1 File Supporting information file. Details in the respective sub-chapters on A) scenario assumptions, B) objectives and attributes assessment, C) stakeholder preferences, D) uncertainty and simulation assumptions, and E) additional results.
(PDF)
