# Supplementary material for: Comparing multi-criteria decision analysis and integrated assessment to support long-term water supply planning
Source: PLoS One. 2017 May 8;12(5):e0176663. doi: 10.1371/journal.pone.0176663 (PMC5421771; doi:10.1371/journal.pone.0176663)
Supplement: S1 File — Details in the respective sub-chapters on A) scenario assumptions, B) objectives and attributes assessment, C) stakeholder preferences, D) uncertainty and simulation assumptions, and E) additional results. (PDF) [file pone.0176663.s001.pdf]

Supporting Materials to

Comparing Multi-criteria Decision Analysis and Integrated Assessment to support long-term water supply planning

Lisa Scholten[1], Max Maurer[2,3] and Judit Lienert[2]

[1] Faculty of Civil Engineering and Geosciences, Delft University of Technology, PO Box 5048, 2600 GA Delft, The Netherlands

[2] Eawag: Swiss Federal Institute of Aquatic Science and Technology, Überlandstrasse 133, P.O. Box 611, CH-8600 Dübendorf, Switzerland

[3] ETH Zurich, Institute of Environmental Engineering, CH-8093 Zurich, Switzerland

Introduction

This supporting information provides additional data used to generate the results presented in the main article. These data and results are figures and tables, which we arrange in five parts. Additional data and assumptions regarding the future scenarios used are given in part A. Part B provides detailed summary tables and description of the attribute performance assessed. In part C, we give a summary of the stakeholder preferences used. Supplementary assumptions of the uncertainty simulation can be found in part D. Finally, part E contains supplementary results to those presented in the main text that may also be of interest to the reader. Some of the tables summarize or extend information already published elsewhere and are reprinted with the permission of the original source as cited.

Table of contents

PART A – SCENARIO ASSUMPTIONS 3
Tab. A.1. Main characteristics of the four future scenarios. 3
Tab. A.2. Scenario-dependent water demand. 3

PART B – OBJECTIVES HIERARCHY AND ATTRIBUTE ASSESSMENT 4
Fig. B.1. Objectives hierarchy (adapted from [5], Fig.1). 4
Tab. B.1. Overview of attributes and their assessment 5
Tab. B.2. Cross-impact matrix. 7
Tab. B.3. Decision alternatives (adapted from [5], Tab. SI3.1). 8
Tab. B.4. Predictions of the attributes outlined in Tab. S2.1 (adapted from [5], Tab. SI3.2) 9

PART C – STAKEHOLDER PREFERENCES 15
Tab. C.1. Elicited weights from face-to-face interviews with ten stakeholders 15
Tab. C.2. Value function preferences 15
Tab. C.3. Stated acceptance thresholds and potential preference interactions 15

PART D – ASSUMPTIONS FOR UNCERTAINTY SIMULATION 16
Text D.1. Supplementary modeling assumptions for evaluation layout L5 16

PART E – ADDITIONAL RESULTS 17
Fig. E.1. Attribute outcomes in the Doom and Quality of life scenarios 17
Tab. E.1. Absolute mean weights as used in evaluation model layouts L1-L5. 18
Fig. E.2. Expected values (EV) of alternatives for ten stakeholders. 19
Fig. E.3. Value of alternatives (rows) for stakeholder SH1 in evaluation model layout L3. 20
Fig. E.4. Value of alternatives for stakeholder SH2 in evaluation model layout L3. 20
Fig. E.5. Value of alternatives for stakeholder SH3 in evaluation model layout L3. 21
Fig. E.6. Value of alternatives for stakeholder SH4 in evaluation model layout L3. 21
Fig. E.7. Value of alternatives for stakeholder SH5 in evaluation model layout L3. 22
Fig. E.8. Value of alternatives for stakeholder SH6 in evaluation model layout L3. 22
Fig. E.9. Value of alternatives for stakeholder SH7 in evaluation model layout L3. 23
Fig. E.10. Value of alternatives for stakeholder SH8 in evaluation model layout L3. 23
Fig. E.11. Value of alternatives for stakeholder SH9 in evaluation model layout L3. 24
Fig. E.12. Value of alternatives for stakeholder SH10 in evaluation model layout L3. 24
Tab. E.2. Overall value and ranking of alternatives (A1b–A0) 25

REFERENCES 27

Part A – Scenario assumptions

Tab. A.1. Main characteristics of the four future scenarios.

Name	Socio-economic situation	Population and network development
**Status Quo**	As today: rural region near Zurich with extensive agriculture, leisure areas and nature protection zones. Real income change*: +0.4 %/year	No urban expansion, stable population of ca. 24’200 inhabitants
**Boom**	High prosperity, dense urban development, strong nature protection, new transportation. Real income change*: +4.0 %/year	Rapid urbanization challenges with an increased need for both densification and expansion of urban areas (200’000 inhabitants in 2050).
**Quality of life**	Prosperous region with moderate population growth, limited expansion of building areas, high environmental awareness. Real income change*: +2.0 %/year	Moderate, stable population growth (ca. 29’000 inhabitants in 2050) and only small urban expansion.
**Doom**	Economic recession causes strong financial pressure on municipal budgets, slight population decline but no system expansion/deconstruction. Real income change*: -1.5 %/year	No urban expansion, slight population decrease (ca. 23’000 inhabitants in 2050)

* The mean income in 2008 was 64’575 CHF. With 0.4 % observed increase, the income in 2010 is 65’093 CHF

Tab. A.2. Scenario-dependent water demand.

Demand type	Boom	Doom	Qual. growth	Status quo
**Household (+small businesses) demand Q_{hh}**	135 L/Ed (current demand) [1]	162 L/Ed (demand in 1997) [2]	125 L/Ed (demand with more water-saving appliances, e.g. as in Germany, 2011:122 L/Ed) [3]	135 L/Ed [1]
**Industrial water demand Q_i**	constant per capita amount as in ZH 2009: 146.1 L/Ed [4]	constant per capita amount calculated as 2010 in case study area (corrected for industrial uses in Mönchaltorf): 50.3 L/Ed-	constant per capita amount as 2010 in case study area: 86.4 L/Ed	constant per capita amount as 2010 in case study area: 86.4 L/Ed
**Water loss Q_L**	mean loss over last years: 11 %	11 %	11 %	11 %
**Own consumption Q_E**	Demand for own operations, e.g. flushing, street cleaning etc.: 6.1 %	Demand for own operations, e.g. flushing, street cleaning etc.: 6.1 %	Demand for own operations, e.g. flushing, street cleaning etc.: 6.1 %	Demand for own operations, e.g. flushing, street cleaning etc.: 6.1 %
**Overall water demand Q_d**	$Q_d=Q_{hh}+Q_i+Q_L+Q_E$	$Q_d=Q_{hh}+Q_i+Q_L+Q_E$	$Q_d=Q_{hh}+Q_i+Q_L+Q_E$	$Q_d=Q_{hh}+Q_i+Q_L+Q_E$

Part B – Objectives hierarchy and attribute assessment

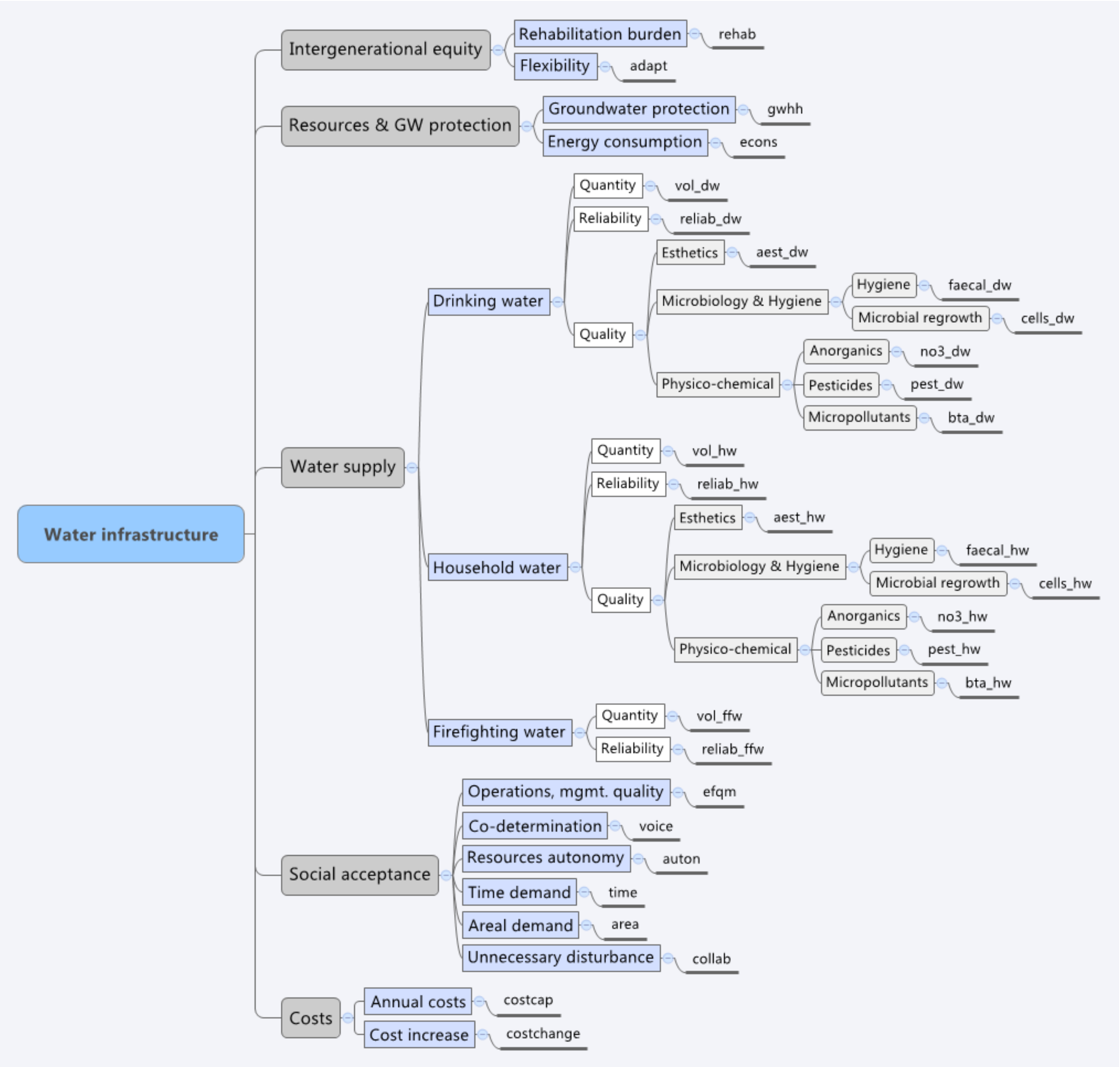

Fig. B.1. Objectives hierarchy (adapted layout from [5], Fig.1). The boxes show higher and lower level fundamental objectives for achieving a good water supply infrastructure. Underlined short names at the right end of the branch are the attributes used to quantify the performance on these objectives. Details, see Tab. B.1.

Tab. B.1. Overview of attributes and their assessment (adapted from [5]; Tab. SI2.1).

Name	Attribute [min-max]	Assessment
rehab	Realization of the rehabilitation demand [0-100⁺ %]	Calculated. Rehabilitation of the centralized pipe water system is modeled in detail following the approach described in [6]. The therein specified prior distribution is used to predict failures for the case study networks as a whole, but without Bayesian inference of failure parameters (because there are no failure records from three of the five case study water networks and because of the little difference between the prior and posterior distribution shown in [6] for water supplier D). The replacement of treatment, pumping, and storage facilities of the centralized and decentralized treatment system are not considered given their much shorter lifetimes and higher immediacy. Partial replacements are often performed during usual maintenance. For these assets, a 100% realization of the rehabilitation demand within one generation is assumed.
adapt	Flexibility of technical extension or deconstruction of infrastructure [0-100⁺ %]	Expert assessment. At first, all alternatives were judged individually by four participating engineers. Their judgment was incurred concerning how easy it would be to technically extend or to deconstruct the respective infrastructure. Thereto each participant received a form with a description of the relevant aspects characterizing the alternatives, namely: organizational structure, construction and operation of water infrastructure, wastewater system technology, and drinking water system technology. The participant assigned one out of the five categories very low (0- 20%), low (20- 40%), medium (40- 60%), high (60- 80%), very high (80- 100%) system flexibility to each alternative. Then, the mean of the participants' judgments and the standard deviation were calculated (using the mid-points of the categories' intervals, i.e., 10, 30, 50, 70, and 90%). Those alternatives with more than 10% deviation were subsequently discussed. The group members with the highest divergence explained the argumentation for their judgments. After this was done, a final score was assigned to each alternative by the overall group. Larger interval ranges depict higher uncertainty or higher variance between the group member's judgments. These results were sent to an external expert (Fraunhofer Institute for Systems and Innovation Research ISI, Karlsruhe, Germany) for validation.
gwhh	% Utilization of groundwater recharge [⁺0-180 %]	Calculated as groundwater abstraction/groundwater recharge. Groundwater recharge was estimated using the Hydrus1D model for simplified soil profiles, representing the characteristics of predominant soils in the case study region. Climate data (MeteoSwiss 2011) and delta change scenarios for ten different regional climate models were used [7, 8]. Based on these, rain series were generated in a collaboration project [9] using a weather generator [10] following the description of [11]. The minimum and maximum resulting range for groundwater recharge per m^2 was used. The political area of the case study is used as a reference, i.e. groundwater abstraction and recharge are calculated as per m^2 of political land area. The amount of groundwater abstraction depends on the scenario and alternative.
econs	Net energy consumption for water treatment and transport [⁺0-2 kWh/m^3]	Calculated. The best case (low energy consumption) is assumed as zero, because of little / no treatment of water and wastewater, and the use of gravity for transport. The worst case (maximum energy consumption) was calculated assuming very energy-intensive water treatment, and water withdrawal and transport over long distances requiring pumps and tank wagons. To transport bottled water, mineral oil equivalents were converted to energy. For wastewater, we assumed the energy consumption of high-tech decentralized treatment units, and added the energy consumption for the removal of micropollutants and the treatment of urine (and a safety factor). Energy demand for water treatment and distribution is calculated based on assumptions from [12] for different centralized treatment and distribution systems. Energy demand for advanced oxidation processes origins from [13]. Energy for household pumping and treatment is calculated according to producer specifications of selected decentralized installations. Energy demand for lorries is taken from [14]. Bottled water is presumably bought together with other goods and thus its impact regarding energy (fuel) consumption was neglected.
vol_dw, vol_hw,<br><br>vol_ffw	Days per year with water quantity limitations [⁺0-365 d/a]<br><br>FFW: Available water for firefighting in new housing areas [500-3600⁺ L/min]	Calculated. Whether a system is prone to water quantity limitations or not depends on its dimensioning and the expected demand. Centralized pipe systems were dimensioned on peak demands and are thus less prone to quantity limitations than decentralized tank options dimensioned on satisficing average daily demands. Following explanations of one of the local engineering consultants, the currently used peak hourly demand amounts to 450 L/(inhabitant*d) which is considerably less than the amounts used in the past (around 550-800 L/(inh.*d)), but sufficient to cover past residential peak demands in the case study water networks. Only in the network of one water supplier, the measured peak demand over the last decade is 471.4 L/(Ed). Except of this single event, on 99.7% of days between 2007-2010, the water demand amounted to less than 390.3 L/(inh.*d). Hence it is assumed, that the centralized pipe network is not likely to expect water quantity restrictions if dimensioned to that peak demand (450 L/(inh.*d), peak hour demand = 10% of peak days). If the decentralized systems are delivery on demand systems (or buying water in the supermarket), it is also assumed that quantity limitations are unlikely. In the case of alternative A4, in the Boom scenario, water is refilled in regular, weekly intervals. Using the rain time series generated for the predictions of groundwater recharge (see gwhh) and assuming a completely filled rainwater tank at the beginning, the number of days with quantity restrictions are counted.
reliab_dw, reliab_hw, reliab_ffw	System reliability (in interviews termed criticality) [⁺0-0.25]	Calculated. The estimates of system reliability are based on the probability of failure, which is modeled in detail for the centralized pipe system and the criticality of different assets. In decentralized systems, a discrete scale is used. As orientation, the classification of failure rates in decentralized wastewater systems as reported in [15] is used. It classifies the annual probability of failure as associated to a qualitative judgment from very high (failure rate (FR): >1-1) over high (FR: 0.5- 0.33), moderate (FR: 0.25-0.1), and rare (0.05-0.03) to extremely rare (FR 0.02).
aes_dw, aes_hw	Days per year with esthetic impairment such as taste, smell, etc. [⁺0-365 d/a]	Expert assessment. An expert from the Zurich cantonal laboratory provided the estimates. Thereto, two meetings were convened, before the expert assessed the alternatives. In the first meeting, characteristics of the case study area, the alternatives, and the future scenarios were presented and discussed. Factors that influence the attribute were discussed. The expert defined which additional information he needed to provide estimates for the attribute levels. In the second meeting, the requested additional information and detailed characteristics of the alternatives were presented and discussed.
faecal_dw, faecal_hw	Days per year with hygienic concerns (hygiene indicators) [⁺0-365 d/a]	Expert assessment. Expert and assessment as for aes_dw, aes_hw.
cells_dw,	Changes in total cell count as	Expert assessment. Expert and assessment as for aes_dw, aes_hw, but with an additional estimate of an expert at Eawag (specialist in flow cytometric cell counts).

Name	Attribute [min-max]	Assessment
cells_hw	indicator of bacterial regrowth [+0-2 log units]	The estimate of both experts were combined, i.e. the overall average, maximum, and minimum values were used.
no3_dw, no3_hw	Inorganic substances (indicator: nitrate concentration) [+0-20 mg/L]	Attribute ranges. Time did not suffice to estimate this attribute in detail. Hence, minimum and maximum attribute ranges are used. These stem from the measured concentrations in the different raw waters in the case study region [16] and lake water at Stäfa [17], and the minimum and maximum mixing ratios of these. It is assumed that some treatment can be found which might lead to a complete removal of nitrate.
pest_dw, pest_hw	Pesticides (sum of pesticide concentration) [+0-0.02 µg/L]	Attribute ranges. Time did not suffice for detailed assessments. The minimum and maximum attribute ranges are used. They stem from measured concentrations in different raw waters of the case study region [16] and lake water at Stäfa [17] and the minimum and maximum mixing ratios of these. It is assumed that some treatment can be found which might lead to a complete removal of pesticides.
bta_dw, btw_hw	Micropollutants (indicator: benzotriazole) [+0-150 ng/L]	Attribute ranges. Time did not suffice for detailed assessments. The minimum and maximum attribute ranges are used. They stem from measured concentrations in the different raw waters and the minimum and maximum mixing ratios. It is assumed that some treatment leads to a complete benzotriazole removal.
efqm	Score of the EFQM excellence model (European Foundation for Quality Management) [20-95+%]	Expert assessment. For details concerning the model see EFQM [18]. An expert from Eawag provided the estimates. The same procedure as for aes_dw, aes_hw, cells_dw, cells_hw was followed. Through nine criteria, the EFQM Excellence Model helps companies understand and analyze the cause and effect relationships between what the organization does and the results it achieves. Five of these criteria are 'Enablers' and four are 'Results'. 'Enabler' criteria cover what an organization does and how it does it. 'Results' criteria cover what an organization achieves [18]. Each alternative is assessed separately, assigning up to 100 points each, to be normalized to a range of 0-100%. The results criteria were discarded as the expert judged a fictitious judgment of future results based on organization from and spatial extent pointless.
voice	Degree (percent) of codetermination [0-100+ %]	Expert assessment. Two experts from Eawag provided estimates. After information and discussion about the alternatives and future scenarios, all alternatives were assessed individually by the expert. They assigned one of five categories very low (0- 20%), low (20- 40%), medium (40- 60%), high (60- 80%), very high (80- 100%) to each alternative. The estimates of both experts were integrated to get an overall minimum, maximum, and average value.
auton	Portion of water coming from the Mönchaltorfer Aa region [0-100+ %]	Calculated. The percentage of water abstracted from sources and wells in the case study region depends on the alternative and the water demand. Water demand covers household, industry, and business demand as well as water losses. It is calculated depending on the future scenario and alternative. Imported water from the regional water supply cooperative (surface water from lake Zurich) is considered 'external' and reduces the case study's resources autonomy.
time	Necessary time investment for operation and maintenance by user [+0-10 h/(inh.*a)]	Calculated. Only applies to decentralized installations in private households that the end user takes care of. Necessary operation and maintenance times depend on the water supply facilities as specified by the alternative and following dimensioning for different building units. Time demands are specified by installation and building unit, added up and then divided by the number of inhabitants sharing a unit. Building units are areas of approximately similar housing and density. The existing building areas in the case study were summarized into 10 building units, 5 for the Status quo/Doom scenario, 3 for the Boom scenario, 2 for the Quality of life scenario. A weighted mean over all building units is calculated for estimation.
area	Additional area demand on private property per end user [+0-10 m²/inh.]	Calculated. Only applies to decentralized installations in private households with additional space needs. The different installations are dimensioned for predefined building units (see explanation under time) and then the area demand for each building unit can be calculated. The area per building unit is divided by the number of inhabitants in the building unit and a weighted mean calculated over all building units in the case study area.
collab	Number of infrastructure sectors that collaborate in planning and construction [1-6+]	Direct consequence of the alternative definition. The number of collaborating infrastructure sectors is equal to that specified in the alternative description , see Tab. S.3 and [19].
costcap	Annual cost per inhabitant in% of the mean taxable income [+0.01-5 %]	Calculated. Annual costs 2010-2050 were estimated using unit costs for expansion, rehabilitation, operation, and maintenance specified for:<br>*Fees:* imported water fees (from regional water supplier), bottled water fees, water lorry delivery fee<br>*Operation and maintenance:* centralized water supply system, decentralized water storage (household tanks), decentralized firefighting tanks, point-of-entry (POE) treatment system, point-of-use (POU) treatment system, rainwater filters, decentralized tank chlorination.<br>*Expansion of or reinvestment:* pipe rehabilitation, pipe network expansion, central water purification plant (WPP), central water reservoirs, central UV treatment, decentralized water storage (household tanks), decentralized firefighting tanks, POE systems, POU systems, rainwater filters, decentralized tank chlorination.
costchange	Mean annual (linear) increase of costs [+0-5 %/a]	Calculated. Derived from costcap using the annual linear increase of costs between 2010-2050.

Tab. B.2. Cross-impact matrix. Characteristics of alternatives and future scenarios and impact on attribute prediction. Grey-shaded fields indicate characteristics that are assumed to affect the attribute predictions directly.[1]

	**ORGANIZATION & SPATIAL EXTENT**				**MANAGEMENT**				**TECHNOLOGY**			**SCENARIO**			
**Attribute**	**Organization form**	**Sector cooperation**	**Responsibilities (intake, treatment , distribution etc.)**	**Collaboration between municipalities**	**Funding strategy**	**Rehabilitation strategies and measures**	**Pipe/sewer laying technique**	**Operation, maintenance, inspection, monitoring**	**Raw water source and purpose of use**	**Distribution system**	**Water treatment**	**Population growth and spatial development**	**Environmental awareness, water demand**	**Nominal income increase, financial situation**	**Climate scenarios**
Rehab						■		■				■			
Adapt	■					■		■	■	■	■				
Gwhh									■			■	■		■
Econs									■	■	■				
Vol_dw/_hw/_ffw									■	■			■		■
Reliab_dw/_hw/_ffw						■		■	■	■	■	■			
Aes_dw/hw						■			■	■	■				
Faecal_dw/_hw						■			■	■	■				
Cells_dw/_hw						■			■	■	■				
NO3_dw/_hw															
Pest_dw/_hw															
Bta_dw/hw															
Efqm	■	■	■	■										■	
Voice	■	■	■	■	■										
Auton									■			■	■		■
Time									■	■	■	■			
Area									■	■	■	■			
Collab		■													
Costcap						■	■	■	■	■	■	■	■	■	■
Costchg						■	■	■	■	■	■	■	■	■	■

[1] The clustering of attributes indicates that attributes are affected by the same influencing factors and not that their attribute outcomes are identical. This is because the attribute outcomes depend on both the influences under different scenarios and the configuration of the alternatives.

Tab. B.3. Decision alternatives (adapted from [5], Tab. SI3.1). UV= ultra-violet disinfection; AOP= advanced oxidation process; GAC= granular activated carbon; POE= Point-of-entry treatment (in cellar), POU= Point-of-use (under sink), O3= ozone, UF= ultrafiltration, RO= reverse osmosis.

	**A0**<br>*Current system*	**A1a / A1b**<br>*Centralized, privatization, high environmental protection*	**A2**<br>*Centralized IKA, rain stored*	**A3**<br>*Fully decentralized*	**A4**<br>*Decaying centra-lized infrastructure, decentralized outskirts*	**A5**<br>*Decaying infrastructure everywhere*	**A6 / A6***<br>*Centralized, maximal collaboration*	**A7**<br>*Mixed responsibility, fully decentralized with on-site treatment*	**A8a/ A8b**<br>*Status quo with storm water retention*	**A9**<br>*Centralized, privatization, minimal maintenance*
**Organization, cooperation, mgmt..**	Five individual water utilities, two municipally governed, three organized as cooperatives. No cross-sector integration [a].	A1a: A private contractor manages all sectors [a] and municipalities) (withi n larger region). A1b: As A1a, but managed by intercommunal agency (IKA).	As A1a, but managed by intercommunal agency IKA) and with constant budget, 100% self-financed.	All sectors[a]/ communities[b] work separately. Households are responsible and contract service to external companies.	Cooperatives, municipalities, households. No sector inte-gration). Out-side area of 2010: consumers are responsible, buy external companies' services	Most infrastruc-ture services are responsibility of households. Services are contracted to external organizations.	Maximal cooperation; case study communities [b] and Oetwil a.S. as one cooperative; combined services for water, waste-water, electricity, gas, roads and tele-communication.	Single coopera-tive for water and wastewater services in all municipalities[b]. No cross-sector integration[a]. Private owners responsible for facilities on private grounds.	Municipalities[b] responsible for a single, integrated wastewater and drinking water sector that jointly operates WIS; some services contracted out to private enterprises.	The water infrastructures are fully contracted out, all sectors work separately[a]. Private consumers choose their contracting provider.
**Operation and maintenance**	Mixed, mostly reactive. Age-dependent re-placement and inspections.	Replacement (2%/a) by con-dition (service galleries). Exten-sive operation & maintenance; average inspection.	Replacement by condition (1%/a) with less costly laying technique. Moderate operation, maintenance, and inspection.	Only repairs upon urgent need for action, no replacement undertaken. Moderate operation and maintenance; little inspection.	As A3, but operation and maintenance is minimal.	Measures are only undertaken upon urgent need for action, operation and maintenance are minimal (as A4), and no inspection at all.	Replacement according to condition (1%/a). Repair and replacement in trench. Moderate operation, maintenance, and inspection efforts.	Replacement according to prioritization. No replacement of centralized assets. Moderate operation, maintenance, and inspection.	Replacement (1%/a) by condition and criticality). Renovation is trenchless. Mode-rate operation, maintenance, and inspection.	Measures only undertaken upon urgent need for action. Minimal operation, maintenance and inspection.
**Water supply and uses**	Centrally treated and supplied for potable, house-hold, and fire-fighting use. Dimensioning based on maximum expected peak demands (mostly fire-fighting).	Centrally treated/supplied for potable, household, and firefighting use. Dimensioning as usual based on maximum expected peak demands (mostly fire-fighting).	Centrally treated/supplied for potable, household, and firefighting use. Dimensioning on maximum hourly house-hold demand. Decentralized fire-fighting water tanks.	Potable water from the super-market, house-hold water treated in house-holds and deli-vered by water lorries. Fire-fighting water volumes also kept in house-hold water tank.	Water for all purposes is centrally supplied in the area of 2010 (drinking water quality not ensured). In the outskirts, water is supplied by lorries once per week.	No centralized water supply, no new pipes. Consumers operate tanks recharged by a private delivery service (lorries). Hygienically safe water. Separate fire-fighting water tanks.	Centralized supply of drinking and household water. Decentralized reuse of rainwater for toilet flushing. Dimensioning is on the maximum hourly demand of households, further volumes for firefighting are held in decentralized, under-ground firefighting water tanks.	Rainwater used in households where possible. Further water will only be delivered by the municipality (lorries) upon special demand. Fire-fighting tanks are shared between neigh-boring lots.	A8a: Centralized treatment and supply for all water uses. A8b:as A8a, but new housing areas are dimensioned on 30 m^3/h fire flows – similar to 'self-cleaning networks' [20]	Centralized treatment and supply for all water uses. New areas dimensioned on max. household demand per hour. Decentralized fire-fighting water tanks.
**Water sources**	2010 amounts from springs/ groundwater, supplemented with purified lake water from regional water supplier.	As A0.	As A0, Rainwater is used as far as possible for filling firefighting water tanks.	Household and firefighting: filtrated rain-water & treated greywater; Drin-king: bottled water. Other: regional source.	2010 amounts, if not enough, more water from regional water supplier (lake water).	All water is abstracted from springs and groundwater wells in the region.	A6: Withdrawal from sources and wells topped-up by rainwater; max. 10% from regional supplier (lake water). Rainwater for washing, toilet flushing. A6*: s.A0.	As A0, but rain-water used where possible. Reduced water demand due to urine diversion.	As A0.	As A0.
**Treatment technology**	Untreated groundwater, lake water (multi-step treatment with O3-GAC).	Groundwater disinfection with UV; lake water treatment (multi-step treatment with AOP+GAC).	Groundwater disinfection with UV; lake water treatment (multi-step treatment with AOP+GAC).	As A0. Bottled water. Rainwater & grey water are purified (POE system using GAC+UF).	As A0. Household POU drinking water treatment (GAC-RO filter)	In-house hygienization of tank water (chlorination).	As A0. Rainwater is coarsely filtrated at the inflow to the rainwater tank.	POE treatment (GAC+UF) of all incoming water.	As A0, but with groundwater disinfection (UV treatment).	As A0, but with groundwater disinfection (UV treatment).

[a] With all sectors, we mean transportation, gas supply, energy supply, district heating, telecommunication, as well as water supply and wastewater disposal. [b] Mönchaltorf, Gossau, Grüningen, Egg.

Tab. B.4. Predictions of the attributes outlined in Tab. S2.1 (adapted from [5], Tab. SI3.2) by alternative and scenario, stated as probability distributions (adapted from [5], Tab SI3.2). Explanation of abbreviations: A1a – A9…alternatives; see Tab. S3.1 for a description; Status quo, Boom, Doom, Quality of life are the four socio-demographic future scenarios; DW… drinking water; HW… household water; FFW…firefighting water; β(x,y)…beta distribution with shape1 = x, shape2= y; N(x,y)…normal distribution with μ= x, σ= y; LN(x,y)…lognormal distribution with μ = x, σ= y; WB(x,y)…Weibull distribution with shape = x and scale = y; LOG(x,y)…logistic distribution with location = x, scale= y; U(x,y)…uniform distribution with min = x, max= y; TN(x,y [a,b])…truncated normal distribution with μ = x, σ = y and truncation at min= a, max = b.

	A1a	A1b	A2	A3	A4	A5	A6	A6*	A7	A8a	A8b	A9	A0
**Realization of the rehabilitation demand [%] (rehab)**													
**Status quo**	β(9.03875, 4.0951)	β(9.0375, 4.0951)	β(19.0754, 8.9788)	U(0,0)	U(0,0)	U(0,0)	β(19.075 8.979	β(19.075 8.979	U(0,0)	N(0.0438, 0.0162)	N(0.0438, 0.0162)	U(0,0)	WB(7.886,0.246)
**Boom**	N(0.2486, 0.0814)	N(0.2486, 0.0814)	N(0.2027, 0.0744)	U(0,0)	U(0,0)	U(0,0)	N(0.2027, 0.0744)	N(0.2027, 0.0744)	U(0,0)	β(9.7487, 110.0828)	β(9.7487, 110.0828)	U(0,0)	LN(-2.56, 0.58)
**Doom**	β(9.0375, 4.0951)	β(9.0375, 4.0951)	β(19.0754, 8.9788)	U(0,0)	U(0,0)	U(0,0)	β(19.0754, 8.9788)	β(19.0754, 8.9788)	U(0,0)	N(0.0438, 0.0162)	N(0.0438, 0.0162)	U(0,0)	WB(7.886,0.246)
**Quality of life**	N(0.5692, 0.1517)	N(0.5692, 0.1517)	N(0.5212, 0.1261)	U(0,0)	U(0,0)	U(0,0)	N(0.5212, 0.1261)	N(0.5212, 0.1261)	U(0,0)	LOG(0.074, 0.0088)	LOG(0.074,0.0088)	U(0,0)	N(0.18, 0.055)
**Flexibility of technical extension or deconstruction of infrastructure [%] (adapt)**													
**Status quo**	N(35,7.65)	N(40,10.2)	N(20,10.2)	N(85,7.65)	N(62.5,6.38)	N(62.5,6.38)	N(55,7.65)	N(55,7.65)	N(65,7.65)	N(35,7.65)	N(35,7.65)	N(30,10.2)	N(35,7.65)
**Boom**	N(35,7.65)	N(40,10.2)	N(20,10.2)	N(85,7.65)	N(62.5,6.38)	N(62.5,6.38)	N(55,7.65)	N(55,7.65)	N(65,7.65)	N(35,7.65)	N(35,7.65)	N(30,10.2)	N(35,7.65)
**Doom**	N(35,7.65)	N(40,10.2)	N(20,10.2)	N(85,7.65)	N(62.5,6.38)	N(62.5,6.38)	N(55,7.65)	N(55,7.65)	N(65,7.65)	N(35,7.65)	N(35,7.65)	N(30,10.2)	N(35,7.65)
**Quality of life**	N(35,7.65)	N(40,10.2)	N(20,10.2)	N(85,7.65)	N(62.5,6.38)	N(62.5,6.38)	N(55,7.65)	N(55,7.65)	N(65,7.65)	N(35,7.65)	N(35,7.65)	N(30,10.2)	N(35,7.65)
**% Utilization of groundwater recharge [%] (gwhh)**													
**Status quo**	N(6.45,1.08)	N(6.45,1.08)	N(6.45,1.08)	N(5.32,0.89)	N(6.45,1.08)	N(11,1.84)	N(8.49,1.42)	N(6.45, 1.08)	N(6.45,1.08)	N(6.45,1.08)	N(6.45,1.08)	N(6.45,1.08)	N(6.45, 1.08)
**Boom**	N(7.51,1.25)	N(7.51,1.25)	N(7.51,1.25)	N(81.66,13.64)	N(7.51,1.25)	N(134.69, 22.49)	N(118.96, 19.87)	N(7.51,1.25)	N(7.51,1.25)	N(7.51,1.25)	N(7.51,1.25)	N(7.51,1.25)	N(7.51, 1.25)
**Doom**	N(6.45,1.08)	N(6.45,1.08)	N(6.45,1.08)	N(3.57,0.6)	N(6.45,1.08)	N(10.55,1.76)	N(7.84,1.31)	N(6.45,1.08)	N(6.45,1.08)	N(6.45,1.08)	N(6.45,1.08)	N(6.45,1.08)	N(6.45,1.08)
**Quality of life**	N(6.50,1.09)	N(6.5,1.09)	N(6.5,1.09)	N(6.37,1.06)	N(6.37,1.06)	N(12.71,2.12)	N(9.93,1.66)	N(6.50,1.09)	N(6.5,1.09)	N(6.5,1.09)	N(6.5,1.09)	N(6.5,1.09)	N(6.5, 1.08)
**Net energy consumption for water treatment and transport [kWh/m³] (econs)**													
**Status quo**	N(0.713, 0.1783)	N(0.713, 0.1783)	N(0.713, 0.1783)	N(0.0777, 0.0194)	N(0.4,0.1)	N(0.3649, 0.0912)	N(0.55, 0.1375)	N(0.55,0.24)	N(0.185, 0.0462)	N(0.67, 0.1675)	N(0.67, 0.1675)	N(0.67, 0.1675)	N(0.5,0.125)
**Boom**	N(0.713, 0.1783)	N(0.713, 0.1783)	N(0.713, 0.1783)	N(0.119, 0.0298)	N(0.2996, 0.0749)	N(0.3649, 0.0912)	N(0.55, 0.1375)	N(0.55,0.24)	N(0.2654, 0.0664)	N(0.67, 0.1675)	N(0.67, 0.1675)	N(0.67, 0.1675)	N(0.5,0.125)
**Doom**	N(0.713, 0.1783)	N(0.713, 0.1783)	N(0.713, 0.1783)	N(0.0898, 0.0225)	N(0.4,0.1)	N(0.3649, 0.0912)	N(0.55, 0.1375)	N(0.55,0.24)	N(0.2148, 0.0537)	N(0.67, 0.1675)	N(0.67, 0.1675)	N(0.67, 0.1675)	N(0.5,0.125)
**Quality of**	N(0.713,	N(0.713,	N(0.713,	N(0.0778,	N(0.4,0.1)	N(0.3649,	N(0.55,	N(0.55,0.24	N(0.1797,	N(0.67,	N(0.67,	N(0.67,	N(0.5,0.125)

	A1a	A1b	A2	A3	A4	A5	A6	A6*	A7	A8a	A8b	A9	A0
**life**	0.1783)	0.1783)	0.1783)	0.0194)		0.0912)	0.1375)	)	0.0449)	0.1675)	0.1675)	0.1675)	
**DW: Days per year with water quantity limitations [d/a] (vol_dw)**													
**Status quo**	U(0,0)	U(0,0)	U(0,0)	U(0,0)	U(0,0)	U(0,0)	U(0,0)	U(0,0)	U(0,0)	U(0,0)	U(0,0)	U(0,0)	U(0,0)
**Boom**	U(0,0)	U(0,0)	U(0,0)	U(0,0)	U(0,0)	U(0,0)	U(0,0)	U(0,0)	U(0,0)	U(0,0)	U(0,0)	U(0,0)	U(0,0)
**Doom**	U(0,0)	U(0,0)	U(0,0)	U(0,0)	U(0,0)	U(0,0)	U(0,0)	U(0,0)	U(0,0)	U(0,0)	U(0,0)	U(0,0)	U(0,0)
**Quality of life**	U(0,0)	U(0,0)	U(0,0)	U(0,0)	U(0,0)	U(0,0)	U(0,0)	U(0,0)	U(0,0)	U(0,0)	U(0,0)	U(0,0)	U(0,0)
**HW: Days per year with water quantity limitations [d/a] (vol_hw)**													
**Status quo**	U(0,0)	U(0,0)	U(0,0)	U(0,0)	U(0,0)	NU0,0)	U(0,0)	U(0,0)	U(0,0)	U(0,0)	U(0,0)	U(0,0)	U(0,0)
**Boom**	U(0,0)	U(0,0)	U(0,0)	U(0,0)	N(18.66, 0.9006)	U(0,0)	U(0,0)	U(0,0)	U(0,0)	U(0,0)	U(0,0)	U(0,0)	U(0,0)
**Doom**	U(0,0)	U(0,0)	U(0,0)	U(0,0)	U(0,0)	U(0,0)	U(0,0)	U(0,0)	U(0,0)	U(0,0)	U(0,0)	U(0,0)	U(0,0)
**Quality of life**	U(0,0)	U(0,0)	U(0,0)	U(0,0)	U(0,0)	U(0,0)	U(0,0)	U(0,0)	U(0,0)	U(0,0)	U(0,0)	U(0,0)	U(0,0)
**FFW: Available water for firefighting in new housing areas [L/min] (vol_ffw)**													
**Status quo**	N(1766.968, 442)	N(1766.968, 442)	N(1310.211, 328)	N(1726.288, 432)	N(1766.968, 442)	N(1838.676, 460)	N(1310.211, 328)	N(1310.211, 328)	N(1838.676, 460)	N(1766.968, 442)	N(1766.968, 442)	N(1310.211, 32)8	N(1766.968, 442)
**Boom**	N(3600,900)	N(3600,900)	N(3600,900)	N(2902.984, 726)	N(3600,900)	N(3600,900)	N(3600,900)	N(3600,900)	N(3600,900)	N(3600,900)	N(3600,900)	N(3600, 900)	N(3600,900)
**Doom**	N(1854.309, 464)	N(1854.309, 464)	N(1497.555, 375)	N(1791.37, 448)	N(1854.309,464)	N(1960.12, 491)	N(1497.555, 375)	N(1497.555, 375)	N(1960.12, 491)	N(1854.309, 464)	N(1854.309, 464)	N(1497.555,375)	N(1854.309, 464)
**Quality of life**	N(1766.968, 442)	N(1766.968, 442)	N(1310.211, 328)	N(1726.288, 432)	N(1766.968, 442)	N(1838.676, 460)	N(1310.211, 328)	N(1310.211, 328)	N(1838.676, 460)	N(1766.968, 442)	N(1766.968, 442)	N(1310.211,328)	N(1766.968, 442)
**DW: System reliability (in interviews: "criticality") [-] (reliab_dw)**													
**Status quo**	LN(-5.2162, 0.2991)	LN(-5.2162, 0.2991)	LN(-5.1793, 0.3056)	U(0.98,1)	N(0.0827, 0.0161)	N(0.175, 0.0375)	LN(-5.1793, 0.3056)	LN(-5.1793, 0.3056)	N(0.065, 0.0175)	LN(-4.2198, 0.3378)	LN(-4.2198, 0.3378)	LN(-4.0617, 0.3748)	LN(-4.317,0.377)
**Boom**	β(2.5936, 694.7973)	β(2.5936, 694.7973)	β(2.7087, 689.5533)	U(0.98,1)	U(0.98,1)	N(0.175, 0.0375)	β(2.7087, 689.5533)	β(2.7087, 689.5533)	N(0.065, 0.0175)	β(2.8013, 680.5096)	β(2.8013, 680.5096)	β(3.0522, 653.4647)	LN(2.823,632.05)
**Doom**	LN(-5.2162, 0.2991)	LN(-5.2162, 0.2991)	LN(-5.1793, 0.3056)	U(0.98,1)	N(0.0827, 0.0161)	N(0.175, 0.0375)	LN(-5.1793, 0.3056)	LN(-5.1793, 0.3056)	N(0.065, 0.0175)	LN(-4.2198, 0.3378)	LN(-4.2198, 0.3378)	LN(-4.0617, 0.3748)	LN(-4.317,0.38)
**Quality of life**	Beta(4.073, 688.1364)	Beta(4.073, 688.1364)	LN(-5.1757, 0.4138)	U(0.98,1)	N(0.0897, 0.0171)	N(0.175, 0.0375)	LN(-5.1757, 0.4138)	LN(-5.1757, 0.4138)	N(0.065, 0.0175)	LN(-4.7867, 0.3619)	LN(-4.7867, 0.3619)	LN(-4.5669, 0.3502)	LN(-4.705,0.38)
**HW: System reliability (in interviews: "criticality") [-] (reliab_hw)**													
**Status quo**	LN(-5.2162,	LN(-5.2162,	LN(-5.1793, 0.3056)	N(0.065, 0.0175)	LN(-4.0617, 0.3748)	N(0.175, 0.0375)	LN(-5.1793,	LN(-5.1793,	N(0.175, 0.0375)	LN(-4.2198;	LN(-4.2198, 0.3378)	LN(-4.0617, 0.3748)	LN(-4.317,0.377)

	A1a	A1b	A2	A3	A4	A5	A6	A6*	A7	A8a	A8b	A9	A0
	0.2991)	0.2991)					0.3056)	0.3056)		0.3378)			
**Boom**	β(2.5936, 694.7973)	β(2.5936, 694.7973)	β(2.7087, 689.5533)	N(0.065, 0.0175)	N(0.0878, 0.0163)	N(0.175, 0.0375)	β(2.7087, 689.5533)	β(2.7087, 689.5533)	N(0.175, 0.0375)	β(2.8013, 680.5096)	β(2.8013, 680.5096)	β(3.0522, 653.4647)	LN(2.823,632.05)
**Doom**	LN(-5.2162, 0.2991)	LN(-5.2162, 0.2991)	LN(-5.1793, 0.3056)	N(0.065, 0.0175)	LN(-4.0617, 0.3748)	N(0.175, 0.0375)	LN(-5.1793, 0.3056)	LN(-5.1793, 0.3056)	N(0.175, 0.0375)	LN(-4.2198, 0.3378)	LN(-4.2198, 0.3378)	LN(-4.0617, 0.3748)	LN(-4.317,0.38)
**Quality of life**	β(4.073, 688.1364)	β(4.073, 688.1364)	LN(-5.1757, 0.4138)	N(0.065, 0.0175)	N(0.055, 0.0107)	N(0.175, 0.0375)	LN(-5.1757, 0.4138)	LN(-5.1757, 0.4138)	N(0.175, 0.0375)	LN(-4.7867, 0.3619)	LN(-4.7867, 0.3619)	LN(-4.5669, 0.3502)	LN(-4.705,0.38)
**FFW: System reliability (in interviews: "criticality") [-] (reliab_ffw)**													
**Status quo**	LN(-5.2162, 0.2991)	LN(-5.2162, 0.2991)	LN(-5.1793, 0.3056)	N(0.065, 0.0175)	LN(-4.0617, 0.3748)	N(0.175, 0.0375)	LN(-5.1793, 0.3056)	LN(-5.1793, 0.3056)	N(0.065, 0.0175)	LN(-4.2198, 0.3378)	LN(-4.2198, 0.3378	LN(-4.0617, 0.3748)	LN(-4.317,0.377)
**Boom**	β(2.5936, 694.7973)	β(2.5936, 694.7973)	β(2.7087, 689.5533)	N(0.065, 0.0175)	N(0.0638, 0.0118)	N(0.175, 0.0375)	β(2.7087, 689.5533)	β(2.7087, 689.5533)	N(0.065, 0.0175)	β(2.8013, 680.5096)	β(2.8013 680.5096	β(3.0522, 653.4647)	LN(2.823,632.05)
**Doom**	LN(-5.2162, 0.2991)	LN(-5.2162, 0.2991)	LN(-5.1793, 0.3056)	N(0.065, 0.0175)	LN(-4.0617, 0.3748)	N(0.175, 0.0375)	LN(-5.1793, 0.3056)	LN(-5.1793, 0.3056)	N(0.065, 0.0175)	LN(-4.2198, 0.3378)	LN(-4.2198, 0.3378)	LN(-4.0617, 0.3748)	LN(-4.317,0.38)
**Quality of life**	β(4.073, 688.1364)	β(4.073, 688.1364)	LN(-5.1757, 0.4138)	N(0.065, 0.0175)	LN(-3.2535, 0.2143)	N(0.175, 0.0375)	LN(-5.1757, 0.4138)	LN(-5.1757, 0.4138)	N(0.065, 0.0175)	LN(-4.7867, 0.3619)	LN(-4.7867, 0.3619)	LN(-4.5669, 0.3502)	LN(-4.705,0.38)
**DW: Days per year with esthetic impairment such as taste, smell, etc.[d/a] (aes_dw)**													
**Status quo**	N(5,2.55)	N(5,2.55)	N(5,2.55)	N(1,0.51)	N(1,0.51)	N(20, 5.1)	N(5,2.55)	N(5,2.55)	N(27.5,11.48)	N(5,2.55)	N(5,2.55)	N(10,5.1)	N(5,2.55)
**Boom**	N(5,2.55)	N(5,2.55)	N(5,2.55)	N(1,0.51)	N(1,0.51)	N(20, 5.1)	N(5,2.55)	N(5,2.55)	N(27.5,11.48)	N(5,2.55)	N(5,2.55)	N(15,7.65)	N(5,2.55)
**Doom**	N(5,2.55)	N(5,2.55)	N(5,2.55)	N(1,0.51)	N(1,0.51)	N(20, 5.1)	N(5,2.55)	N(5,2.55)	N(27.5,11.48)	N(5,2.55)	N(5,2.55)	N(10,5.1)	N(5,2.55)
**Quality of life**	N(5,2.55)	N(5,2.55)	N(5,2.55)	N(1,0.51)	N(1,0.51)	N(20, 5.1)	N(5,2.55)	N(5,2.55)	N(27.5,11.48)	N(5,2.55)	N(5,2.55)	N(10,5.1)	N(5,2.55)
**HW: Days per year with esthetic impairment such as taste, smell, etc.[d/a] (aes_hw)**													
**Status quo**	N(5,2.55)	N(5,2.55)	N(5,2.55)	N(55,22.96)	N(75,12.76)	N(20, 5.1)	N(10,5.1)	N(10,5.1)	N(27.5,11.48)	N(5,2.55)	N(5,2.55)	N(10,5.1)	N(5,2.55)
**Boom**	N(5,2.55)	N(5,2.55)	N(5,2.55)	N(55,22.96)	N(75,12.76)	N(20, 5.1)	N(10,5.1)	N(10,5.1)	N(27.5,11.48)	N(5,2.55)	N(5,2.55)	N(15,7.65)	N(5,2.55)
**Doom**	N(5,2.55)	N(5,2.55)	N(5,2.55)	N(55,22.96)	N(75,12.76)	N(20, 5.1)	N(10,5.1)	N(10,5.1)	N(27.5,11.48)	N(5,2.55)	N(5,2.55)	N(10,5.1)	N(5,2.55)
**Quality of life**	N(5,2.55)	N(5,2.55)	N(5,2.55)	N(55,22.96)	N(75,12.76)	N(20, 5.1)	N(10,5.1)	N(10,5.1)	N(27.5,11.48)	N(5,2.55)	N(5,2.55)	N(10,5.1)	N(5,2.55)
**DW: Days per year with hygienic concerns (hygiene indicators) [d/a] (faecal_dw)**													
**Status quo**	N(2.5,1.28)	N(2.5,1.28)	N(2.5,1.28)	U(0,0)	U(0,0)	N(1,0.51)	U(0,0)	U(0,0)	U(0,0)	N(2.5,1.28)	N(2.5,1.28)	N(5,2.55)	N(2.5,1.28)
**Boom**	N(1,0.51)	N(1,0.51)	N(1,0.51)	U(0,0)	U(0,0)	N(1,0.51)	U(0,0)	U(0,0)	U(0,0)	N(2.5,1.28)	N(2.5,1.28)	N(5,2.55)	N(2.5,1.28)

	A1a	A1b	A2	A3	A4	A5	A6	A6*	A7	A8a	A8b	A9	A0
**Doom**	N(2.5,1.28)	N(2.5,1.28)	N(2.5,1.28)	U(0,0)	U(0,0)	N(1,0.51)	U(0,0)	U(0,0)	U(0,0)	N(2.5,1.28)	N(2.5,1.28)	N(5,2.55)	N(2.5,1.28)
**Quality of life**	N(2.5,1.28)	N(2.5,1.28)	N(2.5,1.28)	U(0,0)	U(0,0)	N(1,0.51)	U(0,0)	U(0,0)	U(0,0)	N(2.5,1.28)	N(2.5,1.28)	N(5,2.55)	N(2.5,1.28)
**HW: Days per year with hygienic concerns (hygiene indicators) [d/a] (faecal_hw)**													
**Status quo**	N(2.5,1.28)	N(2.5,1.28)	N(2.5,1.28)	U(0,0)	N(20, 5.1)	N(1,0.51)	N(5,2.55)	N(5,2.55)	U(0,0)	N(2.5,1.28)	N(2.5,1.28)	N(5,2.55)	N(2.5,1.28)
**Boom**	N(1,0.51)	N(1,0.51)	N(1,0.51)	U(0,0)	N(20, 5.1)	N(1,0.51)	N(5,2.55)	N(5,2.55)	U(0,0)	N(2.5,1.28)	N(2.5,1.28)	N(5,2.55)	N(2.5,1.28)
**Doom**	N(2.5,1.28)	N(2.5,1.28)	N(2.5,1.28)	U(0,0)	N(20, 5.1)	N(1,0.51)	N(5,2.55)	N(5,2.55)	U(0,0)	N(2.5,1.28)	N(2.5,1.28)	N(5,2.55)	N(2.5,1.28)
**Quality of life**	N(2.5,1.28)	N(2.5,1.28)	N(2.5,1.28)	U(0,0)	N(20, 5.1)	N(1,0.51)	N(5,2.55)	N(5,2.55)	U(0,0)	N(2.5,1.28)	N(2.5,1.28)	N(5,2.55)	N(2.5,1.28)
**DW: Changes in total cell count as indicator of bacterial regrowth [log units] (cells_dw)**													
**Status quo**	U(0,0)	U(0,0)	U(0,0)	N(0.15,0.08)	N(-0.5,0.26)	N(-1.5,0.26)	N(0.14,0.07)	N(0.14,0.07)	N(0.34,0.07)	N(0.1,0.05)	N(0.1,0.05)	N(0.15,0.08)	N(0.1,0.05)
**Boom**	N(0.1,0.05)	N(0.1,0.05)	N(0.1,0.05)	N(0.15,0.08)	N(-0.85,0.59)	N(-1.5,0.26)	N(0.14,0.07)	N(0.14,0.07)	N(0.34,0.07)	N(0.15,0.08)	N(0.15,0.08)	N(0.15,0.08)	N(0.15,0.08)
**Doom**	N(0.1,0.05)	N(0.1,0.05)	U(0,0)	N(0.15,0.08)	N(-0.5,0.26)	N(-1.5,0.26)	N(0.14,0.07)	N(0.14,0.07)	N(0.34,0.07)	N(0.1,0.05)	N(0.1,0.05)	N(0.15,0.08)	N(0.1,0.05)
**Quality of life**	U(0,0)	U(0,0)	U(0,0)	N(0.15,0.08)	N(-0.5,0.26)	N(-1.5,0.26)	N(0.14,0.07)	N(0.14,0.07)	N(0.34,0.07)	N(0.1,0.05)	N(0.1,0.05)	N(0.15,0.08)	N(0.1,0.05)
**HW: Changes in total cell count as indicator of bacterial regrowth [log units] (cells_hw)**													
**Status quo**	U(0,0)	U(0,0)	N(0.1,0.05)	N(0.39,0.05)	N(0.35,0.18)	N(-1.5,0.26)	N(0.24,0.03)	N(0.24,0.03)	N(0.34,0.07)	N(0.1,0.05)	N(0.1,0.05)	N(0.15,0.08)	N(0.1,0.05)
**Boom**	N(0.1,0.05)	N(0.1,0.05)	N(0.1,0.05)	N(0.39,0.05)	N(-0.65,0.69)	N(-1.5,0.26)	N(0.23,0.03)	N(0.23,0.03)	N(0.34,0.07)	N(0.15,0.08)	N(0.15,0.08)	N(0.15,0.08)	N(0.15,0.08)
**Doom**	N(0.1,0.05)	U(0,0)	N(0.1,0.05)	N(0.39,0.05)	N(0.35,0.18)	N(-1.5,0.26)	N(0.24,0.03)	N(0.24,0.03)	N(0.34,0.07)	N(0.1,0.05)	N(0.1,0.05)	N(0.15,0.08)	N(0.1,0.05)
**Quality of life**	U(0,0)	U(0,0)	N(0.1,0.05)	N(0.39,0.05)	N(0.35,0.18)	N(-1.5,0.26)	N(0.24,0.03)	N(0.24,0.03)	N(0.34,0.07)	N(0.1,0.05)	N(0.1,0.05)	N(0.15,0.08)	N(0.1,0.05)
**DW and HW: Inorganic substances (indicator: nitrate concentration) [mg/L] (no3_dw, no3_hw)**													
**Status quo**	U(0,20)	U(0,20)	U(0,20)	U(0,20)	U(0,20)	U(0,20)	U(0,20)	U(0,20)	U(0,20)	U(0,20)	U(0,20)	U(0,20)	U(0,20)
**Boom**	U(0,20)	U(0,20)	U(0,20)	U(0,20)	U(0,20)	U(0,20)	U(0,20)	U(0,20)	U(0,20)	U(0,20)	U(0,20)	U(0,20)	U(0,20)
**Doom**	U(0,20)	U(0,20)	U(0,20)	U(0,20)	U(0,20)	U(0,20)	U(0,20)	U(0,20)	U(0,20)	U(0,20)	U(0,20)	U(0,20)	U(0,20)
**Quality of life**	U(0,20)	U(0,20)	U(0,20)	U(0,20)	U(0,20)	U(0,20)	U(0,20)	U(0,20)	U(0,20)	U(0,20)	U(0,20)	U(0,20)	U(0,20)
**DW and HW: Pesticides (sum of pesticide concentration) [µg/L] (pest_dw, pest_hw)**													
**Status quo**	U(0,0.02)	U(0,0.02)	U(0,0.02)	U(0,0.02)	U(0,0.02)	U(0,0.02)	U(0,0.02)	U(0,20)	U(0,0.02)	U(0,0.02)	U(0,0.02)	U(0,0.02)	U(0,20)
**Boom**	U(0,0.02)	U(0,0.02)	U(0,0.02)	U(0,0.02)	U(0,0.02)	U(0,0.02)	U(0,0.02)	U(0,20)	U(0,0.02)	U(0,0.02)	U(0,0.02)	U(0,0.02)	U(0,20)
**Doom**	U(0,0.02)	U(0,0.02)	U(0,0.02)	U(0,0.02)	U(0,0.02)	U(0,0.02)	U(0,0.02)	U(0,20)	U(0,0.02)	U(0,0.02)	U(0,0.02)	U(0,0.02)	U(0,20)

	**A1a**	**A1b**	**A2**	**A3**	**A4**	**A5**	**A6**	**A6***	**A7**	**A8a**	**A8b**	**A9**	**A0**
**Quality of life**	U(0,0.02)	U(0,0.02)	U(0,0.02)	U(0,0.02)	U(0,0.02)	U(0,0.02)	U(0,0.02)	U(0,20)	U(0,0.02)	U(0,0.02)	U(0,0.02)	U(0,0.02)	U(0,20)
**DW and HW: Micropollutants (indicator: benzotriazole) [ng/L] (bta_dw, bta_hw)**													
**Status quo**	U(0,150)	U(0,150)	U(0,150)	U(0,150)	U(0,150)	U(0,150)	U(0,150)	U(0,150)	U(0,150)	U(0,150)	U(0,150)	U(0,150)	U(0,150)
**Boom**	U(0,150)	U(0,150)	U(0,150)	U(0,150)	U(0,150)	U(0,150)	U(0,150)	U(0,150)	U(0,150)	U(0,150)	U(0,150)	U(0,150)	U(0,150)
**Doom**	U(0,150)	U(0,150)	U(0,150)	U(0,150)	U(0,150)	U(0,150)	U(0,150)	U(0,150)	U(0,150)	U(0,150)	U(0,150)	U(0,150)	U(0,150)
**Quality of life**	U(0,150)	U(0,150)	U(0,150)	U(0,150)	U(0,150)	U(0,150)	U(0,150)	U(0,150)	U(0,150)	U(0,150)	U(0,150)	U(0,150)	U(0,150)
**Score of the EFQM excellence model (European Foundation for Quality Management) [%] (efqm)**													
**Status quo**	N(68, 6.63)	N(72,6.63)	N(69,4.59)	N(37,5.61)	N(39,7.65)	N(33,5.61)	N(65,2.55)	N(65,2.55)	N(62,5.1)	N(63,2.55)	N(63,2.55)	N(46,8.16)	N(45,12.76)
**Boom**	N(72,4.59)	N(72,6.63)	N(71,4.59)	N(39,5.61)	N(41,7.65)	N(35,5.61)	N(69,2.55))	N(69,2.55))	N(60,5.1)	N(63,2.55)	N(63,2.55)	N(48,8.16)	N(45,12.76)
**Doom**	N(67, 6.12)	N(70,6.63)	N(66,5.1)	N(35,5.61)	N(37,7.65)	N(31,5.61)	N(63,2.55)	N(63,2.55)	N(64,5.1)	N(65,2.55)	N(65,2.55)	N(42,8.16)	N(45,12.76)
**Quality of life**	N(72,4.59)	N(72,6.63)	N(71,4.59)	N(37,5.61)	N(39,7.65)	N(33,5.61)	N(65,2.55)	N(65,2.55)	N(62,5.1)	N(63,2.55)	N(63,2.55)	N(46,8.16)	N(45,12.76)
**Degree (percent) of codetermination [%] (voice)**													
**Status quo**	N(20,10.2)	N(40,10.2)	N(50,4.51)	N(80,10.2)	N(70,15.31)	N(80,10.2)	N(60,10.2)	N(60,10.2)	N(75,12.76)	N(70,10.2)	N(70,10.2)	N(80,10.2)	N(50,5.1)
**Boom**	N(20,10.2)	N(40,10.2)	N(50,4.51)	N(80,10.2)	N(70,15.31)	N(80,10.2)	N(60,10.2)	N(60,10.2)	N(75,12.76)	N(70,10.2)	N(70,10.2)	N(80,10.2)	N(50,5.1)
**Doom**	N(20,10.2)	N(40,10.2)	N(50,4.51)	N(80,10.2)	N(70,15.31)	N(80,10.2)	N(60,10.2)	N(60,10.2)	N(75,12.76)	N(70,10.2)	N(70,10.2)	N(80,10.2)	N(50,5.1)
**Quality of life**	N(20, 10.2)	N(40, 10.2)	N(50, 4.51)	N(80, 10.2)	N(70, 15.31)	N(80, 10.2)	N(60, 10.2)	N(60, 10.2)	N(75, 12.76)	N(70, 10.2)	N(70, 10.2)	N(80, 10.2)	N(50, 5.1)
**% of water coming from the Mönchaltorfer Aa region [%] (auton)**													
**Status quo**	U(55.20, 55.20)	U(55.20, 55.20)	U(55.20, 55.20)	U(80.32, 80.32)	U(55.46, 55.46)	U(100, 100)	U(90,90)	U(72.44, 72.44)	U(89.33, 89.33)	U(55.46, 55.46)	U(55.46, 55.46)	U(55.46, 55.46)	U(55.46, 55.46)
**Boom**	U(5.25, 5.25)	U(5.25, 5.25)	U(5.25, 5.25)	U(79.05, 79.05)	U(5.28, 5.28)	U(100, 100)	U(90,90)	U(11.67, 11.67)	U(14.47, 14.47)	U(5.28, 5.28)	U(5.28, 5.28)	U(55.46, 55.46)	U(5.28, 5.28)
**Doom**	U(57.58, 57.58)	U(57.58, 57.58)	U(57.58, 57.58)	U(70.01, 70.01)	U(57.85, 57.85)	U(100, 100)	U(90,90)	U(77.52, 77.52)	U(93.55, 93.55)	U(57.85, 57.85)	U(57.85, 57.85)	U(55.46, 55.46)	U(57.85, 57.85)
**Quality of life**	U(48.17, 48.17)	U(48.17, 48.17)	U(48.17, 48.17)	U(81.08, 81.08)	U(48.40, 48.40)	U(100, 100)	U(90,90)	U(64.40, 64.40)	U(81.17, 81.17)	U(48.40, 48.40)	U(48.40, 48.40)	U(48.40, 48.40)	U(48.40, 48.40)
**Necessary time investment for operation and maintenance by user [h/(inh.*a)] (time)**													
**Status quo**	U(0,0)	U(0,0)	U(0.36, 0.36)	U(1.69, 1.69)	U(5,5)	U(8.04, 8.04)	U(0.36, 0.36)	U(0.36, 0.36)	U(1.69, 1.69)	U(0,0)	U(0,0)	U(0,0)	U(0,0)
**Boom**	U(0,0)	U(0,0)	U(0.12, 0.12)	U(0.9, 0.9)	U(0,0)	U(4.94, 4.94)	U(0.12, 0.12)	U(0.12, 0.12)	U(0.9, 0.9)	U(0,0)	U(0,0)	U(0,0)	U(0,0)
**Doom**	U(0,0)	U(0,0)	U(0.36, 0.36)	U(1.33, 1.33)	U(5,5)	U(9.65, 9.65)	U(0.36, 0.36)	U(0.36, 0.36)	U(1.69, 1.69)	U(0,0)	U(0,0)	U(0,0)	U(0,0)

	A1a	A1b	A2	A3	A4	A5	A6	A6*	A7	A8a	A8b	A9	A0
**Quality of life**	U(0,0)	U(0,0)	U(0.3326, 0.3326)	U(1.4917, 1.4917)	U(4.9064, 4.9064)	U(6.9569, 6.9569)	U(0.3326, 0.3326)	U(0.3326, 0.3326)	U(1.595, 1.595)	U(0,0)	U(0,0)	U(0,0)	U(0,0)
**Additional area demand on private property per end user [m²/inh] (area)**													
**Status quo**	U(0,0)	U(0,0)	U(0,0)	U(7.35, 7.35)	U(0.25, 0.25)	U(5.63, 5.63)	U(6.78, 6.78)	U(6.78, 6.78)	U(7.09, 7.09)	U(0,0)	U(0,0)	U(0,0)	U(0,0)
**Boom**	U(0,0)	U(0,0)	U(0.57, 0.57)	U(4.31, 4.31)	U(2.59, 2.59)	U(3.27, 3.27)	U(2.50, 2.50)	U(2.50, 2.50)	U(3.52, 3.52)	U(0,0)	U(0,0)	U(0.57, 0.57)	U(0,0)
**Doom**	U(0,0)	U(0,0)	U(0,0)	U(7.35, 7.35)	U(0.25, 0.25)	U(5.63, 5.63)	U(6.78, 6.78)	U(6.78, 6.78)	U(7.09, 7.09)	U(0,0)	U(0,0)	U(0, 0)	U(0,0)
**Quality of life**	U(0,0)	U(0,0)	U(0.35, 0.35)	U(7.12, 7.12)	U(0.25, 0.25)	U(5.40, 5.40)	U(6.52, 6.52)	U(6.52, 6.52)	U(6.74, 6.74)	U(0,0)	U(0,0)	U(0.35, 0.35)	U(0,0)
**Number of infrastructure sectors that collaborate in planning and construction [-] (collab)**													
**Status quo**	U(6,6)	U(6,6)	U(6,6)	U(1,1)	U(1,1)	U(2,2)	U(6,6)	U(6,6)	U(6,6)	U(2,2)	U(2,2)	U(1,1)	U(2,2)
**Boom**	U(6,6)	U(6,6)	U(6,6)	U(1,1)	U(1,1)	U(2,2)	U(6,6)	U(6,6)	U(6,6)	U(2,2)	U(2,2)	U(1,1)	U(2,2)
**Doom**	U(6,6)	U(6,6)	U(6,6)	U(1,1)	U(1,1)	U(2,2)	U(6,6)	U(6,6)	U(6,6)	U(2,2)	U(2,2)	U(1,1)	U(2,2)
**Quality of life**	U(6,6)	U(6,6)	U(6,6)	U(1,1)	U(1,1)	U(2,2)	U(6,6)	U(6,6)	U(6,6)	U(2,2)	U(2,2)	U(1,1)	U(2,2)
**Annual cost per person in% of the mean taxable income [%] (costcap)**													
**Status quo**	LN(-5.1776, 0.1232)	LN(-5.1776, 0.1232)	TN(0.0039, 0.0006)[0.002,0.007]	LN(-4.2529, 0.2835)	LN(-5.6495, 0.1676)	LN(-5.0688, 0.3677)	TN(0.0039, 0.0006)[0.002, 0.006]	TN(0.0039, 0.0006)[0.002, 0.007]	LN(-4.7923, 0.2947)	LN(-5.5707, 0.1603)	LN(-5.5707, 0.1603)	β(25.88, 8599.462)	LN(-5.73,0.17)
**Boom**	U(0.0346, 0.0565)	U(0.0346, 0.0565)	U(0.02, 0.04)	U(0.0018, 0.0225)	U(0.0015, 0.021)	U(0.0007, 0.0052)	U(0.016, 0.0365)	U(0.0162, 0.0437)	β(10.9985 5798.49	U(0.0101, 0.0432)	U(0.0085, 0.0359)	U(0.0147, 0.0327)	U(0.0187,0.0417)
**Doom**	LN(-4.3689, 0.1219)	LN(-4.3689, 0.1219)	LN(-4.745, 0.1434)	TN(0.035, 0.0092)[0,0.08]	LN(-4.8506, 0.1726)	LN(-4.2149, 0.3446)	TN(0.0087, 0.0013)[0.004, 0.014]	TN(0.0088, 0.0013)[0.004, 0.016]	TN(0.02, 0.0127) [0,0.2]	TN(0.0085, 0.0014)[0.004, 0.014]	TN(0.0085, 0.0014)[0.004, 0.014]	TN(0.0066, 0.0012)[0.002, 0.012]	β(37.7,5054.2)
**Quality of life**	U(0.0088, 0.0147)	U(0.0088, 0.0147)	U(0.0042, 0.0091)	β(12.4288, 1453.01)	U(0.004, 0.009)	LN(-5.6628, 0.3674)	U(0.0043, 0.0093)	U(0.0041, 0.0091)	LN(-5.3033, 0.2926)	U(0.003, 0.0102)	U(0.003, 0.0102)	U(0.0034, 0.0075)	U(0.0042,0.0093)
**Mean annual (linear) increase of costs [%/a] (costchange)**													
**Status quo**	N(0.0062, 0.0003)	N(0.0062, 0.0003)	N(0.0043, 0.0002)	N(0.0043, 0.0002)	N(0.0038, 0.0002)	N(0.0074, 0.0004)	N(0.0043, 0.0002)	N(0.0043, 0.0002)	N(0.0094, 0.0005)	N(0.0042, 0.0002)	N(0.0042, 0.0002)	N(0.0032, 0.0001)	N(0.0036,0.0002)
**Boom**	N(0.0216, 0.017)	N(0.0216, 0.017)	N(0.0138, 0.009)	N(0.0297, 0.0138)	N(0.0242, 0.0112)	N(0.0042, 0.002)	N(0.0154, 0.0085)	N(0.0155, 0.0084)	N(0.0094, 0.0005)	N(0.0136, 0.0093)	N(0.012, 0.0076)	N(0.0128, 0.0086)	N(0.0153,0.0114)
**Doom**	N(0.0095, 0.0018)	N(0.0095, 0.0018)	N(0.0066, 0.0012)	N(0.0264, 0.0047)	N(0.0059, 0.0011)	N(0.0118, 0.0021)	N(0.0065, 0.0012)	N(0.0065, 0.0012)	N(0.0151, 0.0027)	N(0.0063, 0.0012)	N(0.0063, 0.0012)	N(0.0049, 0.0009)	N(0.0055,0.001)
**Quality of life**	N(0.0096, 0.0014)	N(0.0096, 0.0014)	N(0.0061, 0.0006)	N(0.013, 0.0031)	N(0.0059, 0.0007)	N(0.0057, 0.0014)	N(0.006, 0.0005)	N(0.006, 0.0006)	N(0.008, 0.0019)	N(0.0059, 0.0006)	N(0.0059, 0.0006)	N(0.0049, 0.0005)	N(0.0058,0.0008)

Part C – Stakeholder preferences

Tab. C.1. Elicited weights from face-to-face interviews with ten stakeholders Unfortunately, no permission could be obtained to reprint the original table here. Therefore please be referred to supporting information table SI4.1 in [5].

Tab. C.2. Value function preferences Unfortunately, no permission could be obtained to reprint the original table here. Therefore please be referred to supporting information table SI4.4 in [5].

Tab. C.3. Stated acceptance thresholds and potential preference interactions Unfortunately, no permission could be obtained to reprint the original table here. Therefore please be referred to supporting information table SI4.6 in [5].

Part D – Assumptions for uncertainty simulation

Text D.1. Supplementary modeling assumptions for evaluation layout L5

Hierarchical value (aggregation) function

$$V(A) = \alpha_k \cdot V_{add}(A) + (1 - \alpha_k) \cdot V_{CD} \; ; \; 0 \leq \alpha_k \leq 1$$

The mixture of the weighted arithmetic and geometric mean model (additive and Cobb-Douglas model) allows to combine a limited compensability of bad outcomes on one objective by good outcomes on another while avoiding that the overall value is zero due to a zero value on one of its components (lower-level values). The mixture parameter α_k for aggregation at node k is sampled from a uniform distribution on [0,1], k~Unif(0,1). α = 1 stands for full additivity, α = 0 for pure Cobb-Douglas aggregation.

Single-attribute value functions

The distribution of the shape parameter of the marginal value functions depends on the available preference information.

a) *Supporting points elicited in detail*: an exponential function was fitted to the elicited intervals, such that the uncertainty of the curvature parameter c_j follows a normal distribution $N(\mu_j \; ½ \; \sigma_j)$.
b) *Rough information about shape*: the exponential function parameter c_j *~Unif[min, max]; where Unif[0,10]* is used if concave, *Unif[-10, 0]* if convex, and *Unif[-0.4,0.4]* if approximately linear.
c) *Not elicited:* exponential function with c_j ~ *Unif[-10,10]*

Weights

Weights are independently sampled within each (sub-) branch of the objectives hierarchy from a truncated normal distribution w_i~TN(μ_i, σ_i) truncated at [0,1] and normalized to 1 as required by the additive and Cobb-Douglas model.

Acceptance thresholds, individual adjustments

Not meeting the acceptance thresholds leads to complete elimination of the alternative (overall value = 0). For further, minor adjustments see [5]

Part E – Additional results

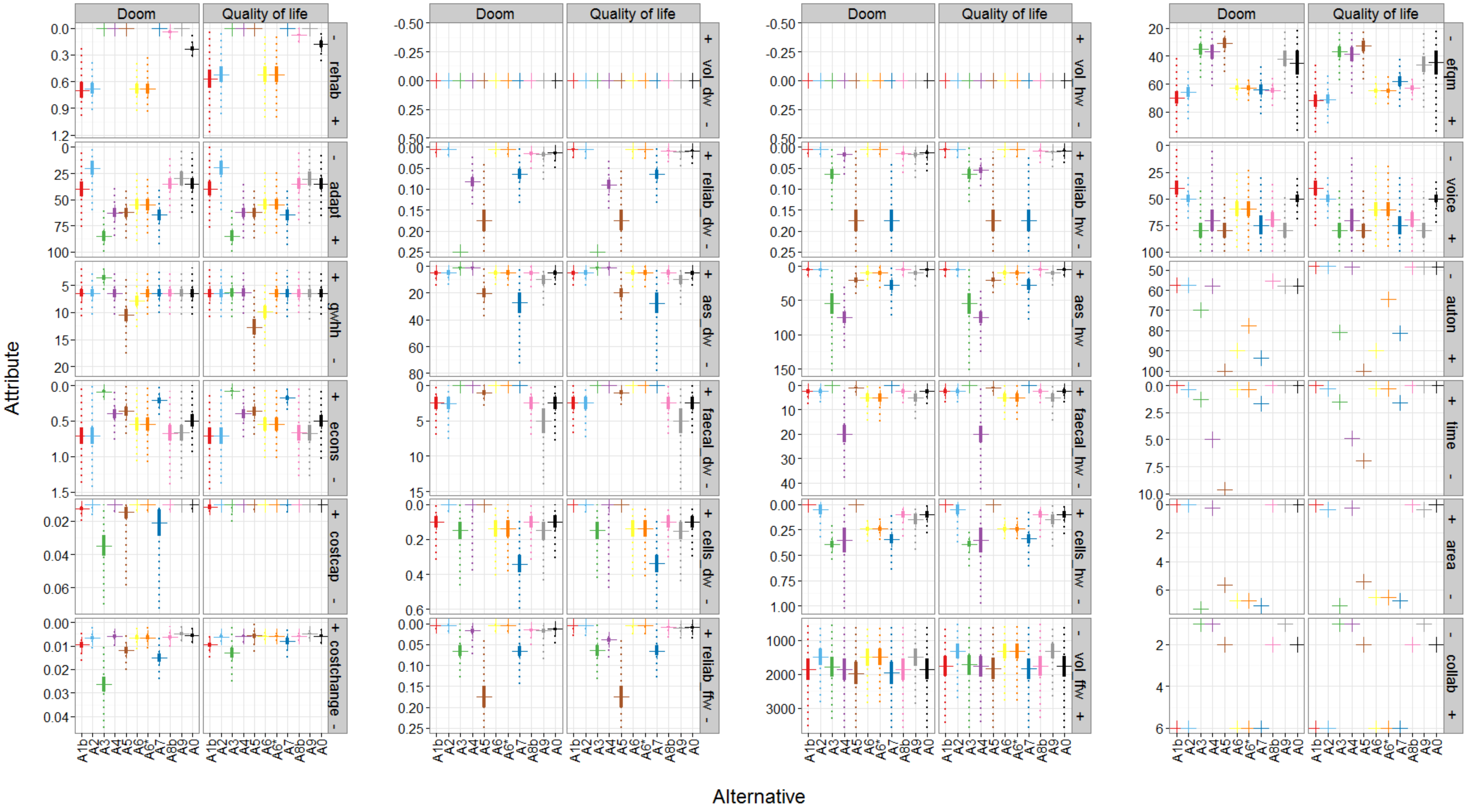

Fig. E.1. Attribute outcomes in the Doom and Quality of life scenarios for the alternatives. Distributional assumptions are given in Tab. B.4, for interpretation see Fig 3 in main text.

Tab. E.1. Absolute mean weights as used in evaluation model layouts L1-L5. The evaluation model layouts L1-L5 are specified in Section 2.5 and Tab. 2, main text. The absolute weights in hierarchical aggregation are determined by multiplying the relative weights downwards along the branches of the objectives hierarchy [21]. Objectives receiving higher weights are mapped with darker grey shading. SH – stakeholder, highest-level objectives: IE – intergenerational equity, RG – resources and groundwater protection, WS – water supply, SA – social acceptance, CO – costs, abbreviations of attributes (column to the far left) see Tab. B.1.

	**L1**	**L2**	**L3-L5: Individual stakeholder weights**									
	**Equal weights (bottom-up)**	**Equal weights with hierarchy (top-down)**	**SH1**	**SH2**	**SH3**	**SH4**	**SH5**	**SH6**	**SH7**	**SH8**	**SH9**	**SH10**
**IE_rehab**	0.033	0.100	0.108	0.130	0.030	0.158	0.163	0.136	0.074	0.161	0.131	0.000
**IE_flex**	0.033	0.100	0.064	0.064	0.087	0.126	0.130	0.095	0.093	0.040	0.065	0.000
**RG_gwhh**	0.033	0.100	0.161	0.159	0.180	0.172	0.040	0.111	0.199	0.208	0.123	0.436
**RG_energ**	0.033	0.100	0.079	0.062	0.063	0.060	0.058	0.055	0.049	0.103	0.024	0.000
**WS_dw.quant**	0.033	0.022	0.029	0.032	0.035	0.039	0.032	0.057	0.027	0.010	0.028	0.030
**WS_dw.reliab**	0.033	0.022	0.058	0.037	0.050	0.057	0.034	0.088	0.033	0.055	0.037	0.061
**WS_dw.q.aest**	0.033	0.007	0.015	0.018	0.018	0.026	0.013	0.035	0.010	0.019	0.019	0.022
**WS_dw.q.hyg**	0.033	0.004	0.014	0.011	0.020	0.014	0.007	0.029	0.011	0.105	0.016	0.022
**WS_dw.q.cell**	0.033	0.004	0.007	0.010	0.020	0.014	0.006	0.006	0.006	0.020	0.008	0.000
**WS_dw.q.no3**	0.033	0.002	0.004	0.003	0.010	0.010	0.004	0.017	0.002	0.020	0.000	0.000
**WS_dw.q.pest**	0.033	0.002	0.004	0.005	0.010	0.010	0.004	0.017	0.005	0.010	0.000	0.006
**WS_dw.q.bta**	0.033	0.002	0.004	0.004	0.008	0.008	0.004	0.017	0.005	0.014	0.005	0.005
**WS_hw.quant**	0.033	0.022	0.034	0.026	0.020	0.009	0.056	0.008	0.026	0.024	0.022	0.046
**WS_hw.reliab**	0.033	0.022	0.049	0.040	0.028	0.013	0.063	0.020	0.037	0.054	0.030	0.052
**WS_hw.q.aest**	0.033	0.007	0.007	0.011	0.010	0.004	0.009	0.004	0.010	0.016	0.014	0.025
**WS_hw.q.hyg**	0.033	0.004	0.008	0.004	0.012	0.003	0.001	0.002	0.013	0.021	0.016	0.022
**WS_hw.q.cell**	0.033	0.004	0.003	0.003	0.011	0.003	0.001	0.001	0.004	0.004	0.008	0.000
**WS_hw.q.no3**	0.033	0.002	0.002	0.001	0.006	0.001	0.001	0.001	0.000	0.000	0.000	0.000
**WS_hw.q.pest**	0.033	0.002	0.002	0.003	0.006	0.001	0.001	0.001	0.000	0.000	0.000	0.000
**WS_hw.q.bta**	0.033	0.002	0.002	0.001	0.004	0.001	0.001	0.001	0.000	0.000	0.000	0.000
**WS_lw.reliab**	0.033	0.033	0.063	0.047	0.039	0.078	0.054	0.032	0.048	0.000	0.030	0.075
**WS_lw.quant**	0.033	0.033	0.044	0.023	0.029	0.065	0.038	0.016	0.041	0.000	0.014	0.025
**SA_efqm**	0.033	0.033	0.000	0.032	0.019	0.003	0.012	0.090	0.038	0.010	0.056	0.000
**SA_voice**	0.033	0.033	0.000	0.021	0.015	0.001	0.011	0.026	0.019	0.002	0.012	0.000
**SA_auton**	0.033	0.033	0.000	0.024	0.013	0.011	0.000	0.000	0.010	0.008	0.012	0.000
**SA_time**	0.033	0.033	0.000	0.014	0.010	0.009	0.012	0.000	0.010	0.000	0.045	0.000
**SA_area**	0.033	0.033	0.000	0.010	0.013	0.007	0.000	0.000	0.010	0.004	0.045	0.000
**SA_collab**	0.033	0.033	0.000	0.026	0.015	0.006	0.015	0.000	0.027	0.004	0.033	0.000
**CO_year**	0.033	0.100	0.134	0.067	0.109	0.040	0.085	0.089	0.130	0.046	0.208	0.052
**CO_incr**	0.033	0.100	0.106	0.113	0.109	0.050	0.143	0.044	0.064	0.046	0.000	0.121
∑	1	1	1	1	1	1	1	1	1	1	1	1

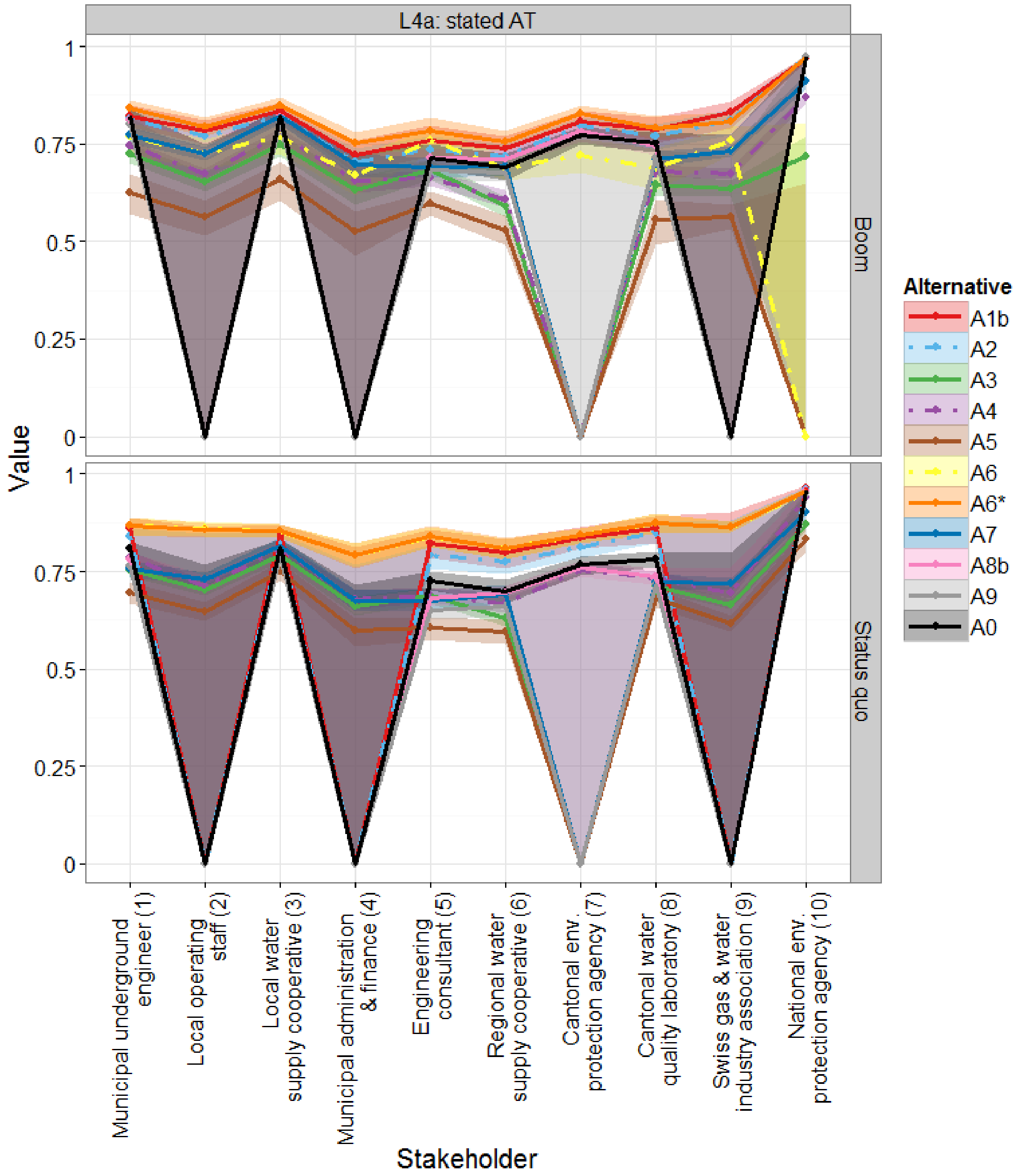

Fig. E.2. Expected values (EV) of alternatives for ten stakeholders. with stated acceptance thresholds AT for certain attributes and two scenarios. Such stated AT, which were defined by some stakeholders, were implemented in the evaluation model layout L4a. An AT renders an alternative unacceptable if the respective attribute were to fall below the stated AT. In this case, the total value (EV) of that alternative receives a value of 0 (see "dips" in Fig. E.2).

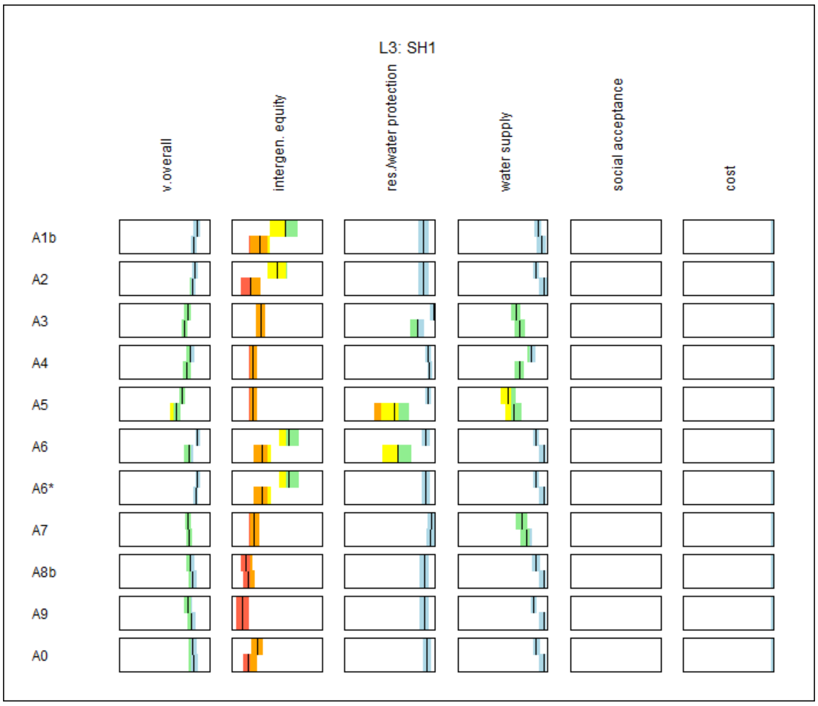

Fig. E.3. Value of alternatives (rows) for stakeholder SH1 in evaluation model layout L3. Overall values (v.overall) and values of the five main objectives (columns) are displayed. Red color represents low values, the left end of the box representing value v=0, the right end v=1 (blue color). The upper half of each box shows the values of the alternative in the Status quo, the lower half in the Boom scenario. Vertical black lines represent the median, colored areas the 5-95% quantile intervals. Boxes for social acceptance are empty because this stakeholder discarded the objective as irrelevant (weight = 0).

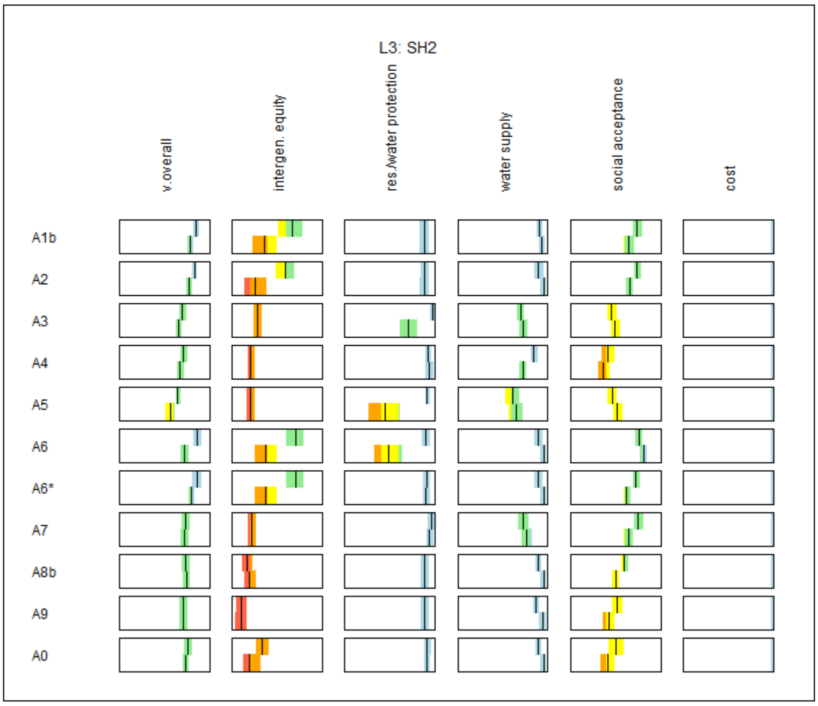

Fig. E.4. Value of alternatives for stakeholder SH2 in evaluation model layout L3. For interpretation see Fig. E.3.

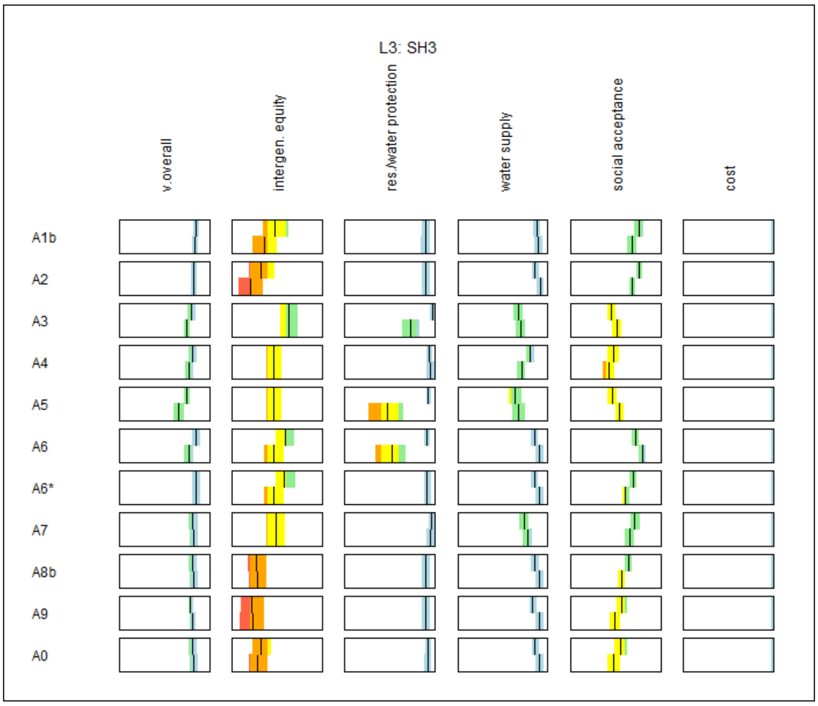

Fig. E.5. Value of alternatives for stakeholder SH3 in evaluation model layout L3. For interpretation see Fig. E.3.

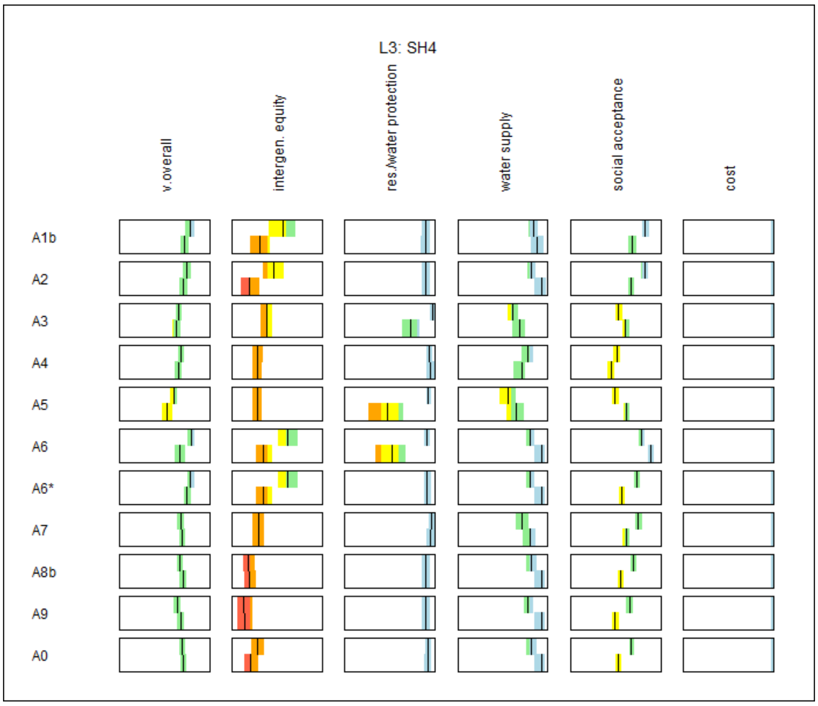

Fig. E.6. Value of alternatives for stakeholder SH4 in evaluation model layout L3. For interpretation see Fig. E.3.

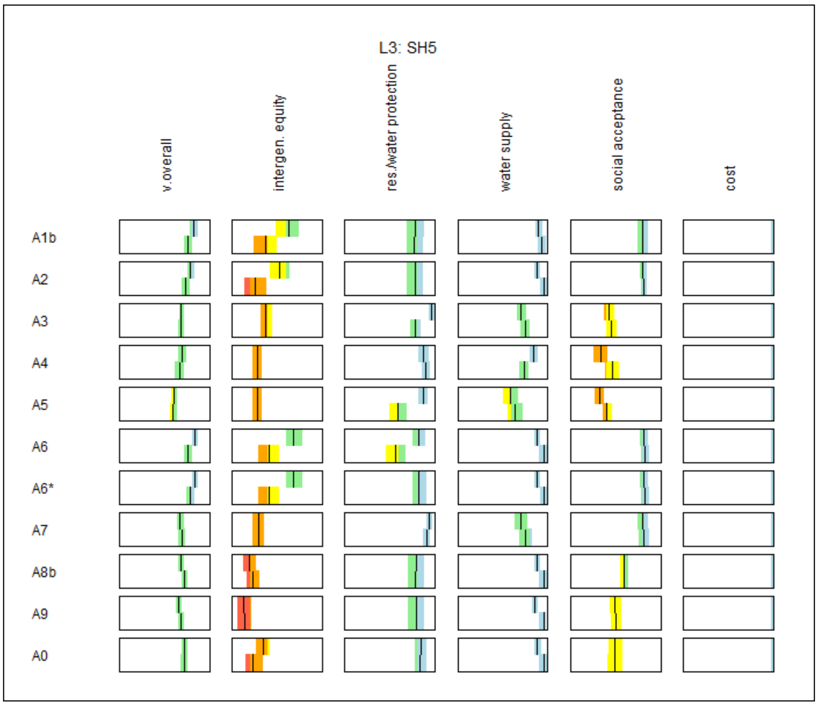

Fig. E.7. Value of alternatives for stakeholder SH5 in evaluation model layout L3. For interpretation see Fig. E.3.

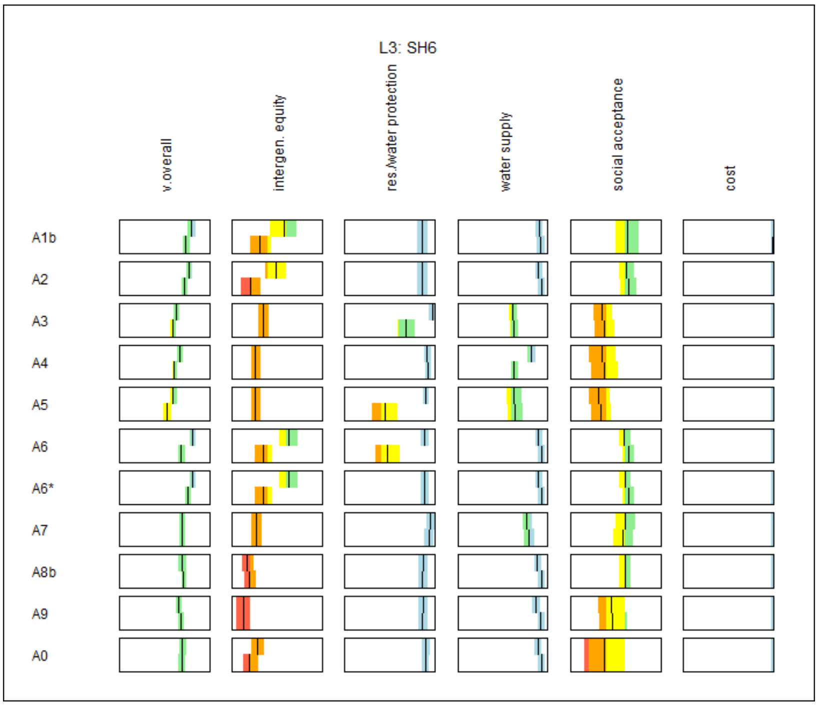

Fig. E.8. Value of alternatives for stakeholder SH6 in evaluation model layout L3. For interpretation see Fig. E.3.

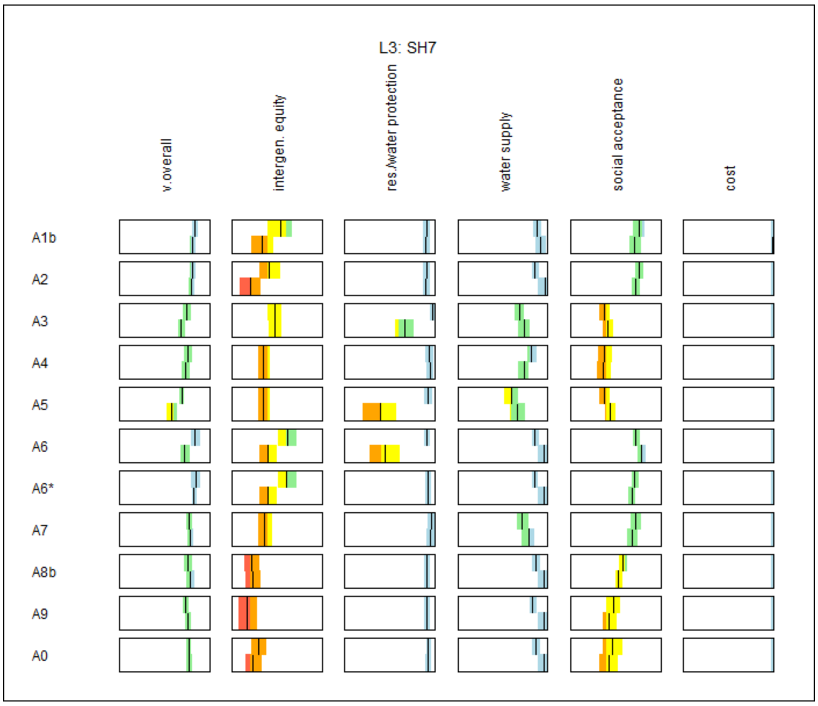

Fig. E.9. Value of alternatives for stakeholder SH7 in evaluation model layout L3. For interpretation see Fig. E.3.

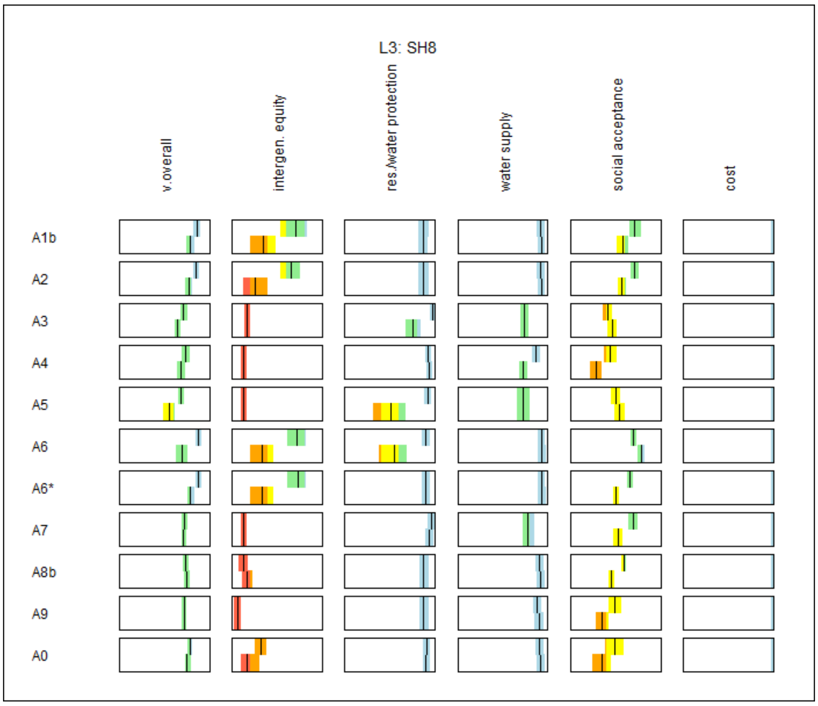

Fig. E.10. Value of alternatives for stakeholder SH8 in evaluation model layout L3. For interpretation see Fig. E.3.

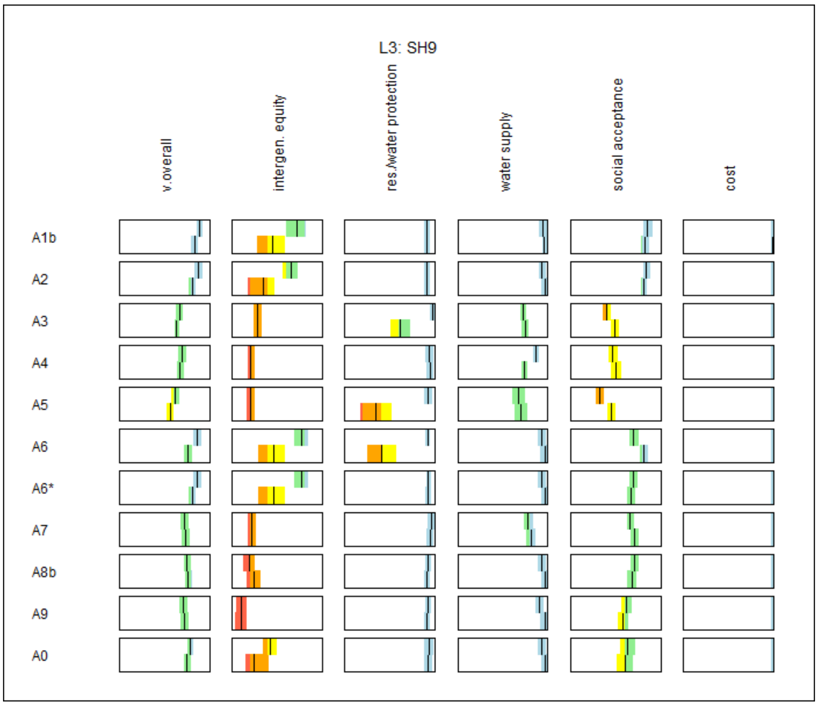

Fig. E.11. Value of alternatives for stakeholder SH9 in evaluation model layout L3. For interpretation see Fig. E.3.

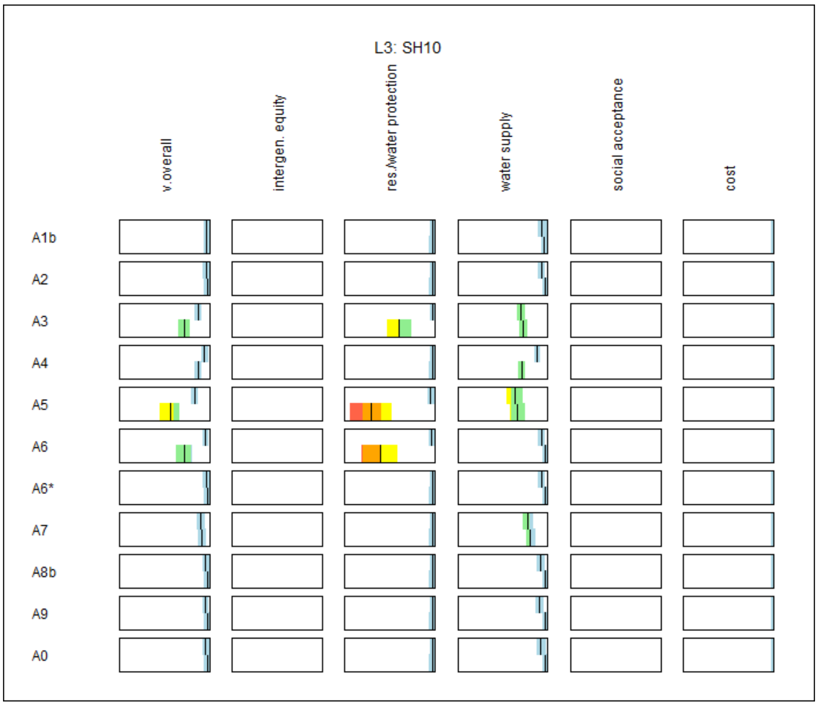

Fig. E.12. Value of alternatives for stakeholder SH10 in evaluation model layout L3. For interpretation see Fig. E.3.

Tab. E.2. Overall value and ranking of alternatives (A1b–A0) for ten stakeholders (SH), two scenarios, and three aggregation model layouts that include the preferences of stakeholders (L3, L4b, L5; Tab. 2). Expected values (mean, μ), standard deviations (σ), rank on expected values (R), and average over expected values (Avg(μ)) are shown. Bold: alternative achieving the highest rank and value; italic: lowest rank and value.

	SH1			SH2			SH3			SH4			SH5			SH6			SH7			SH8			SH9			SH10			aggregated (SH1-10)		
	R	μ	σ	R	μ	σ	R	μ	σ	R	μ	σ	R	μ	σ	R	μ	σ	R	μ	σ	R	μ	σ	R	μ	σ	R	μ	σ	R	μ	σ
**L3 – Boom scenario**																																	
**A1b**	2	0.822	0.02	2	0.784	0.02	2	0.834	0.01	2	0.723	0.02	2	0.759	0.02	2	0.739	0.02	2	0.809	0.02	2	0.786	0.02	1	0.831	0.02	6	0.968	0.01	2	0.806	0.07
**A2**	4	0.814	0.01	3	0.770	0.02	3	0.823	0.01	4	0.705	0.02	4	0.734	0.02	3	0.719	0.02	3	0.797	0.01	3	0.769	0.02	2	0.810	0.02	1	0.974	0.01	3	0.792	0.07
**A3**	10	0.725	0.02	10	0.654	0.01	10	0.747	0.02	10	0.630	0.02	8	0.684	0.01	10	0.589	0.02	10	0.688	0.02	10	0.646	0.02	10	0.636	0.01	10	0.717	0.03	10	0.672	0.05
**A4**	9	0.745	0.01	9	0.674	0.01	8	0.774	0.01	9	0.653	0.02	10	0.663	0.01	9	0.609	0.01	8	0.733	0.01	9	0.682	0.01	9	0.672	0.01	8	0.870	0.01	9	0.707	0.07
**A5**	11	0.624	0.03	11	0.563	0.03	11	0.658	0.03	11	0.526	0.03	11	0.598	0.02	11	0.528	0.02	11	0.581	0.03	11	0.554	0.03	11	0.564	0.02	11	0.558	0.06	11	0.575	0.04
**A6**	8	0.767	0.03	6	0.725	0.02	9	0.769	0.03	8	0.669	0.03	3	0.759	0.02	7	0.687	0.02	9	0.722	0.03	8	0.688	0.03	5	0.758	0.02	9	0.719	0.05	8	0.726	0.03
**A6***	1	0.843	0.01	1	0.794	0.01	1	0.850	0.01	1	0.750	0.02	1	0.784	0.02	1	0.758	0.02	1	0.829	0.01	1	0.791	0.02	3	0.806	0.02	4	0.973	0.01	1	0.818	0.06
**A7**	7	0.774	0.01	7	0.724	0.01	5	0.821	0.01	6	0.698	0.02	7	0.690	0.02	5	0.694	0.01	4	0.784	0.01	7	0.712	0.01	7	0.732	0.01	7	0.910	0.01	6	0.754	0.07
**A8b**	5	0.812	0.01	4	0.741	0.01	4	0.822	0.01	5	0.702	0.02	6	0.715	0.01	4	0.712	0.01	5	0.782	0.01	5	0.745	0.01	4	0.765	0.01	2	0.973	0.01	4	0.777	0.08
**A9**	6	0.799	0.01	8	0.711	0.01	7	0.808	0.01	7	0.681	0.02	9	0.683	0.02	8	0.679	0.02	7	0.759	0.01	6	0.715	0.01	8	0.717	0.01	5	0.972	0.01	7	0.752	0.09
**A0**	3	0.820	0.01	5	0.736	0.01	6	0.820	0.01	3	0.708	0.02	5	0.717	0.02	6	0.691	0.02	6	0.774	0.01	4	0.752	0.02	6	0.754	0.02	3	0.973	0.01	5	0.775	0.08
**L3 – Status quo scenario**																																	
**A1b**	3	0.859	0.02	3	0.848	0.02	3	0.848	0.01	3	0.783	0.03	3	0.822	0.02	3	0.797	0.02	3	0.838	0.02	3	0.859	0.02	1	0.888	0.01	1	0.963	0.00	3	0.851	0.05
**A2**	4	0.839	0.01	4	0.831	0.01	4	0.826	0.01	4	0.746	0.02	4	0.790	0.02	4	0.773	0.02	4	0.812	0.01	4	0.850	0.02	2	0.870	0.01	2	0.959	0.00	4	0.830	0.06
**A3**	10	0.753	0.01	10	0.701	0.01	9	0.792	0.01	9	0.661	0.01	7	0.687	0.01	10	0.630	0.01	9	0.747	0.01	10	0.714	0.01	10	0.666	0.01	10	0.872	0.01	10	0.722	0.07
**A4**	6	0.786	0.01	8	0.712	0.01	7	0.810	0.01	6	0.679	0.01	6	0.689	0.01	8	0.669	0.01	8	0.755	0.01	7	0.734	0.01	9	0.697	0.01	8	0.941	0.01	7	0.747	0.08
**A5**	11	0.696	0.02	11	0.649	0.01	11	0.750	0.01	11	0.599	0.02	11	0.604	0.02	11	0.595	0.02	11	0.690	0.01	11	0.682	0.01	11	0.617	0.01	11	0.834	0.02	11	0.672	0.07
**A6**	2	0.866	0.01	1	0.859	0.01	1	0.855	0.01	1	0.791	0.02	2	0.840	0.02	2	0.808	0.02	2	0.842	0.01	2	0.874	0.02	3	0.865	0.01	6	0.955	0.00	2	0.855	0.04
**A6***	1	0.867	0.01	2	0.856	0.01	2	0.853	0.01	2	0.791	0.02	1	0.840	0.02	1	0.809	0.02	1	0.843	0.01	1	0.874	0.02	4	0.864	0.01	3	0.959	0.00	1	0.856	0.04
**A7**	9	0.758	0.01	6	0.730	0.01	5	0.816	0.01	7	0.676	0.02	9	0.675	0.02	7	0.692	0.01	5	0.773	0.01	8	0.722	0.01	7	0.718	0.01	9	0.900	0.01	8	0.746	0.07
**A8b**	7	0.780	0.01	7	0.730	0.01	8	0.810	0.01	8	0.665	0.02	8	0.682	0.01	6	0.694	0.01	7	0.761	0.01	6	0.737	0.01	6	0.750	0.01	5	0.957	0.01	6	0.757	0.08
**A9**	8	0.763	0.01	9	0.702	0.01	10	0.791	0.01	10	0.638	0.02	10	0.651	0.01	9	0.660	0.02	10	0.733	0.01	9	0.714	0.01	8	0.711	0.01	7	0.950	0.00	9	0.731	0.09
**A0**	5	0.808	0.01	5	0.758	0.01	6	0.814	0.01	5	0.698	0.02	5	0.726	0.01	5	0.699	0.02	6	0.767	0.01	5	0.783	0.01	5	0.784	0.01	4	0.957	0.01	5	0.779	0.07
**L4b – Boom scenario**																																	
**A1b**	2	0.822	0.02	2	0.784	0.02	2	0.834	0.01	2	0.723	0.02	2	0.759	0.02	2	0.739	0.02	2	0.809	0.02	2	0.786	0.02	1	0.831	0.02	6	0.968	0.01	2	0.806	0.07
**A2**	4	0.814	0.01	3	0.770	0.02	3	0.823	0.01	3	0.705	0.02	4	0.734	0.02	3	0.719	0.02	3	0.797	0.01	3	0.769	0.02	2	0.810	0.02	1	0.974	0.01	3	0.792	0.07
**A3**	10	0.725	0.02	9	0.654	0.01	10	0.747	0.02	9	0.630	0.02	8	0.684	0.01	10	0.589	0.02	9	0.016	0.11	10	0.646	0.02	9	0.636	0.01	9	0.660	0.21	10	0.599	0.20
**A4**	9	0.745	0.01	8	0.674	0.01	8	0.774	0.01	8	0.653	0.02	10	0.663	0.01	9	0.609	0.01	10	0.002	0.04	9	0.682	0.01	8	0.672	0.01	8	0.870	0.01	8	0.634	0.22
**A5**	11	0.624	0.03	10	0.563	0.03	11	0.658	0.03	10	0.526	0.03	11	0.598	0.02	11	0.528	0.02	11	0.000	0.00	11	0.554	0.03	10	0.564	0.02	11	0.041	0.16	11	0.466	0.23
**A6**	8	0.767	0.03	4	0.725	0.02	9	0.769	0.03	7	0.669	0.03	3	0.759	0.02	7	0.687	0.02	6	0.722	0.03	8	0.688	0.03	4	0.758	0.02	10	0.132	0.30	7	0.668	0.18
**A6***	1	0.843	0.01	1	0.794	0.01	1	0.850	0.01	1	0.750	0.02	1	0.784	0.02	1	0.758	0.02	1	0.829	0.01	1	0.791	0.02	3	0.806	0.02	4	0.973	0.01	1	0.818	0.06
**A7**	7	0.774	0.01	5	0.724	0.01	5	0.821	0.01	4	0.698	0.02	7	0.690	0.02	5	0.694	0.01	8	0.018	0.12	7	0.712	0.01	7	0.732	0.01	7	0.910	0.01	6	0.677	0.23
**A8b**	5	0.812	0.01	6	0.722	0.12	4	0.822	0.01	6	0.684	0.11	6	0.715	0.01	4	0.712	0.01	4	0.763	0.12	5	0.745	0.01	5	0.746	0.12	2	0.973	0.01	4	0.769	0.08
**A9**	6	0.799	0.01	11	0.353	0.36	7	0.808	0.01	11	0.338	0.34	9	0.683	0.02	8	0.679	0.02	7	0.377	0.38	6	0.715	0.01	11	0.356	0.36	5	0.972	0.01	9	0.608	0.22
**A0**	3	0.820	0.01	7	0.718	0.11	6	0.820	0.01	5	0.691	0.11	5	0.717	0.02	6	0.691	0.02	5	0.756	0.12	4	0.752	0.02	6	0.736	0.12	3	0.973	0.01	5	0.767	0.08
**L4b – Status quo scenario**																																	
**A1b**	3	0.859	0.02	3	0.829	0.13	3	0.848	0.01	3	0.765	0.12	3	0.822	0.02	3	0.797	0.02	3	0.819	0.13	3	0.859	0.02	1	0.868	0.13	1	0.963	0.00	3	0.843	0.05
**A2**	4	0.839	0.01	4	0.811	0.13	4	0.826	0.01	4	0.728	0.12	4	0.790	0.02	4	0.773	0.02	4	0.793	0.13	4	0.850	0.02	4	0.849	0.13	2	0.959	0.00	4	0.822	0.06
**A3**	10	0.753	0.01	9	0.701	0.01	9	0.792	0.01	8	0.661	0.01	7	0.687	0.01	10	0.630	0.01	10	0.016	0.11	10	0.714	0.01	9	0.666	0.01	10	0.872	0.01	9	0.649	0.22
**A4**	6	0.786	0.01	7	0.712	0.01	7	0.810	0.01	6	0.679	0.01	6	0.689	0.01	8	0.669	0.01	7	0.701	0.20	7	0.734	0.01	8	0.697	0.01	8	0.941	0.01	7	0.742	0.08
**A5**	11	0.696	0.02	10	0.649	0.01	11	0.750	0.01	10	0.599	0.02	11	0.604	0.02	11	0.595	0.02	11	0.000	0.00	11	0.682	0.01	10	0.617	0.01	11	0.834	0.02	10	0.603	0.21
**A6**	2	0.866	0.01	1	0.859	0.01	1	0.855	0.01	1	0.791	0.02	2	0.840	0.02	2	0.808	0.02	2	0.842	0.01	2	0.874	0.02	2	0.865	0.01	6	0.955	0.00	2	0.855	0.04
**A6***	1	0.867	0.01	2	0.856	0.01	2	0.853	0.01	2	0.791	0.02	1	0.840	0.02	1	0.809	0.02	1	0.843	0.01	1	0.874	0.02	3	0.864	0.01	3	0.959	0.00	1	0.856	0.04

	**SH1**			**SH2**			**SH3**			**SH4**			**SH5**			**SH6**			**SH7**			**SH8**			**SH9**			**SH10**			**aggregated (SH1-10)**		
	**R**	**μ**	**σ**	**R**	**μ**	**σ**	**R**	**μ**	**σ**	**R**	**μ**	**σ**	**R**	**μ**	**σ**	**R**	**μ**	**σ**	**R**	**μ**	**σ**	**R**	**μ**	**σ**	**R**	**μ**	**σ**	**R**	**μ**	**σ**	**R**	**μ**	**σ**
**A7**	9	0.758	0.01	6	0.730	0.01	5	0.816	0.01	7	0.676	0.02	9	0.675	0.02	7	0.692	0.01	9	0.019	0.12	8	0.722	0.01	7	0.718	0.01	9	0.900	0.01	8	0.671	0.23
**A8b**	7	0.780	0.01	8	0.712	0.12	8	0.810	0.01	9	0.648	0.11	8	0.682	0.01	6	0.694	0.01	6	0.728	0.15	6	0.737	0.01	6	0.731	0.12	5	0.957	0.01	6	0.748	0.08
**A9**	8	0.763	0.01	*11*	*0.350*	*0.35*	10	0.791	0.01	*11*	*0.318*	*0.32*	10	0.651	0.01	9	0.660	0.02	8	0.339	0.37	9	0.714	0.01	*11*	*0.354*	*0.36*	7	0.950	0.00	*11*	*0.589*	*0.22*
**A0**	5	0.808	0.01	5	0.742	0.11	6	0.814	0.01	5	0.684	0.10	5	0.726	0.01	5	0.699	0.02	5	0.739	0.15	5	0.783	0.01	5	0.767	0.11	4	0.957	0.01	5	0.772	0.07
**L5 – Boom scenario**																																	
**A1b**	2	0.753	0.11	2	0.695	0.08	2	0.753	0.09	2	0.675	0.11	2	0.693	0.10	2	0.837	0.04	2	0.873	0.04	2	0.705	0.06	**1**	**0.804**	**0.06**	4	0.965	0.02	2	0.775	0.09
**A2**	4	0.729	0.12	3	0.670	0.08	5	0.727	0.09	3	0.628	0.11	4	0.654	0.10	4	0.797	0.05	3	0.838	0.04	3	0.672	0.07	4	0.767	0.06	**1**	**0.967**	**0.02**	3	0.745	0.10
**A3**	10	0.581	0.09	9	0.418	0.08	10	0.545	0.09	9	0.430	0.10	8	0.487	0.10	*11*	*0.430*	*0.09*	9	0.014	0.00	10	0.402	0.09	9	0.494	0.09	9	0.604	0.10	10	0.440	0.16
**A4**	9	0.581	0.10	8	0.429	0.09	9	0.559	0.09	8	0.433	0.11	*11*	*0.466*	*0.12*	10	0.431	0.09	*10*	*0.000*	*0.00*	9	0.418	0.09	10	0.487	0.09	8	0.805	0.06	9	0.461	0.19
**A5**	*11*	*0.547*	*0.10*	10	0.369	0.08	*11*	*0.518*	*0.08*	10	0.388	0.10	10	0.484	0.12	9	0.459	0.08	*10*	*0.000*	*0.00*	*11*	*0.359*	*0.08*	8	0.524	0.08	*11*	*0.039*	*0.01*	*11*	*0.368*	*0.19*
**A6**	6	0.696	0.11	4	0.634	0.08	7	0.686	0.09	5	0.608	0.10	3	0.687	0.10	3	0.817	0.05	6	0.756	0.07	6	0.605	0.08	3	0.774	0.06	10	0.122	0.02	6	0.639	0.18
**A6***	**1**	**0.773**	**0.11**	**1**	**0.702**	**0.07**	**1**	**0.770**	**0.08**	**1**	**0.691**	**0.10**	**1**	**0.713**	**0.10**	**1**	**0.846**	**0.04**	**1**	**0.885**	**0.04**	**1**	**0.706**	**0.06**	2	0.789	0.05	5	0.964	0.02	**1**	**0.784**	**0.09**
**A7**	7	0.675	0.10	7	0.505	0.09	6	0.691	0.08	7	0.544	0.12	7	0.549	0.13	7	0.579	0.08	8	0.014	0.00	8	0.502	0.09	7	0.644	0.08	7	0.916	0.03	7	0.562	0.22
**A8b**	5	0.724	0.12	6	0.616	0.08	4	0.730	0.09	6	0.601	0.11	6	0.632	0.11	5	0.778	0.05	5	0.774	0.05	5	0.636	0.06	5	0.671	0.06	2	0.966	0.02	5	0.713	0.10
**A9**	8	0.633	0.14	*11*	*0.243*	*0.06*	8	0.642	0.11	*11*	*0.239*	*0.07*	9	0.486	0.15	8	0.566	0.09	7	0.317	0.04	7	0.519	0.09	*11*	*0.264*	*0.05*	6	0.958	0.02	8	0.487	0.22
**A0**	3	0.733	0.11	5	0.617	0.08	3	0.730	0.09	4	0.612	0.11	5	0.635	0.11	6	0.762	0.05	4	0.775	0.04	4	0.645	0.06	6	0.665	0.06	3	0.966	0.02	4	0.714	0.10
**L5 – Status quo scenario**																																	
**A1b**	3	0.796	0.09	3	0.757	0.07	3	0.776	0.08	3	0.751	0.10	3	0.767	0.10	3	0.873	0.03	3	0.884	0.04	3	0.790	0.04	**1**	**0.856**	**0.04**	**1**	**0.9637**	**0.02**	3	0.821	0.07
**A2**	4	0.763	0.09	4	0.727	0.07	4	0.745	0.08	4	0.699	0.10	4	0.724	0.10	4	0.839	0.04	4	0.843	0.04	4	0.770	0.04	4	0.822	0.04	4	0.958	0.02	4	0.789	0.07
**A3**	*11*	*0.606*	*0.08*	9	0.459	0.08	*11*	*0.587*	*0.08*	9	0.469	0.10	9	0.491	0.10	*11*	*0.433*	*0.09*	9	0.016	0.00	*11*	*0.457*	*0.09*	10	0.497	0.09	*11*	*0.815*	*0.06*	10	0.483	0.19
**A4**	7	0.661	0.11	8	0.494	0.10	8	0.654	0.09	8	0.530	0.12	8	0.512	0.13	9	0.519	0.09	7	0.605	0.08	7	0.525	0.08	8	0.559	0.10	8	0.924	0.03	7	0.598	0.12
**A5**	9	0.614	0.10	10	0.437	0.09	10	0.607	0.08	10	0.464	0.11	10	0.489	0.12	10	0.480	0.08	*11*	*0.001*	*0.00*	10	0.462	0.08	9	0.548	0.08	10	0.849	0.04	9	0.495	0.20
**A6**	2	0.799	0.08	**1**	**0.776**	**0.07**	**1**	**0.780**	**0.07**	2	0.773	0.09	2	0.780	0.09	2	0.883	0.03	2	0.902	0.03	**1**	**0.806**	**0.04**	2	0.855	0.04	6	0.951	0.02	2	0.831	0.06
**A6***	**1**	**0.800**	**0.08**	2	0.772	0.07	2	0.778	0.07	**1**	**0.775**	**0.09**	**1**	**0.781**	**0.09**	**1**	**0.883**	**0.03**	**1**	**0.905**	**0.03**	2	0.806	0.04	3	0.855	0.04	5	0.956	0.02	**1**	**0.831**	**0.06**
**A7**	8	0.660	0.10	7	0.511	0.09	7	0.689	0.08	7	0.542	0.12	7	0.533	0.13	7	0.574	0.08	10	0.010	0.00	8	0.510	0.09	7	0.642	0.08	9	0.913	0.03	8	0.559	0.22
**A8b**	6	0.686	0.11	6	0.578	0.08	6	0.715	0.09	6	0.569	0.11	6	0.587	0.11	6	0.718	0.05	6	0.725	0.04	6	0.601	0.06	6	0.650	0.06	3	0.959	0.02	6	0.679	0.11
**A9**	10	0.606	0.13	*11*	*0.228*	*0.06*	9	0.625	0.11	*11*	*0.227*	*0.07*	*11*	*0.465*	*0.14*	8	0.537	0.09	8	0.279	0.04	9	0.508	0.09	*11*	*0.261*	*0.05*	7	0.948	0.02	*11*	*0.468*	*0.22*
**A0**	5	0.743	0.11	5	0.644	0.08	5	0.733	0.09	5	0.652	0.11	5	0.672	0.11	5	0.791	0.05	5	0.782	0.04	5	0.691	0.06	5	0.712	0.07	2	0.959	0.02	5	0.738	0.09

References

1. Freiburghaus M. Personal communication. Information based on first insights of a study by Swiss Federal Gas and Water Industry Association about water demand in Swiss households. 2012.

2. SVGW. Wasserverbrauch im Haushalt. Fact sheet based on a joint study of SVGW (Swiss Federal Gas and Water Industry Association) and FOEN (Federal Office for the Environment), 2001.

3. BDEW. Trinkwasserverwendung im Haushalt 2011 (in German: Drinking water use in households 2011). Study by: Bundesverband der Energie- und Wasserwirtschaft BDEW (in German: German Association of Energy and Water Industries). Available from www.bdew.de (accessed 26 June 2011).

4. SVGW. Statistische Erhebungen der Wasserversorgungen in der Schweiz Betriebsjahr 2008 (in German: Statistical Assessments of Water Suppliers in Switzerland Operational Year 2008. Zürich: Schweizer Verein des Gas- und Wasserfaches (in German: Swiss Federal Gas and Water Industry Association), 2009.

5. Scholten L, Reichert P, Schuwirth N, Lienert J. Tackling uncertainty in multi-criteria decision analysis- An application to water supply infrastructure planning. European Journal of Operational Research. 2015;242(1):243-60. doi: 10.1016/j.ejor.2014.09.044.

6. Scholten L, Scheidegger A, Reichert P, Maurer M, Lienert J. Strategic rehabilitation planning of piped water networks using multi-criteria decision analysis. Water Research. 2014;49(1):124-43. doi: 10.1016/j.watres.2013.11.017.

7. Bosshard T, Kotlarski S, Ewen T, Schär C. Spectral representation of the annual cycle in the climate change signal. Hydrology and Earth System Sciences. 2011;15(9):2777-88.

8. CH2011. Swiss Climate Change Scenarios 2011. Zurich, Switzerland: 2011.

9. Integrated river water quality management. Project of the National Research Program NRP 61 [Internet]. 2013. Available from: http://www.nfp61.ch/E/projects/cluster-water-management/integrated-river_water-quality_management/Pages/default.aspx (accessed 06 December 2013).

10. Kilsby CG, Jones PD, Burton A, Ford AC, Fowler HJ, Harpham C, et al. A daily weather generator for use in climate change studies. Environmental Modelling & Software. 2007;22(12):1705-19. doi: http://dx.doi.org/10.1016/j.envsoft.2007.02.005.

11. Fatichi S, Ivanov V, Caporali E. AWE-GEN: Advanced weather generator technical reference version 1.0. University of Michigan, Ann Arbor, Michigan, USA; 2011.

12. Vince F, Aoustin E, Breant P, Marechal F. LCA tool for the environmental evaluation of potable water production. Desalination. 2008;220(1-3):37-56. doi: 10.1016/j.desal.2007.01.021. PubMed PMID: WOS:000254024800004.

13. Katsoyiannis IA, Canonica S, von Gunten U. Efficiency and energy requirements for the transformation of organic micropollutants by ozone, O3/H2O2 and UV/H2O2. Water Research. 2011;45(13):3811-22. doi: 10.1016/j.watres.2011.04.038.

14. Fortschreibung und Erweiterung Daten- und Rechenmodell. Energieverbrauch und Schadstoffemissionen des motorisierten Verkehrs in Deutschland 1960 - 2030 (TREMOD, Version 5) (in German: Continuation and extention of the data and calculation model. Energy use and pollutant emissions of motorized transport in Germany 1960-2030). UBA, Umweltbundesamt. 2010. Available from: http://www.probas.umweltbundesamt.de/php/themen.php?&prozessid={6D34A996-4DCC-7061-9B52-3006450AB34A}&id=13455327232&step=4&search= (accessed 11 July 2013).

15. Jones DS, Efroymson RA, Armstrong AQ, Muhlheim MD, Carnes SA. Integrated Risk Assessment for Individual Onsite Wastewater Treatment Systems. St. Louis: Oak Ridge National Laboratory, 2004 Contract No.: Prepared for the National Decentralized Water Resources Capacity Development Project, Project No. WU-HT-01-18.

16. Grundwasserqualität - Messdaten (in German: Measurement data on groundwater quality) [Internet]. Amt für Abfall, Wasser, Energie und Lust des Kantons Zürich (Office for waste, waste, energy, and air of the canton of Zurich). 2013. Available from: http://www.awel.zh.ch/internet/baudirektion/awel/de/wasserwirtschaft/grundwasser/messdaten_gw/gw_quali.html#a-content (accessed 18 August 2013).

17. Stadt Zürich. Jahresbericht 2011: Seewasser-Untersuchungen (in German: Annual Report 2011: Lakewater analyses). 2012.

18. European Foundation for Quality Management [Internet]. 2012. Available from: http://www.efqm.org/en (accessed 6 November 2012).

19. Lienert J, Scholten L, Egger C, Maurer M. Structured decision-making for sustainable water infrastructure planning and four future scenarios. EURO Journal on Decision Processes. 2015;3(1-2):107-40. doi: 10.1007/s40070-014-0030-0.

20. Vreeburg JHG, Blokker EJM, Horst P, Van Dijk JC. Velocity-based self-cleaning residential drinking water distribution systems. Water Science and Technology: Water Supply. 2009;9(6):635-41.

21. Eisenführ F, Weber M, Langer T. Rational Decision Making. 1st ed: Springer; 2010.
